# Supplementary material for: Heart Rehabilitation in patients awaiting Open heart surgery targeting to prevent Complications and to improve Quality of life (Heart-ROCQ): study protocol for a prospective, randomised, open, blinded endpoint (PROBE) trial
Source: BMJ Open. 2019 Sep 18;9(9):e031738. doi: 10.1136/bmjopen-2019-031738 (PMC6756317; doi:10.1136/bmjopen-2019-031738)
Supplement: Supplementary data [file bmjopen-2019-031738supp001.pdf]

SUPPLEMENTARY INFORMATION - DESCRIPTION OF THE HEART-ROCQ PROGRAMME

PRE-out phase – An outpatient cardiac rehabilitation phase during the waiting period
Three times per week, a minimum of three weeks

Physical therapy

Aims
- To maintain or improve patients physical capacity before surgery
- Patient learns to apply the stress-strain training principles
- To optimize pulmonary muscle strength
- Patient knows which breathing and coughing techniques should be used after surgery to prevent pulmonary complications

When needed:
- Patient learns to recognize body signals and boundaries.
- Patient is able to exercise despite of possible kinesiophobia

Type of exercise	Frequency	Intensity	Monitoring
IMT	3 × p / wk	- 6 cycles of 6 repetitions,<br>- rest periods of resp. 60, 45, 30, 15 and 5 s.<br>- 60-80% of maximal inspiratory pressure[65]	- Week 1: each training ↑ intensity with 10%<br>- Intensity ↑ with 5% if RPE <5[1]
Aerobic cycle	3 × p / wk	- 25 min. at RPE 3[1]<br>- Interval training will be given, if the patient is not able to perform endurance training.	- First training: 50% of POpeak<br>- Load ↑ if RPE< 3[1]<br>- Interval training: guided by complaints and RPE of 3
Resistance training	3 × p / wk	- 1-3 cycles of 10-15 repetitions<br>- Rest: 30-60 s.<br>- 50-80% of estimated 1RM<br>- On six fitness apparatuses	- First training: 6-10 RM per fitness apparatus
Body awareness	1 × p / 2 wks	- 30 min. breathing and relaxation techniques	Not Applicable
Group education	Two sessions	- Basic training principles<br>- Forced expiration, huff and cough techniques	Not Applicable

Dietary advice[2]

Aims
- To maintain or improve patients nutritional status
- Patient knows which nutrients are important focusing on the surgery
- Patient knows the importance of food in relation to cardiovascular disease and cardiovascular risk management

Type	Frequency	Content
Intake interview	One session	- Anamnesis about nutritional status
Counselling	On indication	- Individual sessions according to existing guidelines
Group education	One session	- 60 min., cardiovascular risk factors and dietary intake

Psychological guidance[2]

Aims
- To optimize mental status of the patients before surgery
- Patient has made a start with the awareness of cardiovascular risk factors

Intake interview	One session	- Anamnesis about mental status
Counselling	On indication	- Individual sessions according to evidence-based treatment protocols dependent on mental problems of patient[3]
Group education	One session	- Coping with psychological tension/stress, to process the mental trauma of cardiac surgery, and risk factors

No-smoking consultation *(For patients who smoke)*

Aim
- Patient gives up smoking during the waiting time before surgery

Intake interview & counselling	One session per wk	- 30 min., individual session based on existing guidelines

IMT: Inspiratory muscle training; RPE: Rate of perceived exertion; POpeak: Maximum power output achieved during submaximal ergometry test. [1]On a Borgscale 0-10; [2]Involvement of partner/relatives during group and individual sessions; [3] like cognitive behavioural therapy, eye movement desensitisation and reprocessing (EMDR), acceptance and commitment therapy etc.

Hartog J, *et al. BMJ Open* 2019; 9:e031738. doi: 10.1136/bmjopen-2019-031738

POST-in phase – An inpatient cardiac rehabilitation phase
Starting 4-7 days after surgery, duration of three weeks, weekends at home

Physical therapy

Aim

- To recover patients physical capacity
- Patient performs breathing and coughing techniques to prevent pulmonary complications
- Patient mobilise and can perform activities of daily living independently
- Patient knows about risk factors according to physical activity, knows the ACSM recommendations (moderate-intensity cardiorespiratory exercise training for ≥30min/day ≥5 days/week or vigorous-intensity cardiorespiratory exercise training for ≥20 min/day on ≥3 days/week or a combination[66]) and makes a plan to apply the ACSM recommendations in his own life
- Patient works on personal goals

Type of exercise	Frequency	Intensity	Monitoring
Individual therapy	2 × p / day	- Practice at transfers, walking, and climbing stairs<br>- Very light mobilising exercises for upper extremity	- During the first 2 days of this phase.<br>- Extended, if patient is not able to participate in the group sessions.
Individual therapy	2 × p / wk	- Attention to personal goals	- Week 2 and 3
IMT[2]	3 × p / wk: 2 × under supervision, 1 × without supervision	- 6 cycles of 6 repetitions,<br>- rest periods of resp. 60, 45, 30, 15 and 5 s.<br>- intensity of 60-80% of maximum inspiratory pressure[65]	- First training: 50% of resistance of last preoperative training<br>- Intensity ↑ with 5% if RPE <5[1]<br>- IMT stops when resistance of preoperative training is reached
Aerobic cycle[2]	1 × p / day	- Week 1: duration of 5-20 min. at light intensity (RPE 2[1])<br>- Week 2 and 3: Work up to 25 min. at moderate intensity (RPE 3[1])<br>- Interval training will be given, if the patient is not able to perform endurance training.	- First training: at 50% of power output of last preoperative training<br>- Load ↑ if RPE< 2 à 3[1]<br>- Interval training: guided by complaints and RPE of 3.
Resistance training[2]	3-4 × p / wk	- 3 cycles of 15-20 repetitions<br>- Rest periods of 30-60 seconds<br>- On six fitness apparatuses	- First training: 50% of resistance of last preoperative training for LE and 25% for UE.<br>- Gradual build up to 50-80% van 1RM based on RPE 3[1]
Body awareness[2]	1 × p / wk	- 30 min. breathing and relaxation techniques	Not Applicable

Dietary advice[3]

Aim

- To maintain or improve patients nutritional status
- Patient knows which nutrients are important focusing on the recovery of surgery
- Patients knows the importance of food in relation to cardiovascular disease and cardiovascular risk management

Type	Frequency	Content
Intake interview	One session	- Anamnesis about nutritional status post-surgery
Counselling	Sessions on indication	- Treatment according to existing guidelines
Group education	One session	- 90 min., skills needed to maintain a healthy life style

Psychological guidance[3]

Aim

- Patient start to process the mental trauma of cardiac surgery and the consequences of it
- Patient has self-management competence to maintain a healthy life style
- Patient and partner/relatives are able to support each other in the process

Intake interview	One session	- Anamnesis about mental status post-surgery
Intake interview & counselling	Sessions on indication	- Individual sessions according to evidence-based treatment protocols dependent on mental problems of patient[4]
Group education	One session	- 90 min., processing the cardiac surgery: possible reactions en consequences, coping with stress and healthy life style

IMT: Inspiratory muscle training; RPE: Rate of perceived exertion; LE: Lower extremities; UE: Upper extremities. [1]On a Borgscale 0-10; [2]Group sessions start from day three of the inpatient phase or when patient is able to perform in the group activities; [3]Involvement of partner/relatives during group and individual sessions; [4] like cognitive behavioural therapy, eye movement desensitisation and reprocessing (EMDR), acceptance and commitment therapy etc.

Hartog J, *et al. BMJ Open* 2019; 9:e031738. doi: 10.1136/bmjopen-2019-031738

POST-in phase – An inpatient cardiac rehabilitation phase (continuation)<br>*Starting 4-7 days after surgery, duration of three weeks, weekends at home*		
**No-smoking consultation**[1] *(For patients who smoke)*		
Aim<br>- Patient is still motivated to continue to stop smoking or patient is motivated to give up smoking (when patient did not give up smoking before cardiac surgery.		
Intake interview & counselling	1 × p / wk	- 30 min., individual sessions based on existing guidelines
Group education	One session	- 60 min., general information about smoking addiction and support from fellow smokers who also like to give up smoking
**Return to work consultation**[1] *(for patients who are employed)*		
Aim<br>- Patient is informed about laws and regulation for illness, social security contributions, and medical examinations<br>- Patient knows their rights and obligations, the rights and obligations of the employer, the working conditions agency, the employee insurance agency, and re-integration companies<br>- Patient received tools to return adequately back to work (knows positive and negative factors that can impact the re-integration)		
Group education	One session	- 60 min., laws and regulation for illness, procedures, roles, rights and obligations of different involved persons, communication to involved persons (e.g. colleagues, employers), working during rehabilitation, positive and negative factors regarding to return to work
Counselling	Sessions on indication	- Individual sessions with labour consultant dependent on problems of patient

[1]Involvement of partner/relatives during group and individual sessions.

**POST-out phase – An outpatient cardiac rehabilitation phase**<br>*Starting on Tuesday after discharge of the POST-in phase, two times per week, four weeks*			
**Physical therapy**			
Aim<br>- Patient has optimized his/her physical capacity<br>- Patient knows his/her boundaries and limitations<br>- Patient knows about cardiovascular risk factors according to physical activity, knows the ACSM recommendations (moderate-intensity cardiorespiratory exercise training for ≥30min/day ≥5 days/week or vigorous-intensity cardiorespiratory exercise training for ≥20 min/day on ≥3 days/week or a combination[65]) and makes a plan to apply the ACSM recommendations in his own life<br>- Patient resumes his/her work or hobbies<br>- Patient experiences pleasure during exercise<br>- Patients achieves their personal goals			
**Type of exercise**	**Frequency**	**Intensity**	**Monitoring**
Aerobic cycle	2 × p / wk	Depending on trainability of patient<br>- 25 min. at 50-80% HRR or RPE 5[1]	- Work up to 25 minutes at moderate intensity (RPE 3[1]), when patient was not able to do it in POST-in phase<br>- Load ↑ if RPE< 5[1] or HRR <50-80%
Resistance training	2 × p / wk	UE:<br>- 3-4 cycles of 10-15 repetitions<br>- Rest: 30-60 s.<br>- 50-80% of estimated 1RM<br>- On six fitness apparatuses<br>LE:<br>- Focus on mobilization in week 1 & 2<br>- Focus on strength and endurance in week 3 & 4	- UE: Load ↑ if RPE< 5[1]<br>- LE: Under guidance of complaints
Sport and games	1 × p / wk	Focus on:<br>- Experiencing pleasure during exercise, regaining trust and handling boundaries.<br>- Exploring different types of sports and knowing the possibilities after CR	
Swimming	1 × p / wk		
Education	1 × p / wk	- Training principles of POST-out phase and a repetition of ACSM recommendations<br>- Awareness of exercise after CR; making a plan to exercise after CR<br>- Explanation of the results of the exercise test<br>- Discussing the plan to exercise, share experiences to expand exercise in home situation	
**Dietary advice, psychological guidance, no-smoking consultation, and return to work consultation**			
Individual sessions are continued when aims are not achieved			

RPE: Rate of perceived exertion; HRR: Heart rate reserve; LE: Lower extremities; UE: Upper extremities; ACSM: American College of Sports Medicine; CR: Cardiac Rehabilitation. [1]On a Borgscale 0-10; [2]Involvement of partner/relatives during group and individual sessions.
