# Supplementary material for: Heart Rehabilitation in patients awaiting Open heart surgery targeting to prevent Complications and to improve Quality of life (Heart-ROCQ): study protocol for a prospective, randomised, open, blinded endpoint (PROBE) trial
Source: BMJ Open. 2019 Sep 18;9(9):e031738. doi: 10.1136/bmjopen-2019-031738 (PMC6756317; doi:10.1136/bmjopen-2019-031738)
Supplement: Author's manuscript [file bmjopen-2019-031738.draft_revisions.pdf]

BMJ Open is committed to open peer review. As part of this commitment we make the peer review history of every article we publish publicly available.

When an article is published we post the peer reviewers' comments and the authors' responses online. We also post the versions of the paper that were used during peer review. These are the versions that the peer review comments apply to.

The versions of the paper that follow are the versions that were submitted during the peer review process. They are not the versions of record or the final published versions. They should not be cited or distributed as the published version of this manuscript.

BMJ Open is an open access journal and the full, final, typeset and author-corrected version of record of the manuscript is available on our site with no access controls, subscription charges or pay-per-view fees (http://bmjopen.bmj.com).

If you have any questions on BMJ Open's open peer review process please email info.bmjopen@bmj.com

HEART Rehabilitation in patients awaiting Open-heart surgery to prevent Complications and to improve Quality of life (Heart-ROCQ): a Prospective Randomised Open Blinded End-point (PROBE) trial

Journal:	*BMJ Open*
Manuscript ID	bmjopen-2019-031738
Article Type:	Protocol
Date Submitted by the Author:	17-May-2019

Complete List of Authors:	Hartog, Johanneke; UMCG<br>Blokzijl, Fredrike; UMCG, Cardio-thoracic Surgery<br>Dijkstra, Sandra; UMCG, Cardio-thoracic Surgery<br>DeJongste, Mike; UMCG<br>Reneman, Michiel; UMCG, Rehabilitation Medicine<br>Dieperink, Willem; UMCG, Critical Care<br>van der Horst, Iwan; UMCG, Critical Care<br>Fleer, Joke; UMCG, Health Psychology<br>van der Woude, Lucas; UMCG, Center for Human Movement Sciences; UMCG, Rehabilitation Medicine<br>Van der Harst, Pim; UMCG, Cardiology<br>Mariani, Massimo; UMCG, Cardio-thoracic surgery

Keywords:	rehabilitation, cardiac surgical procedures, life style, health status, postoperative complications, cost-benefit analysis

SCHOLARONE™
Manuscripts

Trial design BMJ Open

Title

HEART Rehabilitation in patients awaiting **O**pen-heart surgery to prevent **C**omplications and to improve **Q**uality of life (Heart-ROCQ): a **P**rospective **R**andomised **O**pen **B**linded **E**nd-point (PROBE) trial

Authors

Johanneke Hartog, MSc[a]; Fredrike Blokzijl, MSc[a]; Sandra Dijkstra, MSc[a]; Mike J.L. DeJongste, M.D., PhD, FESC[b]; Michiel F. Reneman, PhD[c]; Willem Dieperink, PhD[d]; Iwan C.C. van der Horst, M.D., PhD[d]; Joke Fleer, PhD[e]; Lucas H.V. van der Woude, PhD[c,f]; Pim van der Harst, M.D., PhD[g]; Massimo A. Mariani, M.D., PhD[a]

a University of Groningen, University Medical Centre Groningen, Department of Cardio-thoracic Surgery, Groningen, The Netherlands

b University of Groningen, University Medical Centre Groningen, Groningen, The Netherlands

c University of Groningen, University Medical Centre Groningen, Department of Rehabilitation Medicine, Groningen, The Netherlands

d University of Groningen, University Medical Centre Groningen, Department of Critical Care, Groningen, The Netherlands

e University of Groningen, University Medical Centre Groningen, Department of Health Psychology, Groningen, The Netherlands

f University of Groningen, University Medical Centre Groningen, Centre for Human Movement Sciences, Groningen, The Netherlands

g University of Groningen, University Medical Centre Groningen, Department of Cardiology, Groningen, The Netherlands

Corresponding Author:

Johanneke Hartog, Department of Cardiothoracic Surgery, University Medical Centre Groningen (UMCG), Hanzeplein 1, AB41, 9713 GZ Groningen, The Netherlands

Phone: +3150 3617527; Email: j.hartog@umcg.nl; FAX: +31503614391

For peer review only

For peer review only - http://bmjopen.bmj.com/site/about/guidelines.xhtml

Abstract

Introduction

The rising prevalence of modifiable risk factors (e.g. obesity, hypertension, and physical inactivity) is causing an increase in possible avoidable complication rates in patients undergoing cardiac surgery (CS). This study therefore aims to assess whether a combined pre- and postoperative multidisciplinary cardiac rehabilitation (CR) program (Heart-ROCQ program) can improve functional status and reduce surgical complications, readmissions and major adverse cardiac events (MACE) as compared to standard care.

Methods and analysis

Patients (n=350) are randomised to the Heart-ROCQ program or standard care. The Heart-ROCQ program consists of a preoperative optimization phase during the waiting time (3 times p/week, minimal 3 weeks), a post-operative inpatient phase (3 weeks) and an outpatient CR phase (2 times p/week, 4 weeks). Patients receive multidisciplinary (e.g. physical, dietary, psychological, and smoking cessation) treatment. Standard care consists of 6 weeks post-surgery outpatient CR with education and physical therapy (2 times p/week). The primary outcome is a composite weighted score of functional status, surgical complications, readmissions, and MACE and is evaluated by a blinded end-point committee. Secondary outcomes are length of stay, physical, and psychological functioning, life style risk factors, and work participation. Finally, an economic evaluation is performed. Data is collected at six time points: at baseline (start of the waiting period), the day before surgery, at time of discharge from the hospital, and at 3, 7, and 12 months after surgery.

Ethics and dissemination

This study will be conducted according to the principles of the Declaration of Helsinki (version 8, October 2013). The protocol has been approved by the Medical Ethical Review Board (METc) of the UMCG (No. 2016/464). Results of this study are submitted to a peer-reviewed scientific journal and can be presented at national and international conferences.

For peer review only - http://bmjopen.bmj.com/site/about/guidelines.xhtml

For peer review only

Registration: The Heart-ROCQ study has been registered at ClinicalTrials.gov (No. NCT02984449).

Strength and limitations of this study

- A prospective randomised open, blinded end-point (PROBE) trial
- Comparison of a unique multidisciplinary pre- and post-operative CR program with a standard care post-operative outpatient CR program
- Patient-oriented: primary and secondary outcomes provide a comprehensive and clinically relevant evaluation of patients' recovery from surgery (e.g. on physical, psychological, social domains)
- Both short- and long term effects of the CR programs are included
- Single centre study, albeit that patients are referred from four hospitals

For peer review only

For peer review only - http://bmjopen.bmj.com/site/about/guidelines.xhtml

INTRODUCTION

The leading cause of death in Western countries is ischemic heart disease.(1) One of the treatments for severe ischemic heart disease is cardiac surgery (CS). The risk of complications related to CS is substantial; post-operatively, pulmonary complications (up to 33%), delirium (~26%), and arrhythmias (~30%) have been reported to occur.(2–4) In turn, these complications are associated with prolonged hospitalisation, increased adverse events (i.e. readmission, stroke, myocardial infarction, and mortality), reduced health related quality of life (HRQoL), and higher health care costs.(5–10) Patients with poor dietary habits (present in ~80% of the candidates for CS), who are physical inactive (~45%), who smoke (22%), or who experience depression and/or anxiety disorders (~30%) are at higher risk for post-operative complications and are at risk for lack of functional benefits after CS.(8,11–19)

Over the last decades risk factors such as age, obesity, diabetes, hypertension and dyslipidemia have steadily increased in patients undergoing CS.(20,21) Yet, despite their adverse effects on treatment outcomes, reducing the burden of modifiable risk factors are currently not part of standard clinical care before and after CS. Before CS, patients often have a preoperative period of several weeks on the waiting list in which they receive little or no guidance. This waiting period has been associated with increased psychological stress, feelings of anxiety and reduced functional status.(22,23) With respect to inactivity during hospitalisation after CS, research has shown that during the 8 to 11 days of hospitalisation, patients spent the majority of time in sitting or supine position.(24,25) In-hospital physical inactivity is a predictor of a longer hospital stay and re-hospitalisation.(24,26,27) It causes a decrease in muscle strength and aerobic capacity, both fundamental in the performance of activities of daily living.(28,29) This reduced physical capacity may seriously impact CS patients' independence, especially since these patients are often elderly and thus the functioning of their entire physiological system is already reduced.(30)

Cardiac rehabilitation (CR) targets to improve the pre- and post-operative status of patients undergoing CS. CR has the aim "to favourably influence the underlying cause of cardiovascular disease, as well as to provide the best possible physical, mental, and social conditions".(31) Post-

For peer review only - http://bmjopen.bmj.com/site/about/guidelines.xhtml

operative CR is already an essential part of standard care in the Netherlands, although many hospitals in the Netherlands provide a phase II outpatient CR program, starting 3 to 6 weeks after CS.(32,33) Benefits of post-operative CR are reported for a variety of cardiac patients,(34) however the evidence in patients undergoing CS is still lacking with regard to patient-relevant outcomes and mortality.(35) In addition to post-operative CR, small trials suggested that preoperative CR is effective in reducing post-operative pulmonary complications, duration of hospital stay, improving HRQoL, and increasing the compliance to post-operative CR.(23,36) However, long-term effects and the effects on other complications remain unclear. Furthermore, most studies investigated the effect of preoperative CR *or* post-operative CR, but not the effect of both CR methods combined. The hypothesis is that a combined pre- and post-operative CR program is more beneficial when compared to a separate preoperative CR program *or* a single post-operative CR program.

The aim of the Heart-ROCQ study is to determine the effect of a comprehensive pre- and post-operative CR program (the Heart-ROCQ program) on functional status, post-operative surgical complications, readmissions to the hospital, and major adverse cardiac events (MACE) compared to a regular Dutch post-operative outpatient CR program (the Standard Care CR program).(32) In addition, the effect of the Heart-ROCQ program on physical and psychological functioning, including HRQoL, life style risk factors, work participation, and cost-effectiveness are evaluated in comparison to the Standard Care CR program. To assess for whom and why the CR programs are effective, moderator and mediator analyses are performed.

For peer review only - http://bmjopen.bmj.com/site/about/guidelines.xhtml

METHODS AND ANALYSIS

Study design and organization

This investigator-initiated prospective randomised open, blinded end-point (PROBE) trial is executed in a single Centre (University Medical Centre Groningen [UMCG]). Patients from three referral hospitals (Ommelander Hospital Groningen [OZG], Martini Hospital Groningen [MZH], and Wilhelmina Hospital Assen [WZA]) who are accepted for CS at the UMCG are also approached to participate. Patients are randomly assigned to a combined pre- and post-operative multidisciplinary CR program (the Heart-ROCQ group) or a regular phase II outpatient CR program after surgery (the Standard Care group).(32) Figure 1 provides an overview of the study design.

Ethical considerations and dissemination

This study is conducted according to the principles of the Declaration of Helsinki (version 8, October 2013) and according to the research code of the UMCG. It has been registered at ClinicalTrials.gov (No. NCT02984449) and the protocol (version 2, 2016-Dec-19) has been approved by the Medical Ethical Review Board (METc) of the UMCG (No. 2016/464). If applicable, substantial amendments will be notified to the METc for approval and changes will be written in the articles describing the results of the study. Results of this study will be submitted to a peer-reviewed scientific journal and can be presented at national and international conferences. In addition, the results will be distributed by various information channels (e.g. websites of cardiac patient organisations, social media). Two to three times a year a newsletter about the progress and (in the end) the results of the study is sent to participants, who are interested.

Patient and public involvement

In a pilot study (to be submitted) the patient satisfaction and the feasibility (in terms of accessibility, compliance, training load and safety) of the Heart-ROCQ program have been evaluated. These results were taken into account when further developing the Heart-ROCQ program and the protocol of this study.

For peer review only - http://bmjopen.bmj.com/site/about/guidelines.xhtml

Funding

The Heart-ROCQ study is financially supported by Edwards Lifesciences SA, Abbott (former St. Jude Medical Nederland B.V.) and ‘Stichting Beatrixoord Noord-Nederland’. The sponsors are not involved in the design or execution of the study.

Participants

Patients (≥ 18 years) admitted to elective coronary artery bypass grafting (CABG), valve surgery, aortic surgery, or combined procedures are eligible. Patients accepted for congenital procedures, transcatheter aortic valve implantations (TAVI), aortic dissections, or aortic descending repair are excluded. Other exclusion criteria are: being unable to participate in all program elements of the Heart-ROCQ program due to disorders of the nervous or musculoskeletal system that limits exercise capacity, chronic obstructive pulmonary disease (COPD) with Global Initiative for Chronic Obstructive Lung Disease (GOLD) criteria classification 3 or 4,(37) addiction to alcohol or drugs, a serious psychiatric illness (i.e. recently experienced psychosis, bipolar disorder, diagnosis of schizophrenia, serious cognitive, or neurological problems, and acute suicidal ideations or behaviour), or when it is undesirable to exercise (i.e. hypertrophic cardiomyopathy, unstable angina, advice from cardiologist); any treatment which is planned during one of the CR programs and which is expected to interrupt attendance to the CR program (e.g. planned organ transplantation, preoperative endocarditis, or planned chemotherapy etc.); playing a sport at (inter)national level; being unable to read, write, or understand Dutch.

Study enrolment, randomization and registry

Figure 2 shows the flow chart of the Heart-ROCQ study. Patients on the waiting list of the thoracic surgery department and meeting the study criteria for type of surgery are asked to participate by their cardiologist. The cardiologist provides the patients with study information and an invitation to meet the researcher at the preoperative consultation. At the preoperative consultation, the researcher will obtain informed consent and conduct the baseline measurements. Eligible patients who have signed informed consent and performed the baseline measurements, are randomised to the Heart-ROCQ

For peer review only - http://bmjopen.bmj.com/site/about/guidelines.xhtml

group or Standard Care group. Randomization (concealed group allocation in REDCap, random blocks of 2-4, 1:1 ratio) is stratified for weight of the surgery (isolated CABG, single non-CABG [i.e. replacement or repair of part of aortic or valve], 2 procedures or 3 procedures), gender, and age (≥65 and <65 years). Prior to the start of the study, the randomization lists were created (using the 'ralloc' function of Stata/SE, version 13.0) and imported into the secure data collection tool REDCap (version 7.3.2), by an independent researcher. Medical staff and researchers are not blinded to group allocation, because of logistic reasons. The primary end point is evaluated by an independent end-point committee, blinded for group allocation.

Patients who are not willing to participate are asked to give written consent for using data that are collected during routine care. These data are collected in the Heart-ROCQ study registry to get more insight in potential differences between patients who participated in the study and patients who did not. The Heart-ROCQ registry will thus provide more insight in the generalizability of the results. Data of this registry are not used for the primary statistical analyses.

For peer review only

For peer review only - http://bmjopen.bmj.com/site/about/guidelines.xhtml

Intervention

Heart-ROCQ group

The Heart-ROCQ group receives a CR program at the Centre of Rehabilitation of the UMCG (location Beatrixoord, which is located 6 km from the hospital of the UMCG) consisting of 3 phases. The first phase is an outpatient preoperative optimization phase during the waiting period (3 times per week, minimum of 3 weeks). The second phase is a post-operative inpatient CR phase (3 weeks, only during weekdays) followed by the third phase, an outpatient CR phase (2 times per week, for 4 weeks). During each phase, all participants receive physical therapy including group sessions of inspiratory muscle training (IMT),(38) strength training, aerobic cycling, breathing, coughing and relaxation. In addition, patients have an intake with a dietician and a psychologist and take part, on indication, in individual sessions to optimize their health. Moreover, different group education sessions are organized regarding coping with stress, awareness of risk factors, and maintaining a healthy life style. Two additional modules, namely coaching to stop smoking and to return to work, are available for patients who respectively smoke or are employed. A detailed description of the CR program is given in the supplementary information.

Standard Care group

In the Netherlands, the current standard of care consists of a phase II outpatient CR program, which is conducted at the referred hospital.(32,33) In general, this CR program starts 3 to 6 weeks after discharge from the hospital and lasts for 6 weeks. The program consists of four educational sessions (regarding risk factors and retaining a healthy life style) and physical therapy (2 times per week: 30 min. cycling together with 30 min. sports and games, relaxation therapy, or strength training). Furthermore, patients can be referred to a psychologist or a dietician when needed. The content of this CR program is based on the Dutch CR guidelines.(39)

For peer review only - http://bmjopen.bmj.com/site/about/guidelines.xhtml

Composite primary end point: functional status, complications and events

The primary outcome is a composite weighted score of functional status, post-operative surgical complications, readmissions to the hospital, and MACE. Each event is scored (ranging from 1 to 3 points), separately. Table 1 shows an overview of the primary outcome and the scoring system.

The scores of all events are summed up to calculate a total score. Only the most serious complication is counted per event (e.g. when a percutaneous coronary intervention [score 1] is complicated by a stroke [score 2], the score will be 2 and not 3 [1 + 2]).

The concept of the composite weighted score is adapted from the African-American Heart Failure Trial (A-HeFT).(40) Functional status is assessed through two health domains of the Medical Outcome Study 36-item General Health Survey (RAND-36 version 2)(41): physical functioning and physical health problems. The primary end point is evaluated at 3 months (T4) and 1 year (T6) after surgery (Figure 1). Deep wound infections and surgical re-explorations are screened up to 120 days, after surgery. Other post-operative surgical complications are measured in the period between the surgery and when a patient meets the UMCG discharge criteria (Table 2). Hospital admissions are checked between baseline and one year after surgery. To prevent bias (since the Heart-ROCQ group follows the in-patient CR phase after surgery) hospital admissions between the day of admission before surgery and 30 days after surgery are not included when determining the (calculated) primary end point.

Secondary outcomes

Complications and events

All individual components of the composite end point regarding the complications and events will be analysed separately as secondary outcomes. Table 3 summarizes the definitions of the secondary complications and events, including the moments of screening.

All documents concerning the composite primary end point and the secondary complications are encrypted and subsequently adjudicated by the independent end-point committee. The end-point committee consists of four members (cardiologists and cardiothoracic surgeons), who are not employed in the UMCG and are blinded for group allocation.

For peer review only - http://bmjopen.bmj.com/site/about/guidelines.xhtml

Table 1: definitions and score of the components of the composite primary end point

**Functional status**	**Score**
Worsening in physical functioning (*domain score of health related quality of life, rand36_v2)*†	1
No change or improvement in physical functioning†	0
Worsening in physical problem (*domain score of health related quality of life, rand36_v2)*†	1
No change or improvement in physical problem†	0
A clinical relevant worsening is classified as minimal change according to *Wyrwich et al., 2004*(42)	
**(Serious) Adverse Events**	
No serious adverse event	0
Prolonged mechanical ventilation<br>*Mechanical ventilation longer than 24 hours*	1
Lung infection<br>*1) A new lung radiographic infiltrate AND 2) two signs that the infiltrate is infectious origin (i.e. a) fever - body temperature >38°C or <36°C -, b) leukocytosis - white blood cells >10,000 or <4000, c) positive sputum culture AND/OR d) decline in oxygenation*	1
Delirium<br>*1) A DOS score ≥ 3 at hospital ward AND/OR 2) Diagnosis confirmed by a psychiatrist, geriatrist, or supervising specialist according to the DSM-IV criteria resulting in treatment with medication*	1
Readmissions to intensive care unit<br>*Unrelated to a secondary end point*	1
Deep wound infection<br>*Deeper tissues are affected (muscle, sternum, and mediastinum) and must include: 1) surgical drainage / re-fixation OR 2) an organism is isolated from culture of mediastina tissue or fluid, OR 3) antibiotic treatment, because of sternum wound*	2
Readmissions to hospital<br>*An unplanned hospital stay with different dates of admission and discharge with a medical indication (i.e. clinical signs and symptoms or change of treatment)*	1
Any cardiothoracic surgical interventions<br>*Graft- or valve failure, CABG, valve, aortic, or other cardiac surgery*‡	2
Any percutaneous interventions<br>*PCI, TAVI, etc.*	1
Myocardial infarction<br>*According to the third universal definition of myocardial infarction*§	2
Cerebral vascular accident / stroke<br>*Acute neurological event of at least 24 hours of duration, with focal signs and symptoms and without evidence supporting any alternative explanation. Diagnosis of stroke requires confirmation by CT, MRI, or pathological confirmation.*	2
Sudden death survivor<br>*The sudden onset of symptoms, such as chest pain and cardiac arrhythmias, ventricular tachycardia, which lead to the loss of consciousness and cardiac arrest followed by reanimation and does not lead to biological death.*	2
Death<br>*All-cause mortality*	3
Total score = *sum of physical status and SAE's at 3 months and 1 year (worst score of each event)*	

†Compared with baseline; ‡According to the definitions of the 'Begeleidingscommissie Hartinterventies Nederland; §Thygesen et al., 2012(43); DOS: Delirium Observation Screening scale(44); CABG: Coronary arterial bypass graft; PCI: Percutaneous coronary intervention; TAVI: Transcathetic aortic valve implantation; In gray: post-operative complications can be scored ones; SAE: Serious adverse event.

For peer review only

For peer review only - http://bmjopen.bmj.com/site/about/guidelines.xhtml

Table 2: Discharge criteria of the University Medical Centre Groningen (UMCG)

1) no drain, no external pacemaker lead, no infusion, or oxygen present,

2) stable clinical conditions (stable lab results, X-ray, and haemodynamic parameters),

3) able to perform basic activity of daily living (ADL)-activities (i.e. going independently to the toilet).

Table 3: definitions of the secondary complications and events

**Definitions**	**Time of measure**
Atrial fibrillation<br>*New onset of atrial fibrillation or atrial flutter requiring medical treatment or cardioversion*	Surgery to T3
Prolonged ICU stay<br>*When the number of calendar days is two or more from ICU admission to discharge*	Initial stay
Readmissions to hospital<br>*The number of unplanned hospital stays with different dates of admission and discharge with a medical indication (i.e. clinical signs and symptoms or change of treatment)*<br>*a) directly related to a cardiac cause*<br>*b) not directly related to a cardiac cause*	Baseline to T6[§]
Hospitalisation days<br>*Total number of days of hospitalisation*<br>*a) directly related to a cardiac cause*<br>*b) not directly related to a cardiac cause*	Baseline to T6[§]
Cardiovascular death<br>*Any death due to proximate cardiac or cardiovascular cause (e.g. Myocardial infarct, low-output failure, fatal arrhythmia, death secondary to a cerebral vascular accident, pulmonary embolism, ruptured or dissecting aortic aneurysm, or other vascular diseases), un-witnessed death, and death of unknown cause, and all procedure related deaths (e.g. PCI, CABG), including those related to concomitant treatment[†].*	Baseline to 5 years after surgery
Non-cardiovascular death<br>*Any death not covered by above definition, such as death caused by infection, malignancy, sepsis, pulmonary causes, accident, suicide, or trauma*	Baseline to 5 years after surgery
**Concerning safety**	
Surgical re-exploration for bleeding/ tamponade<br>*Surgical incision into the sternum as a result of a bleeding or tamponade*<br>*a) acute: presented within 24 hours after surgery*<br>*b) late: presented after 24 hours after surgery*	Surgery to T4
Surgical re-exploration dehiscence<br>*Aseptic wound dehiscence*	Surgery to T4

[†]Specifically, any unexpected death even in patients with coexisting potentially fatal non-cardiac disease (e.g. cancer, infection) will be classified as cardiac death; [§]Not measured between the day of admission before surgery and 30 days after surgery, because of POST-in phase of Heart-ROCQ program; ICU: Intensive care unit; T4: Follow-up at 3 months after surgery; T6: Follow-up at 12 months after surgery.

For peer review only

Questionnaires and physical tests

Table 4 shows the physical tests and questionnaires of the secondary outcomes regarding physical and psychological health, life style risk factors, and the economic evaluation. Physical tests and questionnaires are completed at six assessment points (Figure 1). The preoperative measurements (T1 and T2) are conducted at the start (preoperative consultation) and the end (1 to 8 days before surgery) of the waiting period. The third measurement (T3) is performed when patients meet the UMCG discharge criteria (Table 2). The follow-up measurements (T4, T5, and T6) are at 3, 7, and 12 months after surgery. Patients are asked to fill in questionnaires online or when requested on paper, prior to the visits, to execute physical tests. All adverse events reported spontaneously by the patient or observed by the investigator are recorded. In addition, serious adverse events are reported to the METc. Data are stored in REDCap and on a secure drive at the department CardioResearch in the UMCG. Two times per year, the study is monitored by a trained research monitor from another department of the UMCG. Details of procedures, data collection, management and monitoring can be found in the Trial Master File of the Heart-ROCQ study (can be obtained by the investigators). On the 12th of June 2018, the study was audited and certified for the ISO 9001: 2015 independently by DNV GL.

<u>Potential moderators</u>

Preoperative risk profile (i.e. Euroscore II, medical history, life style risk factors, physical, and mental status), surgery parameters, and demographics are collected form the medical record and baseline measurements. The content of the CR program is described in terms of compliance, duration of CR program, type of treatment, frequency, and training load (i.e. for bicycle training: external workload, heart rate response, rate of perceived exertion using the Borg scale, and for strength training: sets, repetitions, and intensity) for both the Heart-ROCQ group as the Standard Care group.

For peer review only - http://bmjopen.bmj.com/site/about/guidelines.xhtml

Table 4: Time of measure, physical tests, and questionnaires of the secondary outcomes

Secondary outcomes	Measure	Time of measure
Physical health		
Cardiorespiratory fitness	6MWT(45)	T1-T4, T6
Muscle strength	STS_10, grip & leg strength(46,47)	T1-T4, T6
Independence in ADL	KATZ(48,49)	T1, T4, T6
Psychological health		
General anxiety	GAD-7(50)	T1,T2, T4, T6
Feelings of depression	PHQ-9(51,52)	T1,T2, T4, T6
Health related Quality of Life	Rand-36_v2(41)	T1, T4, T6
Life style risk factors		
Physical activity	iPAQ(53) & Actigraph†(54)	T1, T4, T6
Obesity indices	BMI, waist-to-hip ratio	T1, T4, T6
Smoking behaviour	Number of cigarettes per day	T1, T4, T6
Economic evaluation		
Health care use & related medical costs	iMCQ(55)	T1, T4-T6
Work participation‡	iPCQ(56)	T1, T4-T6
QALYs	EQ-5D-5L(57)	T1, T4-T6
Potential mediators		
Cardiac self-efficacy	CSE(58)	T1, T2, T4, T6
Illness representations	IPQ-R(59)	T1, T2, T4, T6

†Patients are asked to wear the Actigraph during waking hours for 1 consecutive week; ‡Health-related productivity losses of paid work and unpaid work; T1: Begin of waiting time; T2: 1 to 8 days before surgery; T3: Moment that patients meet the UMCG discharge criteria; T4-T6: Follow-up at 3, 7, and 12 months after surgery; 6MWT: Six minutes walking test; STS_10: Ten times sit to stand test; ADL: Activities of Daily Living; KATZ: Katz Index of Independence in Activities of Daily Living; GAD-7: Generalized Anxiety Disorder 7-item scale; PHQ-9: Patient Health Questionnaire 9-item scale; iPAQ: International Physical Activity Questionnaire; BMI: Body Mass Index; iMCQ: iMTA Medical Cost Questionnaire; iPCQ: iMTA Productivity Cost Questionnaire; QALY: Quality-adjusted life years; EQ-5D-5L: EuroQol five-dimensional questionnaire; CSE: Cardiac Self-efficacy; IPQ-R: Illness Perception Questionnaire, Revised.

Potential mediators

Illness perceptions (i.e. patients mental representations of their illness based on different sources of information) are measured with three subscales (personal control, treatment control, and consequences) of the revised illness perception questionnaire (IPQ-R).(59) These subscales are chosen because of their sensitivity to change and because of their relation to psychological distress(60). The cardiac self-efficacy (CSE) scale(58) is used to measure CSE (i.e. patients confidence to perform a specific task related to cardiac disease).

For peer review only - http://bmjopen.bmj.com/site/about/guidelines.xhtml

For peer review only

Statistical analyses

Sample size

Assuming a normal distribution, the mean weighted score of the primary end point is estimated on 1.0 with a standard deviation of 0.9 at 1 year after surgery. This estimation is based on historical data of the UMCG, an unpublished on-going pilot study in the UMCG, the Dutch registration database,(61) and data reported in literature.(6,62–64) A decrease of 0.3 is expected in the Heart-ROCQ group, based on previous studies comparing CR with standard care(36,63,65) (i.e. no CR) and is considered to be clinically relevant (i.e. on average a 30% decrease in complications/events or worsening in functional status or 10-20% decrease in MACE or death). To detect this decrease and achieve 80% power (significance level of 5%) a group of 286 patients (143 in the Heart-ROCQ group and 143 in the Standard Care group) is needed. To incorporate a withdrawal of ±20% a total sample size of 350 is needed at baseline.

Interim analysis

An interim analysis will be conducted when 40% of the included patients have had the measurements one year after surgery. The study will be terminated prematurely when the primary outcome of one of the CR programs is obviously (P<0.001) different from the other CR program.

Primary and sensitivity analyses

All end points are primary analysed according to the 'intention-to-treat' principles and missing values are counted as worst-case score (nominal variables) or estimated using Maximum Likelihood estimation (interval variables). As supplementary analyses, the end points are analysed on a per-protocol principle with and without using imputation methods for missing values. In all analyses, a two-sided p<0.05 is considered statistically significant.

Statistical methods and clinical relevance for analysing secondary outcomes are written in the research protocol (ClinicalTrials.gov : NCT02984449).

For peer review only - http://bmjopen.bmj.com/site/about/guidelines.xhtml

Economic evaluation

For the evaluation of health-care utilization, standard prices published in the Dutch costs guidelines are used.(66) To compare the costs to quality adjusted life years (QALYs), QALYs are estimated with use of the EQ-5D-5L.(57) Utility values for the EQ-5D-5L are calculated based on the new Dutch tariff.(67) Results from the analysis are reported as an Incremental Cost Effectiveness Ratio (ICER), dividing the difference in effect, by the difference in costs. Bootstrap re-sampling will be performed, and cost-effectiveness acceptability curves will be plotted, to estimate the probability that the Heart-ROCQ program is cost effective when compared to standard care. A societal perspective is applied.

Study status

From May 2017 to December 2018, 75 patients were enrolled. For next year's we expect that the enrolment will increase to 85 patients per year. The last patient is expected to be included in July 2021.

For peer review only

Discussion

The Heart-ROCQ study is the first randomised clinical trial evaluating the effect of a combination of pre- and post-operative CR program compared to a post-operative CR program. Furthermore, unlike the vast majority of CR programs in previous studies, the current program is multidisciplinary targeting different aspects of surgical outcomes in patients undergoing CS. Because different aspects are targeted, a composite weighted score will better reflect the treatment benefits than a single outcome. Therefore, the primary end point is a composite end point of functional status, post-operative surgical complications, readmissions to the hospital and MACE. The components of the end point are of clinical importance to patients undergoing CS and reflect a comprehensive representation of the recovery of the patient.

Both the ideas of the combined primary end point and the weighting of the individual components were derived from other studies.(40,68,69) Assigning different weights to the components was needed for more accurate comparison. Since the components are not equal in clinical importance, equal weights would lead to inaccurate statistical analysis(69). In the current study, the weighted score of HRQoL is lower (1 point instead of 2 points) to minimize bias due to patients' knowledge of group allocation. Therefore, improvements in quality of life are not counted in the primary end point to prevent bias in positive direction. This also prevents that a score in quality of life and event cancel each other out (e.g. when a patient experiences an improvement in Quality in Life [+2 points] and has a stroke [-2 points] then the total score is 0). The scores of all-cause mortality, stroke, myocardial infarction and revascularization are in line with the results showed by Tong and colleagues (2013)(69). A disadvantage of composite end points is that the effect may be driven by complications that occur with the greatest frequency.(70) Therefore, post-operative complications which occur frequently, such as atrial fibrillation, is evaluated separately as secondary end point. The primary end point is evaluated by an end-point committee, which is blinded to group allocation and consists of four independent cardiologists and cardio-thoracic surgeons.

Previous preoperative CR studies were primarily focused on short-term effects; only a few post-operative CR studies have determined long-term effects.(23,35,36) In contrast to previous

For peer review only - http://bmjopen.bmj.com/site/about/guidelines.xhtml

preoperative CR studies, the Heart-ROCQ study sets out to investigate both short- and long term effects of the CR programs.(23,35,36) Due to the trends in, among others, increasing age, obesity, and physical inactivity, patients undergoing CS are nowadays more complex. The Heart-ROCQ program aims to address these issues, which make the program clinically relevant for all CS patients. Therefore, we chose to include different types of CS. Since different moderators and mediators are assessed before, during and after CR, we can explore which factors are associated with better outcomes and which working mechanisms contribute to its effectiveness. These findings may provide a more in-depth understanding of who benefits the most from CR and the underlying mechanisms of CR, which are still not fully understood in patients undergoing CS on short and long-term.(35) In addition, the present study is thought to considerably attribute to the evidence to develop guidelines for clinical practice, especially regarding the preoperative CR program.

The Heart-ROCQ program is expected to be cost-effective in the long-term, which is also of interest for policymakers and health care takers. Therefore, an economic evaluation is performed to assess the cost-effectiveness of CR, since little is known about the cost-effectiveness of CR.(63) A societal perspective of this economic evaluation is chosen, meaning that not only health-care costs, but also patient- and productivity related costs and benefits are taken into account. If the Heart-ROCQ program is proven to be effective, it might be advisable to include a supportive prevention-oriented waiting period prior to surgery as an integral part of the treatment for patients undergoing elective CS. This implies a paradigm shift from curative care following CS to an additional preventive care attitude before surgery. Extensions of rehabilitation options in, or in the vicinity of cardiac centres, will then be required.

Conclusion

The Heart-ROCQ study is the first randomised clinical trial comparing the effect of a combined pre- and post-operative CR program to a regular Dutch phase II post-surgery outpatient rehabilitation CR program in a population undergoing elective CS. This study is expected to provide new understanding of the effectiveness and underlying working mechanisms of CR, and subsequently to improve value-based health care.

For peer review only - http://bmjopen.bmj.com/site/about/guidelines.xhtml

Acknowledgements

We thank E.J. Beens, M.J. Nijholt, Drs. A. Branderhorst, Dr. B. Dorhout (in memoriam), Dr. R. de Jong, H.A.M. Wasser, Dr. J. Brügemann, Dr. J.B. Wempe, Dr. J. Posma, Dr. C.A. Geluk, and Drs. M.L. Pentinga supporting the Heart-ROCQ study. In addition, we thank Drs. T.W. Waterbolk, Dr. R.G. Tieleman, and Dr. R.P.M. van Roosmalen participating in the end-point committee. We are grateful to all involved colleagues of the department of Cardiology, Cardio-thoracic Surgery, and Centre for Rehabilitation of the UMCG, OZG, MZH, and WZA for the pleasant collaboration and their help with executing the Heart-ROCQ study.

Declaration of interest

Drs. J. Hartog, Drs. S. Dijkstra, and Dr. M.A. Mariani report grants from Edwards Lifesciences SA, Abbott (former St. Jude Medical Nederland B.V.), and 'Stichting Beatrixoord Noord-Nederland' related to this study. In addition, Dr. M.A. Mariani reports consultancy from AtriCure, Getinge, and LivaNova. Dr. J. Fleer, Dr. W. Dieperink, Dr. L.H.V. van der Woude, Dr. M.F. Reneman, Drs. F. Blokzijl, Dr. I.C.C. van der Horst, Dr. M.J.L. DeJongste, and Dr. P. van der Harst have nothing to disclose.

Authors'contributions

Conception and design of the study: JH, FZ, MJLdJ, MFR, WD, JF, LHVvdW, PvdH, and MAM; Methodology: JH, FB, SD, MJLdJ, MFR, and PvdH; Acquisition of data: JH, FB and SD; Writing – Original draft, tables, and figures: JH; Writing- Reviewing and Editing: FB, SD, MJLdJ, MFR, WD, ICCvdH, JF, LHVvdW, PvdH, and MAM; Supervision: MJLdJ, MFR, WD, JF, ICCvdH, LHVvdW, PvdH, and MAM; Funding Acquisition: JH, SD, JF, LHVvdW, PvdH, and MAM. All authors approved the final version of the manuscript.

For peer review only

For peer review only - http://bmjopen.bmj.com/site/about/guidelines.xhtml

REFERENCES

1. GBD 2016 Causes of Death Collaborators. Global, regional, and national age-sex specific mortality for 264 causes of death, 1980–2016: a systematic analysis for the Global Burden of Disease Study 2016. Lancet. 2017;390(10100):1151–210.

2. Hulzebos EHJ, Van Meeteren NLU, De Bie RA, Dagnelie PC, Helders PJM. Prediction of postoperative pulmonary complications on the basis of preoperative risk factors in patients who had undergone coronary artery bypass graft surgery. Phys Ther. 2003;83(1):8–16.

3. Kassie GM, Nguyen TA, Kalisch Ellett LM, Pratt NL, Roughead EE. Preoperative medication use and postoperative delirium: a systematic review. BMC Geriatr. 2017 Dec 29;17(1):298.

4. Weymann A, Popov AF, Sabashnikov A, Ali-Hasan-Al-Saegh S, Ryazanov M, Tse G, et al. Baseline and postoperative levels of C-reactive protein and interleukins as inflammatory predictors of atrial fibrillation following cardiac surgery: a systematic review and meta-analysis. Kardiol Pol. 2018 Feb 16;76(2):440–51.

5. Koster S, Hensens AG, Schuurmans MJ, van der Palen J. Consequences of delirium after cardiac operations. Ann Thorac Surg. 2012 Mar;93(3):705–11.

6. Iribarne A, Chang H, Alexander JH, Gillinov AM, Moquete E, Puskas JD, et al. Readmissions after cardiac surgery: experience of the National Institutes of Health/Canadian Institutes of Health research cardiothoracic surgical trials network. Ann Thorac Surg. 2014 Oct;98(4):1274–80.

7. Villareal RP, Hariharan R, Liu BC, Kar B, Lee V-V, Elayda M, et al. Postoperative atrial fibrillation and mortality after coronary artery bypass surgery. J Am Coll Cardiol. 2004 Mar 3;43(5):742–8.

8. Hashemzadeh K, Dehdilani M, Dehdilani M. Postoperative Atrial Fibrillation following Open Cardiac Surgery: Predisposing Factors and Complications. J Cardiovasc Thorac Res. 2013;5(3):101–7.

9. Kosuma P, Wachirasrisirikul S, Jedsadayanmata A. Attributable Costs of Postoperative Atrial Fibrillation among Patients Undergoing Cardiac Surgery. Cardiol Res Pract. 2018;2018:1–5.

10. Findeisen A, Arefian H, Doenst T, Hagel S, Pletz MW, Hartmann M, et al. Economic burden of surgical site infections in patients undergoing cardiac surgery. Eur J Cardio-Thoracic Surg. 2018;0(8):1–7.

11. Ruiz-Núñez B, van den Hurk GHAM, de Vries JHM, Mariani MA, de Jongste MJL, Dijck-Brouwer DAJ, et al. Patients undergoing elective coronary artery bypass grafting exhibit poor pre-operative intakes of fruit, vegetables, dietary fibre, fish and vitamin D. Br J Nutr. 2015;(6):1–11.

12. Zittermann A, Kuhn J, Dreier J, Knabbe C, Gummert JF, Börgermann J. Vitamin D status and the risk of major adverse cardiac and cerebrovascular events in cardiac surgery. Eur Heart J. 2013;34(18):1358–64.

13. Kavanagh T, Mertens DJ, Hamm LF, Beyene J, Kennedy J, Corey P, et al. Prediction of long-term prognosis in 12 169 men referred for cardiac rehabilitation. Circulation. 2002;106(6):666–71.

14. van Laar C, Timman ST, Noyez L. Decreased physical activity is a predictor for a complicated recovery post cardiac surgery. Health Qual Life Outcomes. 2017;15(1):1–7.

15. Christian LM, Graham JE, Padgett DA, Glaser R, Kiecolt-Glaser JK. Stress and wound

For peer review only - http://bmjopen.bmj.com/site/about/guidelines.xhtml

healing. Neuroimmunomodulation. 2007;13(5–6):337–46.

16. Tully PJ, Baker RA. Depression, anxiety, and cardiac morbidity outcomes after coronary artery bypass surgery: a contemporary and practical review. J Geriatr Cardiol. 2012 Jun;9(2):197–208.

17. Kehler DS, Stammers AN, Tangri N, Hiebert B, Fransoo R, Schultz ASH, et al. Systematic review of preoperative physical activity and its impact on postcardiac surgical outcomes. BMJ Open. 2017;

18. Zhang YJ, Iqbal J, Van Klaveren D, Campos CM, Holmes DR, Kappetein AP, et al. Smoking is associated with adverse clinical outcomes in patients undergoing revascularization with PCI or CABG: The SYNTAX trial at 5-year follow-up. J Am Coll Cardiol. 2015;65(11):1107–15.

19. Mallik S, Krumholz HM, Zhen QL, Kasl S V., Mattera JA, Roumains SA, et al. Patients with depressive symptoms have lower health status benefits after coronary artery bypass surgery. Circulation. 2005;111(3):271–7.

20. Ghanta RK, Kaneko T, Gammie JS, Sheng S, Aranki SF. Evolving trends of reoperative coronary artery bypass grafting: An analysis of the Society of Thoracic Surgeons Adult Cardiac Surgery Database. J Thorac Cardiovasc Surg. 2013;145(2):364–72.

21. Kindo M, Hoang Minh T, Perrier S, Bentz J, Mommerot A, Billaud P, et al. Trends in isolated coronary artery bypass grafting over the last decade. Interact Cardiovasc Thorac Surg. 2017;24(1):71–6.

22. Carr T, Teucher U, Mann J, Casson AG. Waiting for surgery from the patient perspective. Psychol Res Behav Manag. 2009;2:107–19.

23. Arthur HM, Daniels C, McKelvie R, Hirsh J, Rush B. Effect of a preoperative intervention on preoperative and postoperative outcomes in low-risk patients awaiting elective coronary artery bypass graft surgery: A randomized, controlled trial. Ann Intern Med. 2000;133(4):253–62.

24. Van Der Peijl ID, Vliet Vlieland TPM, Versteegh MIM, Lok JJ, Munneke M, Dion RAE. Exercise therapy after coronary artery bypass graft surgery: A randomized comparison of a high and low frequency exercise therapy program. Ann Thorac Surg. 2004;77(5):1535–41.

25. Koopman C, Van Dis I, Vaartjes I, Visseren FLJ, Bots ML. Hart- en vaatziekten in Nederland 2014, cijfers over kwaliteit van leven, ziekte en sterfte. Harstichting. 2013. p. 192.

26. Takahashi T, Kumamaru M, Jenkins S, Saitoh M, Morisawa T, Matsuda H. In-patient step count predicts re-hospitalization after cardiac surgery. J Cardiol. 2015;66(4):286–91.

27. Cook DJ, Thompson JE, Prinsen SK, Dearani JA, Deschamps C. Functional recovery in the elderly after major surgery: Assessment of mobility recovery using wireless technology. Ann Thorac Surg. 2013;96(3):1057–61.

28. Kortebein P, Symons TB, Ferrando A, Paddon-Jones D, Ronsen O, Protas E, et al. Functional impact of 10 days of bed rest in healthy older adults. J Gerontol A Biol Sci Med Sci. 2008;63(10):1076–81.

29. Convertino V, Hung J, Goldwater D, DeBusk RF. Cardiovascular responses to exercise in middle-aged men after 10 days of bedrest. Circulation. 1982 Jan;65(1):134–40.

30. Chodzko-Zajko WJ, Proctor DN, Fiatarone Singh MA, Minson CT, Nigg CR, Salem GJ, et al. Exercise and physical activity for older adults. Med Sci Sports Exerc. 2009;41(7):1510–30.

31. BACPR. The British Association for Cardiovascular Prevention and Rehabilitation (BACPR)

For peer review only - http://bmjopen.bmj.com/site/about/guidelines.xhtml

standards and core components for cardiovascular disease prevention and rehabilitation, 2nd edition, 2012. 2012.

32. Bjarnason-Wehrens B, McGee H, Zwisler AD, Piepoli MF, Benzer W, Schmid J-P, et al. Cardiac rehabilitation in Europe: results from the European Cardiac Rehabilitation Inventory Survey. Eur J Cardiovasc Prev Rehabil. 2010;17(4):410–8.

33. De Vries H, Kemps HMC, Van Engen-Verheul MM, Kraaijenhagen RA, Peek N. Cardiac rehabilitation and survival in a large representative community cohort of Dutch patients. Eur Heart J. 2015;36:1519–28.

34. Anderson L, Taylor RS. Cardiac rehabilitation for people with heart disease: an overview of Cochrane systematic reviews. Cochrane database Syst Rev. 2014 Jan;12:CD011273.

35. Blokzijl F, Dieperink W, Keus F, Reneman MF, Mariani MA, van der Horst IC. Cardiac rehabilitation for patients having cardiac surgery: a systematic review. J Cardiovasc Surg (Torino). 2018 Apr 3;

36. Hulzebos EHJ, Smit Y, Helders PPJM, van Meeteren NLU. Preoperative physical therapy for elective cardiac surgery patients. Cochrane database Syst Rev. 2012 Jan;11:CD010118.

37. Vogelmeier CF, Criner GJ, Martinez FJ, Anzueto A, Barnes PJ, Bourbeau J, et al. Global Strategy for the Diagnosis, Management and Prevention of Chronic Obstructive Lung Disease 2017 Report. Respirology. 2017;22(3):575–601.

38. van Adrichem EJ, Meulenbroek RL, Plukker JTM, Groen H, van Weert E. Comparison of two preoperative inspiratory muscle training programs to prevent pulmonary complications in patients undergoing esophagectomy: a randomized controlled pilot study. Ann Surg Oncol. 2014 Jul;21(7):2353–60.

39. Van Dijk J. Multidisciplinaire Richtlijn Hartrevalidatie 2011. Tijdschr VOOR BEDRIJFS- EN Verzek. 2011;19(9):410–5.

40. Taylor AL, Ziesche S, Yancy C, Carson P, D'Agostino R, Ferdinand K, et al. Combination of Isosorbide Dinitrate an Hydralazine in Blacks with Heart Failure. N Engl J Med. 2004;351(20):2049–57.

41. Van der Zee KI, Sanderman R, Heyink JW, de Haes H, Van der Zee KI, Sanderman R, et al. Psychometric qualities of the RAND 36-Item Health Survey 1.0: a multidimensional measure of general health status. Int J Behav Med. 1996 Jan;3(2):104–22.

42. Wyrwich KW, Spertus JA, Kroenke K, Tierney WM, Babu AN, Wolinsky FD. Clinically important differences in health status for patients with heart disease: An expert consensus panel report. Am Heart J. 2004;147(4):615–22.

43. Thygesen K, Alpert JS, Jaffe AS, Simoons ML, Chaitman BR, White HD, et al. Third universal definition of myocardial infarction. Eur Heart J. 2012;33(20):2551–67.

44. Koster S, Hensens AG, Oosterveld FGJ, Wijma A, van der Palen J. The delirium observation screening scale recognizes delirium early after cardiac surgery. Eur J Cardiovasc Nurs. 2009;8(4):309–14.

45. Crapo RO, Casaburi R, Coates AL, Enright PL, MacIntyre NR, McKay RT, et al. ATS statement: Guidelines for the six-minute walk test. Am J Respir Crit Care Med. 2002;166(1):111–7.

46. Puthoff ML, Saskowski D. Reliability and responsiveness of gait speed, five times sit to stand, and hand grip strength for patients in cardiac rehabilitation. Cardiopulm Phys Ther J. 2013

For peer review only

For peer review only - http://bmjopen.bmj.com/site/about/guidelines.xhtml

Mar;24(1):31–7.

47. Douma KW, Regterschot GRH, Krijnen WP, Slager GEC, van der Schans CP, Zijlstra W. Reliability of the Q Force; a mobile instrument for measuring isometric quadriceps muscle strength. BMC Sports Sci Med Rehabil. 2016 Jan;8:4.

48. Katz S, Ford AB, Moskowitz RW, Jackson BA, Jaffe MW, Cleveland MA. Studies of Illness in the Aged. The Index of ADL: A Standardized Measure of Biological and Psychosocial Function. JAMA. 1963;185(12):914–9.

49. Reijneveld SA, Spijker J, Dijkshoorn H. Katz' ADL index assessed functional performance of Turkish, Moroccan, and Dutch elderly. J Clin Epidemiol. 2007;60(4):382–8.

50. Spitzer RL, Kroenke K, Williams JBW, Löwe B. A Brief Measure for Assessing Generalized Anxiety Disorder: the GAD-7. Arch Intern Med. 2006;166(10):1092–7.

51. Kroenke K, Spitzer RL, Williams JB. The PHQ-9: validity of a brief depression severity measure. J Gen Intern Med. 2001 Sep;16(9):606–13.

52. Löwe B, Unützer J, Callahan CM, Perkins AJ, Kroenke K. Monitoring depression treatment outcomes with the patient health questionnaire-9. Med Care. 2004 Dec;42(12):1194–201.

53. Craig CL, Marshall AL, Sjöström M, Bauman AE, Booth ML, Ainsworth BE, et al. International physical activity questionnaire: 12-Country reliability and validity. Med Sci Sports Exerc. 2003;35(8):1381–95.

54. Whitaker KM, Gabriel KP, Jacobs DR, Sidney S, Sternfeld B. Comparison of Two Generations of ActiGraph Accelerometers: The CARDIA Study. Med Sci Sport Exerc. 2018;50(6):1333–40.

55. Bouwmans C, Hakkaart-van Roijen L, Koopmanschap M, Krol M, Severens H BW. Medical Cost Questionnaire (iMCQ). Rotterdam: iMTA, Erasmus Universiteit Rotterdam. 2013 [cited 2016 May 1]. Available from: https://www.imta.nl/

56. Bouwmans C, Krol M, Severens H, Koopmanschap M, Brouwer W, Roijen LH Van. The iMTA Productivity Cost Questionnaire: A Standardized Instrument for Measuring and Valuing Health-Related Productivity Losses. Value Heal. 2015;18(6):753–8. Available from: http://dx.doi.org/10.1016/j.jval.2015.05.009

57. EuroQol Group. What is EQ-5D? 2014 [cited 2016 Nov 3]. Available from: http://www.euroqol.org/

58. Sullivan MD, LaCroix AZ, Russo J, Katon WJ. Self-efficacy and self-reported functional status in coronary heart disease: a six-month prospective study. Psychosom Med. 1998;60(4):473–8.

59. Moss-Morris R, Weinman J, Petrie K. The revised illness perception questionnaire (IPQ-R). Psychol Heal. 2002;17(1):1–16.

60. Morgan K, Villiers-Tuthill A, Barker M, McGee H. The contribution of illness perception to psychological distress in heart failure patients. BMC Psychol. 2014 Nov 28;2(1):50.

61. Meetbaar Beter Boek 2015 [cited 2016 Apr 25]. p. 60. Available from: http://www.meetbaarbeter.com/documents/meetbaar-beter-boeken/

62. Engblom E, Korpilahti K, Hämäläinen H, Rönnemaa T, Puukka P. Quality of life and return to work 5 years after coronary artery bypass surgery. Long-term results of cardiac rehabilitation. J Cardiopulm Rehabil. 1997 Jan;17(1):29–36.

63. Heran BS, Chen JM, Ebrahim S, Moxham T, Oldridge N, Rees K, et al. Exercise-based

For peer review only

For peer review only - http://bmjopen.bmj.com/site/about/guidelines.xhtml

cardiac rehabilitation for coronary heart disease. Cochrane database Syst Rev. 2011 Jul 6;(7):CD001800.

64. Serruys PW, Morice M, Kappetein AP, Colombo A, Holmes DR, Mack MJ, et al. Percutaneous Coronary Intervention versus Coronary-Artery Bypass Grafting for Severe Coronary Artery Disease. N Engl J Med. 2009;360:961–72.

65. Jolliffe JA, Rees K, Taylor RS, Thompson D, Oldridge N, Ebrahim S. Exercise-based rehabilitation for coronary heart disease. Cochrane database Syst Rev. 2001 Jan;(1):CD001800.

66. Zorginstituut Nederland. Kostenhandleiding: Methodologie van kostenonderzoek en referentieprijzen voor economische evaluaties in de gezondheidszorg. 2015.

67. Versteegh MM, Vermeulen KM, Evers SMAA, de Wit GA, Prenger R, Stolk EA. Dutch Tariff for the Five-Level Version of EQ-5D. Value Health. 2016 Jun;19(4):343–52.

68. de Vries AE, de Jong RM, van der Wal MH, Jaarsma T, van Dijk RB, Hillege HL. The value of INnovative ICT guided disease management combined with Telemonitoring in OUtpatient clinics for Chronic Heart failure patients. Design and methodology of the IN TOUCH study: a multicenter randomised trial. BMC Health Serv Res. 2011;11(1):167.

69. Tong BC, Huber JC, Ascheim DD, Puskas JD, Ferguson Jr B, Blackstone EH, et al. Weighting Composite Endpoints in Clinical Trials: Evidence for the Heart Team. 2013;94(6):1908–13.

70. Anker SD, Schroeder S, Atar D, Bax JJ, Ceconi C, Cowie MR, et al. Traditional and new composite endpoints in heart failure clinical trials: facilitating comprehensive efficacy assessments and improving trial efficiency. Eur J Heart Fail. 2016 May;18(5):482–9.

For peer review only

For peer review only - http://bmjopen.bmj.com/site/about/guidelines.xhtml

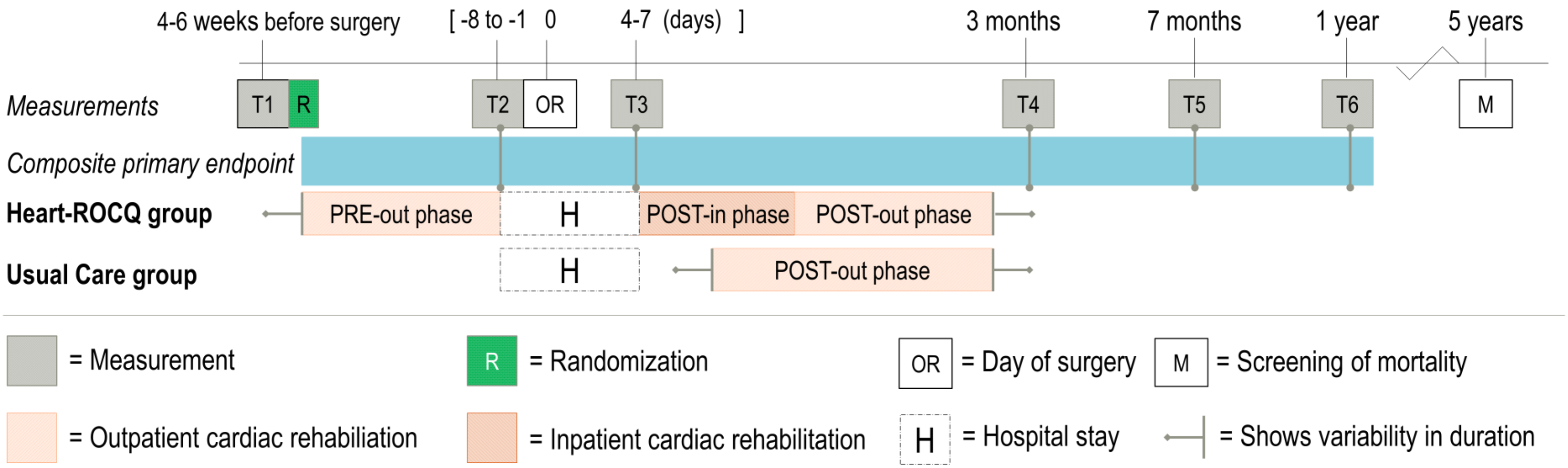

Figure 1: Research design of the Heart-ROCQ PROBE trial. The phases of both cardiac rehabilitation programs and the measurements are shown relative to the moment of surgery.

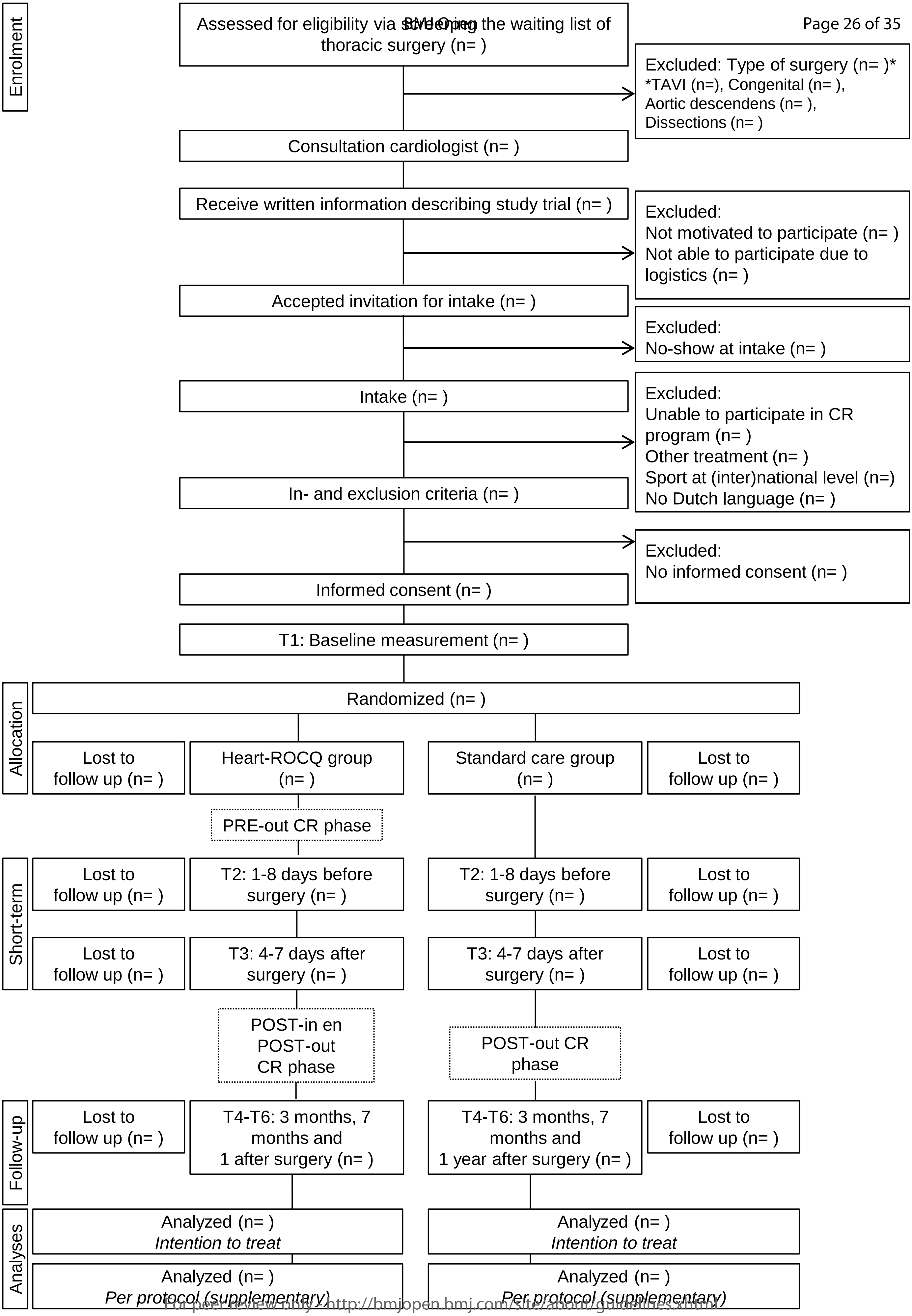
Enrolment
Assessed for eligibility via screening the waiting list of thoracic surgery (n=)
Excluded: Type of surgery (n=)*
*TAVI (n=), Congenital (n=), Aortic descendens (n=), Dissections (n=)
Consultation cardiologist (n=)
Receive written information describing study trial (n=)
Excluded:
Not motivated to participate (n=)
Not able to participate due to logistics (n=)
Accepted invitation for intake (n=)
Excluded:
No-show at intake (n=)
Intake (n=)
Excluded:
Unable to participate in CR program (n=)
Other treatment (n=)
Sport at (inter)national level (n=)
No Dutch language (n=)
In- and exclusion criteria (n=)
Excluded:
No informed consent (n=)
Informed consent (n=)
T1: Baseline measurement (n=)
Allocation
Randomized (n=)
Lost to follow up (n=)
Heart-ROCQ group (n=)
Standard care group (n=)
Lost to follow up (n=)
PRE-out CR phase
Short-term
Lost to follow up (n=)
T2: 1-8 days before surgery (n=)
T2: 1-8 days before surgery (n=)
Lost to follow up (n=)
Lost to follow up (n=)
T3: 4-7 days after surgery (n=)
T3: 4-7 days after surgery (n=)
Lost to follow up (n=)
POST-in en POST-out CR phase
POST-out CR phase
Follow-up
Lost to follow up (n=)
T4-T6: 3 months, 7 months and 1 after surgery (n=)
T4-T6: 3 months, 7 months and 1 year after surgery (n=)
Lost to follow up (n=)
Analyses
Analyzed (n=)
Intention to treat
Analyzed (n=)
Intention to treat
Analyzed (n=)
Per protocol (supplementary)
Analyzed (n=)
Per protocol (supplementary)

SUPPLEMENTARY INFORMATION - DESCRIPTION OF THE HEART-ROCQ PROGRAM

PRE-out phase – An outpatient cardiac rehabilitation phase during the waiting period
Three times per week, a minimum of three weeks

Physical therapy

Aims
- To maintain or improve patients physical capacity before surgery
- Patient learns to apply the stress-strain training principles
- To optimize pulmonary muscle strength
- Patient knows which breathing and coughing techniques should be used after surgery to prevent pulmonary complications

When needed:
- Patient learns to recognize body signals and boundaries.
- Patient is able to exercise despite of possible kinesiophobia

Type of exercise	Frequency	Intensity	Monitoring
IMT	3 × p / wk	- 6 cycles of 6 repetitions,<br>- rest periods of resp. 60, 45, 30, 15 and 5 s.<br>- 60-80% of maximal inspiratory pressure[65]	- Week 1: each training ↑ intensity with 10%<br>- Intensity ↑ with 5% if RPE <5[1]
Aerobic cycle	3 × p / wk	- 25 min. at RPE 3[1]<br>- Interval training will be given, if the patient is not able to perform endurance training.	- First training: 50% of POpeak<br>- Load ↑ if RPE< 3[1]<br>- Interval training: guided by complaints and RPE of 3
Resistance training	3 × p / wk	- 1-3 cycles of 10-15 repetitions<br>- Rest: 30-60 s.<br>- 50-80% of estimated 1RM<br>- On six fitness apparatus	- First training: 6-10 RM per fitness apparatus
Body awareness	1 × p / 2 wks	- 30 min. breathing and relaxation techniques	Not Applicable
Group education	Two sessions	- Basic training principles<br>- Forced expiration, huff- and cough techniques	Not Applicable

Dietary advice[2]

Aims
- To maintain or improve patients nutritional status
- Patient knows which nutrients are important focusing on the surgery
- Patients knows the importance of food in relation to cardiovascular disease and cardiovascular risk management

Type	Frequency	Content
Intake interview	One session	- Anamnesis about nutritional status
Counselling	On indication	- Individual sessions according to existing guidelines
Group education	One session	- 60 min., cardiovascular risk factors and dietary intake

Psychological guidance[2]

Aims
- To optimize mental status of the patients before surgery
- Patient has made a start with the awareness of cardiovascular risk factors

Intake interview	One session	- Anamnesis about mental status
Counselling	On indication	- Individual sessions according to evidence-based treatment protocols dependent on mental problems of patient[3]
Group education	One session	- Coping with psychological tension/stress, to process the mental trauma of cardiac surgery, and risk factors

No-smoking consultation *(For patients who smoke)*

Aim
- Patient gives up smoking during the waiting time before surgery

Intake interview & counselling	One session per wk	- 30 min., individual session based on existing guidelines

IMT: Inspiratory muscle training; RPE: Rate perceives exertion; POpeak: Maximum power output achieved during submaximal Ergometry test. [1]On a Borgscale 0-10; [2]Involvement of partner/relatives during group and individual sessions; [3] like cognitive behavioural therapy, eye movement desensitization and reprocessing (EMDR), acceptance and commitment therapy etc.

For peer review only - http://bmjopen.bmj.com/site/about/guidelines.xhtml

POST-in phase – An inpatient cardiac rehabilitation phase
Starting 4-7 days after surgery, duration of three weeks, weekends at home

Physical therapy

Aim
- To recover patients physical capacity
- Patient performs breathing and coughing techniques to prevent pulmonary complications
- Patient mobilize and can perform activities of daily living independently
- Patient knows about risk factors according to physical activity, knows the ACSM recommendations (moderate-intensity cardiorespiratory exercise training for ≥30min/day ≥5 days/week or vigorous-intensity cardiorespiratory exercise training for ≥20 min/day on ≥3 days/week or a combination[66]) and makes a plan to apply the ACSM recommendations in his own life
- Patient works on personal goals

Type of exercise	Frequency	Intensity	Monitoring
Individual therapy	2 × p / day	- Practice at transfers, walking, and climbing stairs<br>- Very light mobilizing exercises for upper extremity	- During the first 2 days of this phase.<br>- Extended, if patient is not able to participate in the group sessions.
Individual therapy	2 × p / wk	- Attention to personal goals	- Week 2 and 3
IMT[2]	3 × p / wk: 2 × under supervision, 1 × without supervision	- 6 cycles of 6 repetitions,<br>- rest periods of resp. 60, 45, 30, 15 and 5 s.<br>- intensity of 60-80% of maximum inspiratory pressure[65]	- First training: 50% of resistance of last preoperative training<br>- Intensity ↑ with 5% if RPE <5[1]<br>- IMT stops when resistance of preoperative training is reached
Aerobic cycle[2]	1 × p / day	- Week 1: duration of 5-20 min. at light intensity (RPE 2[1])<br>- Week 2 and 3: Work up to 25 min. at moderate intensity (RPE 3[1])<br>- Interval training will be given, if the patient is not able to perform endurance training.	- First training: at 50% of power output of last preoperative training<br>- Load ↑ if RPE< 2 à 3[1]<br>- Interval training: guided by complaints and RPE of 3.
Resistance training[2]	3-4 × p / wk	- 3 cycles of 15-20 repetitions<br>- Rest periods of 30-60 seconds<br>- On 6 fitness apparatus	- First training: 50% of resistance of last preoperative training for LE and 25% for UE.<br>- Gradual build up to 50-80% van 1RM based on RPE 3[1]
Body awareness[2]	1 × p / wk	- 30 min. breathing and relaxation techniques	Not Applicable

Dietary advice[3]

Aim
- To maintain or improve patients nutritional status
- Patient knows which nutrients are important focusing on the recovery of surgery
- Patients knows the importance of food in relation to cardiovascular disease and cardiovascular risk management

Type	Frequency	Content
Intake interview	One session	- Anamnesis about nutritional status post-surgery
Counselling	Sessions on indication	- Treatment according to existing guidelines
Group education	One session	- 90 min., skills needed to maintain a healthy life style

Psychological guidance[3]

Aim
- Patient start to process the mental trauma of cardiac surgery and the consequences of it
- Patient has self-management competence to maintain a healthy life style
- Patient and partner/relatives are able to support each other in the processing process

Intake interview	One session	- Anamnesis about mental status post-surgery
Intake interview & counselling	Sessions on indication	- Individual sessions according to evidence-based treatment protocols dependent on mental problems of patient[4]
Group education	One session	- 90 min., processing the cardiac surgery: possible reactions en consequences, coping with stress and healthy life style

IMT: Inspiratory muscle training; RPE: Rate perceives exertion; LE: Lower extremities; UE: Upper extremities. [1]On a Borgscale 0-10; [2]Group sessions start from day three of the inpatient phase or when patient is able to perform in the group activities; [3]Involvement of partner/relatives during group and individual sessions; [4] like cognitive behavioural therapy, eye movement desensitization and reprocessing (EMDR), acceptance and commitment therapy etc.

For peer review only - http://bmjopen.bmj.com/site/about/guidelines.xhtml

**POST-in phase – An inpatient cardiac rehabilitation phase (continuation)**<br>*Starting 4-7 days after surgery, duration of three weeks, weekends at home*		
**No-smoking consultation**[1] *(For patients who smoke)*		
Aim<br>- Patient is still motivated to stop smoking or patient is motivated to give up smoking (when patient did not give up smoking before cardiac surgery.		
Intake interview & counseling	1 × p / wk	- 30 min., individual sessions based on
Group education	One session	- 60 min., general information about smoking addiction and support from fellow smokers
**Return to work consultation**[1] *(for patients who are employed)*		
Aim<br>- Patient is informed about laws and regulation for illness, social security contributions, and medical examinations<br>- Patient knows their rights and obligations, the rights and obligations of the employer, the working conditions agency, the employee insurance agency, and re-integration companies<br>- Patient received tools to return adequately back to work (knows positive and negative factors that can impact the re-integration)		
Group education	One session	- 60 min., laws and regulation for illness, procedures, roles, rights and obligations of different involved persons, communication to involved persons (e.g. colleagues, employer), working during rehabilitation, positive and negative factors regarding to return to work
Counselling	Sessions on indication	- Individual sessions with labour consultant dependent on problems of patient

[1]Involvement of partner/relatives during group and individual sessions.

For peer review only

For peer review only - http://bmjopen.bmj.com/site/about/guidelines.xhtml

POST-out phase – An outpatient cardiac rehabilitation phase
Starting on Tuesday after discharge of the POST-in phase, two times per week, four weeks

Physical therapy

Aim

- Patient has optimized his/her physical capacity
- Patient knows his/her boundaries and limitations
- Patient knows about cardiovascular risk factors according to physical activity, knows the ACSM recommendations (moderate-intensity cardiorespiratory exercise training for ≥30min/day ≥5 days/week or vigorous-intensity cardiorespiratory exercise training for ≥20 min/day on ≥3 days/week or a combination[65]) and makes a plan to apply the ACSM recommendations in his own life
- Patient resumes his/her work or hobbies
- Patient experiences pleasure during exercise
- Patients achieves their personal goals

**Type of exercise**	**Frequency**	**Intensity**	**Monitoring**
Aerobic cycle	2 × p / wk	Depending on trainability of patient<br>- 25 min. at 50-80% HRR or RPE 5[1]	- Work up to 25 minutes at moderate intensity (RPE 3[1]), when patient was not able to do it in POST-in phase<br>- Load ↑ if RPE< 5[1] or HRR <50-80%
Resistance training	2 × p / wk	UE:<br>- 3-4 cycles of 10-15 repetitions<br>- Rest: 30-60 s.<br>- 50-80% of estimated 1RM<br>- On six fitness apparatus<br>LE:<br>- Focus on mobilization in week 1 & 2<br>- Focus on strength and endurance in week 3 & 4	- UE: Load ↑ if RPE< 5[1]<br>- LE: Under guidance of complaints
Sport and games	1 × p / wk	Focus on:<br>- Experiencing pleasure during exercise, regaining trust and handling boundaries.<br>- Exploring different types of sports and knowing the possibilities after CR	
Swimming	1 × p / wk		
Education	1 × p / wk	- Training principles of POST-out phase and a repetition of ACSM recommendations<br>- Awareness of exercise after CR; making a plan to exercise after CR<br>- Explanation of the results of the exercise test<br>- Discussing the plan to exercise, share experiences to expand exercise in home situation	

Dietary advice, psychological guidance, no-smoking consultation, and return to work consultation

Individual sessions are continued when aims are not achieved

RPE: Rate perceives exertion; HRR: Heart rate reserve; LE: Lower extremities; UE: Upper extremities; ACSM: American College of Sports Medicine; CR: Cardiac Rehabilitation. [1]On a Borgscale 0-10; [2]Involvement of partner/relatives during group and individual sessions.

For peer review only

For peer review only - http://bmjopen.bmj.com/site/about/guidelines.xhtml

SPIRIT

STANDARD PROTOCOL ITEMS: RECOMMENDATIONS FOR INTERVENTIONAL TRIALS

SPIRIT 2013 Checklist: Recommended items to address in a clinical trial protocol and related documents*

Section/item	Item No	Description	Addressed on page number
**Administrative information**			
Title	1	Descriptive title identifying the study design, population, interventions, and, if applicable, trial acronym	______1_______
Trial registration	2a	Trial identifier and registry name. If not yet registered, name of intended registry	______3, 6_____
	2b	All items from the World Health Organization Trial Registration Data Set	______3, 6_____
Protocol version	3	Date and version identifier	________6______
Funding	4	Sources and types of financial, material, and other support	______7_______
Roles and responsibilities	5a	Names, affiliations, and roles of protocol contributors	______1, 19___
	5b	Name and contact information for the trial sponsor	______1_______
	5c	Role of study sponsor and funders, if any, in study design; collection, management, analysis, and interpretation of data; writing of the report; and the decision to submit the report for publication, including whether they will have ultimate authority over any of these activities	______7_______
	5d	Composition, roles, and responsibilities of the coordinating centre, steering committee, endpoint adjudication committee, data management team, and other individuals or groups overseeing the trial, if applicable (see Item 21a for data monitoring committee)	______7, 8, 10, 13

For peer review only

Introduction

Background and rationale	6a	Description of research question and justification for undertaking the trial, including summary of relevant studies (published and unpublished) examining benefits and harms for each intervention	___4, 5______
	6b	Explanation for choice of comparators	___4, 5_______
Objectives	7	Specific objectives or hypotheses	___5_________
Trial design	8	Description of trial design including type of trial (eg, parallel group, crossover, factorial, single group), allocation ratio, and framework (eg, superiority, equivalence, noninferiority, exploratory)	___6, 7, ______

Methods: Participants, interventions, and outcomes

Study setting	9	Description of study settings (eg, community clinic, academic hospital) and list of countries where data will be collected. Reference to where list of study sites can be obtained	___6________
Eligibility criteria	10	Inclusion and exclusion criteria for participants. If applicable, eligibility criteria for study centres and individuals who will perform the interventions (eg, surgeons, psychotherapists)	___7________
Interventions	11a	Interventions for each group with sufficient detail to allow replication, including how and when they will be administered	___9________
	11b	Criteria for discontinuing or modifying allocated interventions for a given trial participant (eg, drug dose change in response to harms, participant request, or improving/worsening disease)	___NA______
	11c	Strategies to improve adherence to intervention protocols, and any procedures for monitoring adherence (eg, drug tablet return, laboratory tests)	___13______
	11d	Relevant concomitant care and interventions that are permitted or prohibited during the trial	___7_______
Outcomes	12	Primary, secondary, and other outcomes, including the specific measurement variable (eg, systolic blood pressure), analysis metric (eg, change from baseline, final value, time to event), method of aggregation (eg, median, proportion), and time point for each outcome. Explanation of the clinical relevance of chosen efficacy and harm outcomes is strongly recommended	___10-14___
Participant timeline	13	Time schedule of enrolment, interventions (including any run-ins and washouts), assessments, and visits for participants. A schematic diagram is highly recommended (see Figure)	____6-14___

For peer review only - http://bmjopen.bmj.com/site/about/guidelines.xhtml

Sample size	14	Estimated number of participants needed to achieve study objectives and how it was determined, including clinical and statistical assumptions supporting any sample size calculations	___15_______
Recruitment	15	Strategies for achieving adequate participant enrolment to reach target sample size	___7, 8, 16___

Methods: Assignment of interventions (for controlled trials)

Allocation:

Sequence generation	16a	Method of generating the allocation sequence (eg, computer-generated random numbers), and list of any factors for stratification. To reduce predictability of a random sequence, details of any planned restriction (eg, blocking) should be provided in a separate document that is unavailable to those who enrol participants or assign interventions	___7, 8______
Allocation concealment mechanism	16b	Mechanism of implementing the allocation sequence (eg, central telephone; sequentially numbered, opaque, sealed envelopes), describing any steps to conceal the sequence until interventions are assigned	___7, 8______
Implementation	16c	Who will generate the allocation sequence, who will enrol participants, and who will assign participants to interventions	____7, 8_____
Blinding (masking)	17a	Who will be blinded after assignment to interventions (eg, trial participants, care providers, outcome assessors, data analysts), and how	____7, 8_____
	17b	If blinded, circumstances under which unblinding is permissible, and procedure for revealing a participant's allocated intervention during the trial	___NA________

Methods: Data collection, management, and analysis

Data collection methods	18a	Plans for assessment and collection of outcome, baseline, and other trial data, including any related processes to promote data quality (eg, duplicate measurements, training of assessors) and a description of study instruments (eg, questionnaires, laboratory tests) along with their reliability and validity, if known. Reference to where data collection forms can be found, if not in the protocol	__11-14_______
	18b	Plans to promote participant retention and complete follow-up, including list of any outcome data to be collected for participants who discontinue or deviate from intervention protocols	__NA_________

Data management	19	Plans for data entry, coding, security, and storage, including any related processes to promote data quality (eg, double data entry; range checks for data values). Reference to where details of data management procedures can be found, if not in the protocol	__13_________
Statistical methods	20a	Statistical methods for analysing primary and secondary outcomes. Reference to where other details of the statistical analysis plan can be found, if not in the protocol	___15, 16_____
	20b	Methods for any additional analyses (eg, subgroup and adjusted analyses)	___15________
	20c	Definition of analysis population relating to protocol non-adherence (eg, as randomised analysis), and any statistical methods to handle missing data (eg, multiple imputation)	_____15_______
**Methods: Monitoring**			
Data monitoring	21a	Composition of data monitoring committee (DMC); summary of its role and reporting structure; statement of whether it is independent from the sponsor and competing interests; and reference to where further details about its charter can be found, if not in the protocol. Alternatively, an explanation of why a DMC is not needed	___13________
	21b	Description of any interim analyses and stopping guidelines, including who will have access to these interim results and make the final decision to terminate the trial	___15________
Harms	22	Plans for collecting, assessing, reporting, and managing solicited and spontaneously reported adverse events and other unintended effects of trial interventions or trial conduct	___13________
Auditing	23	Frequency and procedures for auditing trial conduct, if any, and whether the process will be independent from investigators and the sponsor	___13________
**Ethics and dissemination**			
Research ethics approval	24	Plans for seeking research ethics committee/institutional review board (REC/IRB) approval	___6________
Protocol amendments	25	Plans for communicating important protocol modifications (eg, changes to eligibility criteria, outcomes, analyses) to relevant parties (eg, investigators, REC/IRBs, trial participants, trial registries, journals, regulators)	___6_________

Consent or assent	26a	Who will obtain informed consent or assent from potential trial participants or authorised surrogates, and how (see Item 32)	__7, 8_______
	26b	Additional consent provisions for collection and use of participant data and biological specimens in ancillary studies, if applicable	__7, 8_______
Confidentiality	27	How personal information about potential and enrolled participants will be collected, shared, and maintained in order to protect confidentiality before, during, and after the trial	__13_________
Declaration of interests	28	Financial and other competing interests for principal investigators for the overall trial and each study site	__19_________
Access to data	29	Statement of who will have access to the final trial dataset, and disclosure of contractual agreements that limit such access for investigators	__13_________
Ancillary and post-trial care	30	Provisions, if any, for ancillary and post-trial care, and for compensation to those who suffer harm from trial participation	__13_________
Dissemination policy	31a	Plans for investigators and sponsor to communicate trial results to participants, healthcare professionals, the public, and other relevant groups (eg, via publication, reporting in results databases, or other data sharing arrangements), including any publication restrictions	___6_________
	31b	Authorship eligibility guidelines and any intended use of professional writers	___6_________
	31c	Plans, if any, for granting public access to the full protocol, participant-level dataset, and statistical code	___NA_______
**Appendices**			
Informed consent materials	32	Model consent form and other related documentation given to participants and authorised surrogates	___13________
Biological specimens	33	Plans for collection, laboratory evaluation, and storage of biological specimens for genetic or molecular analysis in the current trial and for future use in ancillary studies, if applicable	___NA________

*It is strongly recommended that this checklist be read in conjunction with the SPIRIT 2013 Explanation & Elaboration for important clarification on the items. Amendments to the protocol should be tracked and dated. The SPIRIT checklist is copyrighted by the SPIRIT Group under the Creative Commons “Attribution-NonCommercial-NoDerivs 3.0 Unported” license.

For peer review only - http://bmjopen.bmj.com/site/about/guidelines.xhtml

HEART Rehabilitation in patients awaiting Open-heart surgery targeting to prevent Complications and to improve Quality of life (Heart-ROCQ): Study protocol for a Prospective Randomised Open Blinded End-point (PROBE) trial

Journal:	*BMJ Open*
Manuscript ID	bmjopen-2019-031738.R1
Article Type:	Protocol
Date Submitted by the Author:	05-Jul-2019
Complete List of Authors:	Hartog, Johanneke; UMCG, Cardio-thoracic Surgery<br>Blokzijl, Fredrike; UMCG, Cardio-thoracic Surgery<br>Dijkstra, Sandra; UMCG, Cardio-thoracic Surgery<br>DeJongste, Mike; UMCG<br>Reneman, Michiel; UMCG, Rehabilitation Medicine<br>Dieperink, Willem; UMCG, Critical Care<br>van der Horst, Iwan; UMCG, Critical Care<br>Fleer, Joke; UMCG, Health Psychology<br>van der Woude, Lucas; UMCG, Center for Human Movement Sciences; UMCG, Rehabilitation Medicine<br>Van der Harst, Pim; UMCG, Cardiology<br>Mariani, Massimo; UMCG, Cardio-thoracic surgery
**Primary Subject Heading**:	Cardiovascular medicine
Secondary Subject Heading:	Cardiovascular medicine, Health economics, Mental health, Rehabilitation medicine, Surgery
Keywords:	rehabilitation, cardiac surgical procedures, life style, health status, postoperative complications, cost-benefit analysis

SCHOLARONE™ Manuscripts

Trial design BMJ Open

Title

HEART Rehabilitation in patients awaiting **O**pen-heart surgery targeting to prevent **C**omplications and to improve **Q**uality of life (Heart-ROCQ) - Study protocol for a **P**rospective **R**andomised **O**pen **B**linded **E**nd-point (PROBE) trial

Authors

Johanneke Hartog, MSc[a]; Fredrike Blokzijl, MSc[a]; Sandra Dijkstra, MSc[a]; Mike J.L. DeJongste, M.D., PhD, FESC[b]; Michiel F. Reneman, PhD[c]; Willem Dieperink, PhD[d]; Iwan C.C. van der Horst, M.D., PhD[d]; Joke Fleer, PhD[e]; Lucas H.V. van der Woude, PhD[c,f]; Pim van der Harst, M.D., PhD[g]; Massimo A. Mariani, M.D., PhD[a]

a University of Groningen, University Medical Centre Groningen, Department of Cardio-thoracic Surgery, Groningen, The Netherlands

b University of Groningen, University Medical Centre Groningen, Groningen, The Netherlands

c University of Groningen, University Medical Centre Groningen, Department of Rehabilitation Medicine, Groningen, The Netherlands

d University of Groningen, University Medical Centre Groningen, Department of Critical Care, Groningen, The Netherlands

e University of Groningen, University Medical Centre Groningen, Department of Health Psychology, Groningen, The Netherlands

f University of Groningen, University Medical Centre Groningen, Centre for Human Movement Sciences, Groningen, The Netherlands

g University of Groningen, University Medical Centre Groningen, Department of Cardiology, Groningen, The Netherlands

Corresponding Author:

Johanneke Hartog, Department of Cardiothoracic Surgery, University Medical Centre Groningen (UMCG), Hanzeplein 1, AB41, 9713 GZ Groningen, The Netherlands

Phone: +3150 3617527; Email: j.hartog@umcg.nl; FAX: +31503614391

For peer review only

Abstract

Introduction

The rising prevalence of modifiable risk factors (e.g. obesity, hypertension, and physical inactivity) is causing an increase in possible avoidable complications in patients undergoing cardiac surgery. This study aims to assess whether a combined pre- and postoperative multidisciplinary cardiac rehabilitation (CR) program (Heart-ROCQ program) can improve functional status and reduce surgical complications, readmissions and major adverse cardiac events (MACE) as compared to standard care.

Methods and analysis

Patients (n=350) are randomised to the Heart-ROCQ program or standard care. The Heart-ROCQ program consists of a preoperative optimization phase whilst waiting for surgery (3 times p/week, minimum 3 weeks), a post-operative inpatient phase (3 weeks) and an outpatient CR phase (2 times p/week, 4 weeks). Patients receive multidisciplinary treatment (e.g. physical therapy, dietary advice, psychological sessions, and smoking cessation). Standard care consists of 6 weeks post-surgery outpatient CR with education and physical therapy (2 times p/week). The primary outcome is a composite weighted score of functional status, surgical complications, readmissions, and MACE and is evaluated by a blinded end-point committee. Secondary outcomes are length of stay, physical, and psychological functioning, life style risk factors, and work participation. Finally, an economic evaluation is performed. Data is collected at six time points: at baseline (start of the waiting period), the day before surgery, at discharge from the hospital, and at 3, 7, and 12 months post-surgery.

Ethics and dissemination

This study will be conducted according to the principles of the Declaration of Helsinki (version 8, October 2013). The protocol has been approved by the Medical Ethical Review Board (METc) of the UMCG (No. 2016/464). Results of this study will be submitted to a peer-reviewed scientific journal and can be presented at national and international conferences.

For peer review only - http://bmjopen.bmj.com/site/about/guidelines.xhtml

Registration: The Heart-ROCQ study has been registered at ClinicalTrials.gov (No. NCT02984449).

Data sharing statement: Data are available upon reasonable request after the results have been published.

Strength and limitations of this study

- A prospective randomised open, blinded end-point (PROBE) trial
- Comparison of a unique multidisciplinary pre- and post-operative CR program with a standard care post-operative outpatient CR program
- Patient-oriented: primary and secondary outcomes provide a comprehensive and clinically relevant evaluation of patients' recovery from surgery (e.g. on physical, psychological, social domains)
- Both short- and long term effects of the CR programs are included
- Single centre study, albeit that patients are referred from four hospitals

For peer review only

For peer review only - http://bmjopen.bmj.com/site/about/guidelines.xhtml

INTRODUCTION

The leading cause of death in Western countries is ischemic heart disease.(1) One of the treatments for severe ischemic heart disease is cardiac surgery. The risk of post-operative complications related to cardiac surgery is substantial; pulmonary complications (up to 33%), delirium (~26%), and arrhythmias (~30%) have been reported to occur.(2–4) These complications are associated with prolonged hospitalisation, increased adverse events (i.e. readmission, stroke, myocardial infarction, and mortality), reduced health related quality of life (HRQoL), and higher health care costs.(5–10) Patients with poor dietary habits (present in ~80% of the candidates for cardiac surgery), who are physically inactive (~45%), who smoke (22%), or who experience depression and/or anxiety disorders (~30%) are at higher risk for post-operative complications and are at risk for lack of functional benefits after cardiac surgery.(8,11–19)

Over the last decades risk factors such as age, obesity, diabetes, hypertension and dyslipidemia have steadily increased in patients undergoing cardiac surgery.(20,21) Despite their adverse effects on treatment outcomes, reducing the burden of modifiable risk factors are currently not part of standard clinical care before and after cardiac surgery. Before cardiac surgery, patients often have a preoperative period of several weeks on the waiting list in which they receive little or no guidance. This waiting period has been associated with increased psychological stress, feelings of anxiety and reduced functional status.(22,23) With respect to inactivity during hospitalisation after cardiac surgery, research has shown that during the 8 to 11 days of hospitalisation, patients spent the majority of their time sitting or in a supine position.(24,25) In-hospital physical inactivity is a predictor of a longer hospital stay and re-hospitalisation.(24,26,27) It causes a decrease in muscle strength and aerobic capacity, both fundamental in the performance of activities of daily living.(28,29) This reduced physical capacity may seriously impact independence, especially since these patients are often elderly and the functioning of their entire physiological system is already reduced.(30)

The goal of Cardiac rehabilitation (CR) is to improve the pre- and post-operative status of patients undergoing cardiac surgery. CR aims "to favourably influence the underlying cause of cardiovascular disease, as well as to provide the best possible physical, mental, and social

For peer review only - http://bmjopen.bmj.com/site/about/guidelines.xhtml

conditions".(31) Post-operative CR is already an essential part of standard care in the Netherlands, although many hospitals in the Netherlands provide a phase II outpatient CR program, starting 3 to 6 weeks after cardiac surgery.(32,33) Benefits of post-operative CR are reported for a variety of cardiac patients,(34) however the evidence in patients undergoing cardiac surgery is still lacking with regard to patient-relevant outcomes and mortality.(35) In addition to post-operative CR, small trials suggested that preoperative CR is effective in reducing post-operative pulmonary complications, duration of hospital stay, improving HRQoL, physical fitness and increasing the compliance to post-operative CR.(23,36,37) However, long-term effects and the effects on other complications remain unclear. Furthermore, most studies investigated the effect of preoperative CR *or* post-operative CR, but not the effect of both CR methods combined. The hypothesis is that a combined pre- and post-operative CR program is more beneficial when compared to a separate preoperative CR program *or* a single post-operative CR program.

The aim of the Heart-ROCQ study is to determine the effect of a comprehensive pre- and post-operative CR program (the Heart-ROCQ program) on functional status, post-operative surgical complications, readmissions to hospital, and major adverse cardiac events (MACE) compared to a regular Dutch post-operative outpatient CR program (the Standard Care CR program).(32) In addition, the effect of the Heart-ROCQ program on physical and psychological functioning, including HRQoL, life style risk factors, work participation, and cost-effectiveness are evaluated in comparison to the Standard Care CR program. To assess who will benefit from CR and why the CR programs are effective, moderator and mediator analyses are performed.

For peer review only - http://bmjopen.bmj.com/site/about/guidelines.xhtml

Methods and analysis

Study design and organization

This investigator-initiated prospective randomised open, blinded end-point (PROBE) trial is executed in a single Centre (University Medical Centre Groningen [UMCG]). Patients from three referral hospitals (Ommelander Hospital Groningen [OZG], Martini Hospital Groningen [MZH], and Wilhelmina Hospital Assen [WZA]) who are accepted for cardiac surgery at the UMCG are also approached to participate. Patients are randomly assigned to a combined pre- and post-operative multidisciplinary CR program (the Heart-ROCQ group) or a regular phase II outpatient CR program after surgery (the Standard Care group).(32) Figure 1 provides an overview of the study design.

Ethical considerations and dissemination

This study is conducted according to the principles of the Declaration of Helsinki (version 8, October 2013) and according to the research code of the UMCG. It has been registered at ClinicalTrials.gov (No. NCT02984449) and the protocol (version 2, 2016-Dec-19) has been approved by the Medical Ethical Review Board (METc) of the UMCG (No. 2016/464). If applicable, substantial amendments will be notified to the METc for approval and changes will be written into articles describing the results of the study. Results of this study will be submitted to a peer-reviewed scientific journal and can be presented at national and international conferences.

Funding

The Heart-ROCQ study is financially supported by Edwards Lifesciences SA, Abbott (former St. Jude Medical Nederland B.V.) and 'Stichting Beatrixoord Noord-Nederland'. The sponsors are not involved in the design or execution of the study.

Participants

Patients (≥ 18 years) admitted to elective coronary artery bypass grafting (CABG), valve surgery, aortic surgery, or combined procedures are eligible. Patients accepted for congenital procedures,

For peer review only

transcatheter aortic valve implantations (TAVI), aortic dissections, or aortic descending repair are excluded. Other exclusion criteria are: inability to participate in all program elements of the Heart-ROCQ program due to disorders of the nervous or musculoskeletal system that limits exercise capacity, chronic obstructive pulmonary disease (COPD) with Global Initiative for Chronic Obstructive Lung Disease (GOLD) criteria classification 3 or 4,(38) addiction to alcohol or drugs, a serious psychiatric illness (i.e. recently experienced psychosis, bipolar disorder, diagnosis of schizophrenia, serious cognitive, or neurological problems, and acute suicidal ideations or behaviour), or when it is undesirable to exercise (i.e. hypertrophic cardiomyopathy, unstable angina, advice from cardiologist); any treatment which is planned during one of the CR programs and which is expected to interrupt attendance to the CR program (e.g. planned organ transplantation, preoperative endocarditis, or planned chemotherapy etc.); playing a sport at (inter)national level; being unable to read, write, or understand Dutch.

For peer review only

Study enrolment, randomization and registry

Figure 2 shows the flow chart of the Heart-ROCQ study. Patients on the waiting list of the thoracic surgery department and meeting the study criteria for type of surgery are asked to participate by their cardiologist. The cardiologist provides the patients with study information and an invitation to meet the researcher at the preoperative consultation. At the preoperative consultation, the researcher will obtain informed consent and conduct the baseline measurements. Eligible patients who have signed informed consent and performed the baseline measurements, are randomised to the Heart-ROCQ group or Standard Care group. Randomization (concealed group allocation in REDCap, random blocks of 2-4, 1:1 ratio) is stratified for weight of the surgery (isolated CABG, single non-CABG [i.e. replacement or repair of part of aortic or valve], 2 procedures or 3 procedures), gender, and age (≥65 and <65 years). Prior to the start of the study, the randomization lists were created (using the 'ralloc' function of Stata/SE, version 13.0) and imported into the secure data collection tool REDCap (version 7.3.2), by an independent researcher. Medical staff and researchers are not blinded to group allocation, because of logistic reasons. The primary end point is evaluated by an independent end-point committee, blinded for group allocation.

For peer review only - http://bmjopen.bmj.com/site/about/guidelines.xhtml

Patients who are not willing to participate are asked to give written consent for using data that are collected during routine care. The data are collected in the Heart-ROCQ study registry to increase insight into-potential differences between patients who participated in the study and patients who did not. The Heart-ROCQ registry will thus provide more insight into the generalizability of the results. Data from this registry are not used for the primary statistical analyses.

<u>Intervention</u>

Heart-ROCQ group

The Heart-ROCQ group receives a CR program at the Centre of Rehabilitation of the UMCG (location Beatrixoord, which is located 6 km from the hospital of the UMCG) consisting of 3 phases. The first phase is an outpatient preoperative optimization phase during the waiting period (3 times per week, minimum of 3 weeks). The second phase is a post-operative inpatient CR phase (3 weeks, weekdays only) followed by the third phase, an outpatient CR phase (2 times per week, for 4 weeks). During each phase, all participants receive physical therapy including group sessions of inspiratory muscle training (IMT),(39) strength training, aerobic cycling, breathing, coughing and relaxation. In addition, patients have an assessment with a dietician and a psychologist and take part, when indicated, in individual sessions to optimize their health. In addition, different group education sessions are organized regarding coping with stress, awareness of risk factors, and maintaining a healthy life style. Two additional modules, namely coaching to stop smoking and to return to work, are available for patients who respectively smoke or are employed. A detailed description of the CR program is given in the supplementary information.

Standard Care group

In the Netherlands, the current standard of care consists of a phase II outpatient CR program, which is conducted at the referred hospital.(32,33) In general, this CR program starts 3 to 6 weeks after discharge from the hospital and lasts for 6 weeks. The program consists of four educational sessions (regarding risk factors and retaining a healthy life style) and physical therapy (2 times per week: 30 min. cycling together with 30 min. sports and games, relaxation therapy, or strength training).

For peer review only

Furthermore, patients can be referred to a psychologist or a dietician when needed. The content of this CR program is based on the Dutch CR guidelines.(40)

Composite primary end point: functional status, complications and events

The primary outcome is a composite weighted score of functional status, post-operative surgical complications, readmissions to the hospital, and MACE. Each event is scored (ranging from 1 to 3 points), separately. Table 1 shows an overview of the primary outcome and the scoring system. The scores of all events are combined to calculate a total score. Only the most serious complication is counted per event (e.g. when a percutaneous coronary intervention [score 1] is complicated by a stroke [score 2], the score will be 2 and not 3 [1 + 2]).

The concept of the composite weighted score is adapted from the African-American Heart Failure Trial (A-HeFT).(41) Functional status is assessed through two health domains of the Medical Outcome Study 36-item General Health Survey (RAND-36 version 2)(42): physical functioning and physical health problems. The primary end point is evaluated at 3 months (T4) and 1 year (T6) after surgery (Figure 1). Deep wound infections and surgical re-explorations are screened up to 120 days, after surgery. Other post-operative surgical complications are measured in the period between the surgery and when a patient meets the UMCG discharge criteria (Table 2). Hospital admissions are checked between baseline and one year after surgery. To prevent bias (since the Heart-ROCQ group follows the in-patient CR phase after surgery) hospital admissions between the day of admission before surgery and 30 days after surgery are not included when determining the (calculated) primary end point.

Secondary outcomes

Complications and events

All individual components of the composite end point regarding the complications and events will be analysed separately as secondary outcomes. Table 3 summarizes the definitions of the secondary complications and events, including at the time of screening.

For peer review only

For peer review only - http://bmjopen.bmj.com/site/about/guidelines.xhtml

All documents concerning the composite primary end point and the secondary complications are encrypted and subsequently adjudicated by the independent end-point committee. The end-point committee consists of four members (cardiologists and cardiothoracic surgeons), who are not employed in the UMCG and are blinded for group allocation.

For peer review only

Table 1: definitions and score of the components of the composite primary end point

**Functional status**	**Score**
Worsening in physical functioning (*domain score of health related quality of life, rand36_v2)*†	1
No change or improvement in physical functioning†	0
Worsening in physical problem (*domain score of health related quality of life, rand36_v2)*†	1
No change or improvement in physical problem†	0
A clinical relevant worsening is classified as minimal change according to *Wyrwich et al., 2004*(43)	
**(Serious) Adverse Events**	
No serious adverse event	0
Prolonged mechanical ventilation<br>*Mechanical ventilation longer than 24 hours*	1
Lung infection<br>*1) A new lung radiographic infiltrate AND 2) two signs that the infiltrate is infectious origin (i.e. a) fever - body temperature >38°C or <36°C -, b) leukocytosis - white blood cells >10,000 or <4000, c) positive sputum culture AND/OR d) decline in oxygenation*	1
Delirium<br>*1) A DOS score ≥ 3 at hospital ward AND/OR 2) Diagnosis confirmed by a psychiatrist, geriatrist, or supervising specialist according to the DSM-IV criteria resulting in treatment with medication*	1
Readmissions to intensive care unit<br>*Unrelated to a secondary end point*	1
Deep wound infection<br>*Deeper tissues are affected (muscle, sternum, and mediastinum) and must include: 1) surgical drainage / re-fixation OR 2) an organism is isolated from culture of mediastina tissue or fluid, OR 3) antibiotic treatment, because of sternum wound*	2
Readmissions to hospital<br>*An unplanned hospital stay with different dates of admission and discharge with a medical indication (i.e. clinical signs and symptoms or change of treatment)*	1
Any cardiothoracic surgical interventions<br>*Graft- or valve failure, CABG, valve, aortic, or other cardiac surgery*‡	2
Any percutaneous interventions<br>*PCI, TAVI, etc.*	1
Myocardial infarction<br>*According to the third universal definition of myocardial infarction*§	2
Cerebral vascular accident / stroke<br>*Acute neurological event of at least 24 hours of duration, with focal signs and symptoms and without evidence supporting any alternative explanation. Diagnosis of stroke requires confirmation by CT, MRI, or pathological confirmation.*	2
Sudden death survivor<br>*The sudden onset of symptoms, such as chest pain and cardiac arrhythmias, ventricular tachycardia, which lead to the loss of consciousness and cardiac arrest followed by reanimation and does not lead to biological death.*	2
Death<br>*All-cause mortality*	3
Total score = *sum of physical status and SAE's at 3 months and 1 year (worst score of each event)*	

†Compared with baseline; ‡According to the definitions of the 'Begeleidingscommissie Hartinterventies Nederland; §Thygesen et al., 2012(44); DOS: Delirium Observation Screening scale(45); CABG: Coronary arterial bypass graft; PCI: Percutaneous coronary intervention; TAVI: Transcathetic aortic valve implantation; In gray: post-operative complications can be scored ones; SAE: Serious adverse event.

For peer review only

For peer review only - http://bmjopen.bmj.com/site/about/guidelines.xhtml

Table 2: Discharge criteria of the University Medical Centre Groningen (UMCG)

1) no drain, no external pacemaker lead, no infusion, or oxygen present,

2) stable clinical conditions (stable lab results, X-ray, and haemodynamic parameters),

3) able to perform basic activity of daily living (ADL)-activities (i.e. going independently to the toilet).

Table 3: definitions of the secondary complications and events

**Definitions**	**Time of measure**
Atrial fibrillation<br>*New onset of atrial fibrillation or atrial flutter requiring medical treatment or cardioversion*	Surgery to T3
Prolonged ICU stay<br>*When the number of calendar days is two or more from ICU admission to discharge*	Initial stay
Readmissions to hospital<br>*The number of unplanned hospital stays with different dates of admission and discharge with a medical indication (i.e. clinical signs and symptoms or change of treatment)*<br>*a) directly related to a cardiac cause*<br>*b) not directly related to a cardiac cause*	Baseline to T6[§]
Hospitalisation days<br>*Total number of days of hospitalisation*<br>*a) directly related to a cardiac cause*<br>*b) not directly related to a cardiac cause*	Baseline to T6[§]
Cardiovascular death<br>*Any death due to proximate cardiac or cardiovascular cause (e.g. Myocardial infarct, low-output failure, fatal arrhythmia, death secondary to a cerebral vascular accident, pulmonary embolism, ruptured or dissecting aortic aneurysm, or other vascular diseases), un-witnessed death, and death of unknown cause, and all procedure related deaths (e.g. PCI, CABG), including those related to concomitant treatment[†].*	Baseline to 5 years after surgery
Non-cardiovascular death<br>*Any death not covered by above definition, such as death caused by infection, malignancy, sepsis, pulmonary causes, accident, suicide, or trauma*	Baseline to 5 years after surgery
**Concerning safety**	
Surgical re-exploration for bleeding/ tamponade<br>*Surgical incision into the sternum as a result of a bleeding or tamponade*<br>*a) acute: presented within 24 hours after surgery*<br>*b) late: presented after 24 hours after surgery*	Surgery to T4
Surgical re-exploration dehiscence<br>*Aseptic wound dehiscence*	Surgery to T4

[†]Specifically, any unexpected death even in patients with coexisting potentially fatal non-cardiac disease (e.g. cancer, infection) will be classified as cardiac death; [§]Not measured between the day of admission before surgery and 30 days after surgery, because of POST-in phase of Heart-ROCQ program; ICU: Intensive care unit; T4: Follow-up at 3 months after surgery; T6: Follow-up at 12 months after surgery.

For peer review only

Questionnaires and physical tests

Table 4 shows the physical tests and questionnaires of the secondary outcomes regarding physical and psychological health, life style risk factors, and the economic evaluation. Physical tests and questionnaires are completed at six assessment points (Figure 1). The preoperative measurements (T1 and T2) are conducted at the start (preoperative consultation) and the end (1 to 8 days before surgery) of the waiting period. The third measurement (T3) is performed when patients meet the UMCG discharge criteria (Table 2). The follow-up measurements (T4, T5, and T6) are at 3, 7, and 12 months after surgery. Patients are asked to fill in questionnaires online or when requested on paper, prior to the visits for the physical tests. All adverse events reported spontaneously by the patient or observed by the investigator are recorded. In addition, serious adverse events are reported to the METc. Data are stored in REDCap and on a secure drive at the department CardioResearch in the UMCG. Two times per year, the study is monitored by a trained research monitor from another department of the UMCG. Details of procedures, data collection, management and monitoring can be found in the Trial Master File of the Heart-ROCQ study (can be obtained by the investigators). On the 12th of June 2018, the study was audited and certified for the ISO 9001: 2015 independently by DNV GL.

Potential moderators

Preoperative risk profile (i.e. Euroscore II, medical history, life style risk factors, physical, and mental status), surgery parameters, and demographics are collected form the medical record and baseline measurements. The content of the CR program is described in terms of compliance, duration of CR program, type of treatment, frequency, and training load (i.e. for bicycle training: external workload, heart rate response, rate of perceived exertion using the Borg scale, and for strength training: sets, repetitions, and intensity) for both the Heart-ROCQ group as the Standard Care group.

For peer review only - http://bmjopen.bmj.com/site/about/guidelines.xhtml

Table 4: Time of measure, physical tests, and questionnaires of the secondary outcomes

Secondary outcomes	Measure	Time of measure
Physical health		
Cardiorespiratory fitness	6MWT(46)	T1-T4, T6
Muscle strength	STS_10, grip & leg strength(47,48)	T1-T4, T6
Independence in ADL	KATZ(49,50)	T1, T4, T6
Psychological health		
General anxiety	GAD-7(51)	T1,T2, T4, T6
Feelings of depression	PHQ-9(52,53)	T1,T2, T4, T6
Health related Quality of Life	Rand-36_v2(42)	T1, T4, T6
Life style risk factors		
Physical activity	iPAQ(54) & Actigraph†(55)	T1, T4, T6
Obesity indices	BMI, waist-to-hip ratio	T1, T4, T6
Smoking behaviour	Number of cigarettes per day	T1, T4, T6
Economic evaluation		
Health care use & related medical costs	iMCQ(56)	T1, T4-T6
Work participation‡	iPCQ(57)	T1, T4-T6
QALYs	EQ-5D-5L(58)	T1, T4-T6
Potential mediators		
Cardiac self-efficacy	CSE(59)	T1, T2, T4, T6
Illness representations	IPQ-R(60)	T1, T2, T4, T6

†Patients are asked to wear the Actigraph during waking hours for 1 consecutive week; ‡Health-related productivity losses of paid work and unpaid work; T1: Begin of waiting time; T2: 1 to 8 days before surgery; T3: Moment that patients meet the UMCG discharge criteria; T4-T6: Follow-up at 3, 7, and 12 months after surgery; 6MWT: Six minutes walking test; STS_10: Ten times sit to stand test; ADL: Activities of Daily Living; KATZ: Katz Index of Independence in Activities of Daily Living; GAD-7: Generalized Anxiety Disorder 7-item scale; PHQ-9: Patient Health Questionnaire 9-item scale; iPAQ: International Physical Activity Questionnaire; BMI: Body Mass Index; iMCQ: iMTA Medical Cost Questionnaire; iPCQ: iMTA Productivity Cost Questionnaire; QALY: Quality-adjusted life years; EQ-5D-5L: EuroQol five-dimensional questionnaire; CSE: Cardiac Self-efficacy; IPQ-R: Illness Perception Questionnaire, Revised.

<u>Potential mediators</u>

Illness perceptions (i.e. patients mental representations of their illness based on different sources of information) are measured with three subscales (personal control, treatment control, and consequences) of the revised illness perception questionnaire (IPQ-R).(60) These subscales are chosen because of their sensitivity to change and because of their relation to psychological distress(61). The cardiac self-efficacy (CSE) scale(59) is used to measure CSE (i.e. patients confidence to perform a specific task related to cardiac disease).

For peer review only

Statistical analyses

Sample size

Assuming a normal distribution, the mean weighted score of the primary end point is estimated on 1.0 with a standard deviation of 0.9 at 1 year after surgery. This estimation is based on historical data of the UMCG, an unpublished on-going pilot study in the UMCG, the Dutch registration database,(62) and data reported in literature.(6,63–65) A decrease of 0.3 is expected in the Heart-ROCQ group, based on previous studies comparing CR with standard care(36,64,66) (i.e. no CR) and is considered to be clinically relevant (i.e. on average a 30% decrease in complications/events or worsening in functional status or 10-20% decrease in MACE or death). To detect this decrease and achieve 80% power (significance level of 5%) a group of 286 patients (143 in the Heart-ROCQ group and 143 in the Standard Care group) is needed. To incorporate a withdrawal of ±20% a total sample size of 350 is needed at baseline.

Interim analysis

An interim analysis will be conducted when 40% of the included patients have had the measurements one year after surgery. The study will be terminated prematurely when the primary outcome of one of the CR programs is obviously ($P<0.001$) different from the other CR program.

Primary and sensitivity analyses

All end points are primary analysed according to the ‘intention-to-treat’ principles and missing values are counted as worst-case score (nominal variables) or estimated using Maximum Likelihood estimation (interval variables). As supplementary analyses, the end points are analysed on a per-protocol principle with and without using imputation methods for missing values. In all analyses, a two-sided $p<0.05$ is considered statistically significant.

Statistical methods and clinical relevance for analysing secondary outcomes are written in the research protocol (ClinicalTrials.gov : NCT02984449).

For peer review only - http://bmjopen.bmj.com/site/about/guidelines.xhtml

Economic evaluation

For the evaluation of health-care utilization, standard prices published in the Dutch costs guidelines are used.(67) To compare the costs to quality adjusted life years (QALYs), QALYs are estimated with use of the EQ-5D-5L.(58) Utility values for the EQ-5D-5L are calculated based on the new Dutch tariff.(68) Results from the analysis are reported as an Incremental Cost Effectiveness Ratio (ICER), dividing the difference in effect, by the difference in costs. Bootstrap re-sampling will be performed, and cost-effectiveness acceptability curves will be plotted, to estimate the probability that the Heart-ROCQ program is cost effective when compared to standard care. A societal perspective is applied.

Study status

From May 2017 to December 2018, 75 patients were enrolled. In following years we expect that the enrolment will increase to 85 patients per year. The last patient is expected to be included in July 2021.

Patient and public involvement

In a pilot study (to be submitted) the patient satisfaction and the feasibility (in terms of accessibility, compliance, training load and safety) of the Heart-ROCQ program have been evaluated. Patients were very satisfied with the program and scoring it eight out of ten, therefore we did not change the content of the program. However, patients' rate of perceived exertion was generally quite low and no serious adverse events occurred during the bicycle training. For safety reasons the intensity was not increased. However, in order to estimate the maximum load more accurately and better tailor the program to the individual, we decided to change one of the stop criteria of the preoperative submaximal ergometry test from 70% to 90% of expected maximal heart rate. Furthermore, our outcomes are, among others, based on the reasons why patients recommended the program to other patients. For example, patients reported that their self-efficacy and physical capacity were improved, so we added the CSE questionnaire and physical tests to objectively measure these outcomes. In this way the results were taken into account in the further development of the Heart-ROCQ program and the protocol of this study. The results of this trial will be distributed by various

For peer review only - http://bmjopen.bmj.com/site/about/guidelines.xhtml

information channels (e.g. websites of cardiac patient organisations, social media). Two to three times a year we provide a newsletter about the progress and (in the end) the results of the study are sent to patients, who are interested.

For peer review only

For peer review only - http://bmjopen.bmj.com/site/about/guidelines.xhtml

Discussion

The Heart-ROCQ study is the first randomised clinical trial evaluating the effect of a combination of pre- and post-operative CR program compared to a post-operative CR program. Unlike the vast majority of CR programs in previous studies, the current program is multidisciplinary targeting different aspects of surgical outcomes in patients undergoing cardiac surgery. Because different aspects are targeted, a composite weighted score will better reflect the treatment benefits than a single outcome. Therefore, the primary end point is a composite end point of functional status, post-operative surgical complications, readmissions to the hospital and MACE. The components of the end point are of clinical importance to patients undergoing cardiac surgery and reflect a comprehensive representation of the recovery of the patient.

Both the ideas of the combined primary end point and the weighting of the individual components were derived from other studies.(41,69,70) Assigning different weights to the components was needed for more accurate comparison. Since the components are not equal in clinical importance, equal weights would lead to inaccurate statistical analysis(70). In the current study, the weighted score of HRQoL is lower (1 point instead of 2 points) to minimize bias due to patients' knowledge of group allocation. Therefore, improvements in quality of life are not counted in the primary end point to prevent bias in a positive direction. This also prevents that a score in quality of life and adverse event cancel each other out (e.g. when a patient experiences an improvement in Quality in Life [+2 points] and has a stroke [-2 points] then the total score is 0). The scores of all-cause mortality, stroke, myocardial infarction and revascularization are in line with the results showed by Tong and colleagues (2013)(70). A disadvantage of composite end points is that the effect may be driven by complications that occur with the greatest frequency.(71) Therefore, post-operative complications which occur frequently, such as atrial fibrillation, are evaluated separately as secondary end point. The primary end point is evaluated by an end-point committee, which is blinded to group allocation and consists of four independent cardiologists and cardio-thoracic surgeons.

Previous preoperative CR studies were primarily focused on short-term effects; only one preoperative CR study and a few post-operative CR studies have determined long-term

For peer review only

effects.(23,35,36,72) In contrast to previous preoperative CR studies, the Heart-ROCQ study sets out to investigate both short- and long term effects of the CR programs.(23,35,36) Due to the trends in, among others, increasing age, obesity, and physical inactivity, patients undergoing cardiac surgery are becoming more complex. The Heart-ROCQ program aims to address these issues, which make the program clinically relevant for all cardiac surgery patients. Therefore, we chose to include different types of cardiac surgery. Since different moderators and mediators are assessed before, during and after CR, we can explore which factors are associated with better outcomes and which working mechanisms contribute to its effectiveness. These findings may provide a more in-depth understanding of who benefits the most from CR in both the short and long term and the underlying mechanisms of CR, which are still not fully understood in patients undergoing cardiac surgery.(35) In addition, the present study is thought to considerably contribute to the evidence to further develop guidelines for clinical practice, especially regarding the preoperative CR program.(73)

The Heart-ROCQ program is expected to be cost-effective in the long-term, which is also of interest for policymakers and health care providers. Therefore, an economic evaluation is performed to assess the cost-effectiveness of CR, since little is known about the cost-effectiveness of CR.(64) A societal perspective of this economic evaluation is chosen, meaning that not only health-care costs, but also patient- and productivity related costs and benefits are taken into account. If the Heart-ROCQ program is proven to be effective, it might be advisable to include a supportive prevention-oriented waiting period prior to surgery as an integral part of the treatment for patients undergoing elective cardiac surgery. This implies a paradigm shift from curative care following cardiac surgery to an additional preventive care attitude before surgery. Extensions of rehabilitation options in, or in the vicinity of cardiac centres, will then be required.

The Heart-ROCQ study is the first randomised clinical trial comparing the effect of a combined pre- and post-operative CR program to a regular Dutch phase II post-surgery outpatient rehabilitation CR program in a population undergoing elective cardiac surgery. This study is expected to provide new understanding of the effectiveness and underlying working mechanisms of CR, and subsequently to improve value-based health care.

For peer review only

For peer review only - http://bmjopen.bmj.com/site/about/guidelines.xhtml

Acknowledgements

We thank E.J. Beens, M.J. Nijholt, Drs. A. Branderhorst, Dr. B. Dorhout (in memoriam), Dr. R. de Jong, H.A.M. Wasser, Dr. J. Brügemann, Dr. J.B. Wempe, Dr. J. Posma, Dr. C.A. Geluk, and Drs. M.L. Pentinga supporting the Heart-ROCQ study. In addition, we thank Drs. T.W. Waterbolk, Dr. R.G. Tieleman, and Dr. R.P.M. van Roosmalen for their participation in the end-point committee. We are grateful to all involved colleagues in the department of Cardiology, Cardio-thoracic Surgery, and Centre for Rehabilitation of the UMCG, OZG, MZH, and WZA for the pleasant collaboration and their help with executing the Heart-ROCQ study.

For peer review only

Declaration of interest

Drs. J. Hartog, Drs. S. Dijkstra, and Dr. M.A. Mariani report grants from Edwards Lifesciences SA, Abbott (former St. Jude Medical Nederland B.V.), and 'Stichting Beatrixoord Noord-Nederland' related to this study. In addition, Dr. M.A. Mariani reports consultancy from AtriCure, Getinge, and LivaNova. Dr. J. Fleer, Dr. W. Dieperink, Dr. L.H.V. van der Woude, Dr. M.F. Reneman, Drs. F. Blokzijl, Dr. I.C.C. van der Horst, Dr. M.J.L. DeJongste, and Dr. P. van der Harst have nothing to disclose.

Authors'contributions

Conception and design of the study: JH, FZ, MJLdJ, MFR, WD, JF, LHVvdW, PvdH, and MAM; Methodology: JH, FB, SD, MJLdJ, MFR, and PvdH; Acquisition of data: JH, FB and SD; Writing – Original draft, tables, and figures: JH; Writing- Reviewing and Editing: FB, SD, MJLdJ, MFR, WD, ICCvdH, JF, LHVvdW, PvdH, and MAM; Supervision: MJLdJ, MFR, WD, JF, ICCvdH, LHVvdW, PvdH, and MAM; Funding Acquisition: JH, SD, JF, LHVvdW, PvdH, and MAM. All authors approved the final version of the manuscript.

For peer review only - http://bmjopen.bmj.com/site/about/guidelines.xhtml

REFERENCES

1. GBD 2016 Causes of Death Collaborators. Global, regional, and national age-sex specific mortality for 264 causes of death, 1980–2016: a systematic analysis for the Global Burden of Disease Study 2016. Lancet. 2017;390(10100):1151–210.

2. Hulzebos EHJ, Van Meeteren NLU, De Bie RA, Dagnelie PC, Helders PJM. Prediction of postoperative pulmonary complications on the basis of preoperative risk factors in patients who had undergone coronary artery bypass graft surgery. Phys Ther. 2003;83(1):8–16.

3. Kassie GM, Nguyen TA, Kalisch Ellett LM, Pratt NL, Roughead EE. Preoperative medication use and postoperative delirium: a systematic review. BMC Geriatr. 2017 Dec 29;17(1):298.

4. Weymann A, Popov AF, Sabashnikov A, Ali-Hasan-Al-Saegh S, Ryazanov M, Tse G, et al. Baseline and postoperative levels of C-reactive protein and interleukins as inflammatory predictors of atrial fibrillation following cardiac surgery: a systematic review and meta-analysis. Kardiol Pol. 2018 Feb 16;76(2):440–51.

5. Koster S, Hensens AG, Schuurmans MJ, van der Palen J. Consequences of delirium after cardiac operations. Ann Thorac Surg. 2012 Mar;93(3):705–11.

6. Iribarne A, Chang H, Alexander JH, Gillinov AM, Moquete E, Puskas JD, et al. Readmissions after cardiac surgery: experience of the National Institutes of Health/Canadian Institutes of Health research cardiothoracic surgical trials network. Ann Thorac Surg. 2014 Oct;98(4):1274–80.

7. Villareal RP, Hariharan R, Liu BC, Kar B, Lee V-V, Elayda M, et al. Postoperative atrial fibrillation and mortality after coronary artery bypass surgery. J Am Coll Cardiol. 2004 Mar 3;43(5):742–8.

8. Hashemzadeh K, Dehdilani M, Dehdilani M. Postoperative Atrial Fibrillation following Open Cardiac Surgery: Predisposing Factors and Complications. J Cardiovasc Thorac Res. 2013;5(3):101–7.

9. Kosuma P, Wachirasrisirikul S, Jedsadayanmata A. Attributable Costs of Postoperative Atrial Fibrillation among Patients Undergoing Cardiac Surgery. Cardiol Res Pract. 2018;2018:1–5.

10. Findeisen A, Arefian H, Doenst T, Hagel S, Pletz MW, Hartmann M, et al. Economic burden of surgical site infections in patients undergoing cardiac surgery. Eur J Cardio-Thoracic Surg. 2018;0(8):1–7.

11. Ruiz-Núñez B, van den Hurk GHAM, de Vries JHM, Mariani MA, de Jongste MJL, Dijck-Brouwer DAJ, et al. Patients undergoing elective coronary artery bypass grafting exhibit poor pre-operative intakes of fruit, vegetables, dietary fibre, fish and vitamin D. Br J Nutr. 2015;(6):1–11.

12. Zittermann A, Kuhn J, Dreier J, Knabbe C, Gummert JF, Börgermann J. Vitamin D status and the risk of major adverse cardiac and cerebrovascular events in cardiac surgery. Eur Heart J. 2013;34(18):1358–64.

13. Kavanagh T, Mertens DJ, Hamm LF, Beyene J, Kennedy J, Corey P, et al. Prediction of long-term prognosis in 12 169 men referred for cardiac rehabilitation. Circulation. 2002;106(6):666–71.

14. van Laar C, Timman ST, Noyez L. Decreased physical activity is a predictor for a complicated recovery post cardiac surgery. Health Qual Life Outcomes. 2017;15(1):1–7.

15. Christian LM, Graham JE, Padgett DA, Glaser R, Kiecolt-Glaser JK. Stress and wound

For peer review only

For peer review only - http://bmjopen.bmj.com/site/about/guidelines.xhtml

healing. Neuroimmunomodulation. 2007;13(5–6):337–46.

16. Tully PJ, Baker RA. Depression, anxiety, and cardiac morbidity outcomes after coronary artery bypass surgery: a contemporary and practical review. J Geriatr Cardiol. 2012 Jun;9(2):197–208.

17. Kehler DS, Stammers AN, Tangri N, Hiebert B, Fransoo R, Schultz ASH, et al. Systematic review of preoperative physical activity and its impact on postcardiac surgical outcomes. BMJ Open. 2017;

18. Zhang YJ, Iqbal J, Van Klaveren D, Campos CM, Holmes DR, Kappetein AP, et al. Smoking is associated with adverse clinical outcomes in patients undergoing revascularization with PCI or CABG: The SYNTAX trial at 5-year follow-up. J Am Coll Cardiol. 2015;65(11):1107–15.

19. Mallik S, Krumholz HM, Zhen QL, Kasl S V., Mattera JA, Roumains SA, et al. Patients with depressive symptoms have lower health status benefits after coronary artery bypass surgery. Circulation. 2005;111(3):271–7.

20. Ghanta RK, Kaneko T, Gammie JS, Sheng S, Aranki SF. Evolving trends of reoperative coronary artery bypass grafting: An analysis of the Society of Thoracic Surgeons Adult Cardiac Surgery Database. J Thorac Cardiovasc Surg. 2013;145(2):364–72.

21. Kindo M, Hoang Minh T, Perrier S, Bentz J, Mommerot A, Billaud P, et al. Trends in isolated coronary artery bypass grafting over the last decade. Interact Cardiovasc Thorac Surg. 2017;24(1):71–6.

22. Carr T, Teucher U, Mann J, Casson AG. Waiting for surgery from the patient perspective. Psychol Res Behav Manag. 2009;2:107–19.

23. Arthur HM, Daniels C, McKelvie R, Hirsh J, Rush B. Effect of a preoperative intervention on preoperative and postoperative outcomes in low-risk patients awaiting elective coronary artery bypass graft surgery: A randomized, controlled trial. Ann Intern Med. 2000;133(4):253–62.

24. Van Der Peijl ID, Vliet Vlieland TPM, Versteegh MIM, Lok JJ, Munneke M, Dion RAE. Exercise therapy after coronary artery bypass graft surgery: A randomized comparison of a high and low frequency exercise therapy program. Ann Thorac Surg. 2004;77(5):1535–41.

25. Koopman C, Van Dis I, Vaartjes I, Visseren FLJ, Bots ML. Hart- en vaatziekten in Nederland 2014, cijfers over kwaliteit van leven, ziekte en sterfte. Harstichting. 2013. p. 192.

26. Takahashi T, Kumamaru M, Jenkins S, Saitoh M, Morisawa T, Matsuda H. In-patient step count predicts re-hospitalization after cardiac surgery. J Cardiol. 2015;66(4):286–91.

27. Cook DJ, Thompson JE, Prinsen SK, Dearani JA, Deschamps C. Functional recovery in the elderly after major surgery: Assessment of mobility recovery using wireless technology. Ann Thorac Surg. 2013;96(3):1057–61.

28. Kortebein P, Symons TB, Ferrando A, Paddon-Jones D, Ronsen O, Protas E, et al. Functional impact of 10 days of bed rest in healthy older adults. J Gerontol A Biol Sci Med Sci. 2008;63(10):1076–81.

29. Convertino V, Hung J, Goldwater D, DeBusk RF. Cardiovascular responses to exercise in middle-aged men after 10 days of bedrest. Circulation. 1982 Jan;65(1):134–40.

30. Chodzko-Zajko WJ, Proctor DN, Fiatarone Singh MA, Minson CT, Nigg CR, Salem GJ, et al. Exercise and physical activity for older adults. Med Sci Sports Exerc. 2009;41(7):1510–30.

31. BACPR. The British Association for Cardiovascular Prevention and Rehabilitation (BACPR)

For peer review only

standards and core components for cardiovascular disease prevention and rehabilitation, 2nd edition, 2012. 2012.

32. Bjarnason-Wehrens B, McGee H, Zwisler AD, Piepoli MF, Benzer W, Schmid J-P, et al. Cardiac rehabilitation in Europe: results from the European Cardiac Rehabilitation Inventory Survey. Eur J Cardiovasc Prev Rehabil. 2010;17(4):410–8.

33. De Vries H, Kemps HMC, Van Engen-Verheul MM, Kraaijenhagen RA, Peek N. Cardiac rehabilitation and survival in a large representative community cohort of Dutch patients. Eur Heart J. 2015;36:1519–28.

34. Anderson L, Taylor RS. Cardiac rehabilitation for people with heart disease: an overview of Cochrane systematic reviews. Cochrane database Syst Rev. 2014 Jan;12:CD011273.

35. Blokzijl F, Dieperink W, Keus F, Reneman MF, Mariani MA, van der Horst IC. Cardiac rehabilitation for patients having cardiac surgery: a systematic review. J Cardiovasc Surg (Torino). 2018 Apr 3;

36. Hulzebos EHJ, Smit Y, Helders PPJM, van Meeteren NLU. Preoperative physical therapy for elective cardiac surgery patients. Cochrane database Syst Rev. 2012 Jan;11:CD010118.

37. Sawatzky JA V, Kehler DS, Ready AE, Lerner N, Boreskie S, Lamont D, et al. Prehabilitation program for elective coronary artery bypass graft surgery patients: a pilot randomized controlled study. Clin Rehabil. 2014 Jan 23;28(7):648–57.

38. Vogelmeier CF, Criner GJ, Martinez FJ, Anzueto A, Barnes PJ, Bourbeau J, et al. Global Strategy for the Diagnosis, Management and Prevention of Chronic Obstructive Lung Disease 2017 Report. Respirology. 2017;22(3):575–601.

39. van Adrichem EJ, Meulenbroek RL, Plukker JTM, Groen H, van Weert E. Comparison of two preoperative inspiratory muscle training programs to prevent pulmonary complications in patients undergoing esophagectomy: a randomized controlled pilot study. Ann Surg Oncol. 2014 Jul;21(7):2353–60.

40. Van Dijk J. Multidisciplinaire Richtlijn Hartrevalidatie 2011. Tijdschr VOOR BEDRIJFS- EN Verzek. 2011;19(9):410–5.

41. Taylor AL, Ziesche S, Yancy C, Carson P, D'Agostino R, Ferdinand K, et al. Combination of Isosorbide Dinitrate an Hydralazine in Blacks with Heart Failure. N Engl J Med. 2004;351(20):2049–57.

42. Van der Zee KI, Sanderman R, Heyink JW, de Haes H, Van der Zee KI, Sanderman R, et al. Psychometric qualities of the RAND 36-Item Health Survey 1.0: a multidimensional measure of general health status. Int J Behav Med. 1996 Jan;3(2):104–22.

43. Wyrwich KW, Spertus JA, Kroenke K, Tierney WM, Babu AN, Wolinsky FD. Clinically important differences in health status for patients with heart disease: An expert consensus panel report. Am Heart J. 2004;147(4):615–22.

44. Thygesen K, Alpert JS, Jaffe AS, Simoons ML, Chaitman BR, White HD, et al. Third universal definition of myocardial infarction. Eur Heart J. 2012;33(20):2551–67.

45. Koster S, Hensens AG, Oosterveld FGJ, Wijma A, van der Palen J. The delirium observation screening scale recognizes delirium early after cardiac surgery. Eur J Cardiovasc Nurs. 2009;8(4):309–14.

46. Crapo RO, Casaburi R, Coates AL, Enright PL, MacIntyre NR, McKay RT, et al. ATS statement: Guidelines for the six-minute walk test. Am J Respir Crit Care Med.

For peer review only

For peer review only - http://bmjopen.bmj.com/site/about/guidelines.xhtml

2002;166(1):111–7.

47. Puthoff ML, Saskowski D. Reliability and responsiveness of gait speed, five times sit to stand, and hand grip strength for patients in cardiac rehabilitation. Cardiopulm Phys Ther J. 2013 Mar;24(1):31–7.

48. Douma KW, Regterschot GRH, Krijnen WP, Slager GEC, van der Schans CP, Zijlstra W. Reliability of the Q Force; a mobile instrument for measuring isometric quadriceps muscle strength. BMC Sports Sci Med Rehabil. 2016 Jan;8:4.

49. Katz S, Ford AB, Moskowitz RW, Jackson BA, Jaffe MW, Cleveland MA. Studies of Illness in the Aged. The Index of ADL: A Standardized Measure of Biological and Psychosocial Function. JAMA. 1963;185(12):914–9.

50. Reijneveld SA, Spijker J, Dijkshoorn H. Katz' ADL index assessed functional performance of Turkish, Moroccan, and Dutch elderly. J Clin Epidemiol. 2007;60(4):382–8.

51. Spitzer RL, Kroenke K, Williams JBW, Löwe B. A Brief Measure for Assessing Generalized Anxiety Disorder: the GAD-7. Arch Intern Med. 2006;166(10):1092–7.

52. Kroenke K, Spitzer RL, Williams JB. The PHQ-9: validity of a brief depression severity measure. J Gen Intern Med. 2001 Sep;16(9):606–13.

53. Löwe B, Unützer J, Callahan CM, Perkins AJ, Kroenke K. Monitoring depression treatment outcomes with the patient health questionnaire-9. Med Care. 2004 Dec;42(12):1194–201.

54. Craig CL, Marshall AL, Sjöström M, Bauman AE, Booth ML, Ainsworth BE, et al. International physical activity questionnaire: 12-Country reliability and validity. Med Sci Sports Exerc. 2003;35(8):1381–95.

55. Whitaker KM, Gabriel KP, Jacobs DR, Sidney S, Sternfeld B. Comparison of Two Generations of ActiGraph Accelerometers: The CARDIA Study. Med Sci Sport Exerc. 2018;50(6):1333–40.

56. Bouwmans C, Hakkaart-van Roijen L, Koopmanschap M, Krol M, Severens H BW. Medical Cost Questionnaire (iMCQ). Rotterdam: iMTA, Erasmus Universiteit Rotterdam. 2013 [cited 2016 May 1]. Available from: https://www.imta.nl/

57. Bouwmans C, Krol M, Severens H, Koopmanschap M, Brouwer W, Roijen LH Van. The iMTA Productivity Cost Questionnaire: A Standardized Instrument for Measuring and Valuing Health-Related Productivity Losses. Value Heal. 2015;18(6):753–8. Available from: http://dx.doi.org/10.1016/j.jval.2015.05.009

58. EuroQol Group. What is EQ-5D? 2014 [cited 2016 Nov 3]. Available from: http://www.euroqol.org/

59. Sullivan MD, LaCroix AZ, Russo J, Katon WJ. Self-efficacy and self-reported functional status in coronary heart disease: a six-month prospective study. Psychosom Med. 1998;60(4):473–8.

60. Moss-Morris R, Weinman J, Petrie K. The revised illness perception questionnaire (IPQ-R). Psychol Heal. 2002;17(1):1–16.

61. Morgan K, Villiers-Tuthill A, Barker M, McGee H. The contribution of illness perception to psychological distress in heart failure patients. BMC Psychol. 2014 Nov 28;2(1):50.

62. Meetbaar Beter Boek 2015 [cited 2016 Apr 25]. p. 60. Available from: http://www.meetbaarbeter.com/documents/meetbaar-beter-boeken/

63. Engblom E, Korpilahti K, Hämäläinen H, Rönnemaa T, Puukka P. Quality of life and return to

For peer review only

For peer review only - http://bmjopen.bmj.com/site/about/guidelines.xhtml

work 5 years after coronary artery bypass surgery. Long-term results of cardiac rehabilitation. J Cardiopulm Rehabil. 1997 Jan;17(1):29–36.

64. Heran BS, Chen JM, Ebrahim S, Moxham T, Oldridge N, Rees K, et al. Exercise-based cardiac rehabilitation for coronary heart disease. Cochrane database Syst Rev. 2011 Jul 6;(7):CD001800.

65. Serruys PW, Morice M, Kappetein AP, Colombo A, Holmes DR, Mack MJ, et al. Percutaneous Coronary Intervention versus Coronary-Artery Bypass Grafting for Severe Coronary Artery Disease. N Engl J Med. 2009;360:961–72.

66. Jolliffe JA, Rees K, Taylor RS, Thompson D, Oldridge N, Ebrahim S. Exercise-based rehabilitation for coronary heart disease. Cochrane database Syst Rev. 2001 Jan;(1):CD001800.

67. Zorginstituut Nederland. Kostenhandleiding: Methodologie van kostenonderzoek en referentieprijzen voor economische evaluaties in de gezondheidszorg. 2015.

68. Versteegh MM, Vermeulen KM, Evers SMAA, de Wit GA, Prenger R, Stolk EA. Dutch Tariff for the Five-Level Version of EQ-5D. Value Health. 2016 Jun;19(4):343–52.

69. de Vries AE, de Jong RM, van der Wal MH, Jaarsma T, van Dijk RB, Hillege HL. The value of INnovative ICT guided disease management combined with Telemonitoring in OUtpatient clinics for Chronic Heart failure patients. Design and methodology of the IN TOUCH study: a multicenter randomised trial. BMC Health Serv Res. 2011;11(1):167.

70. Tong BC, Huber JC, Ascheim DD, Puskas JD, Ferguson Jr B, Blackstone EH, et al. Weighting Composite Endpoints in Clinical Trials: Evidence for the Heart Team. 2013;94(6):1908–13.

71. Anker SD, Schroeder S, Atar D, Bax JJ, Ceconi C, Cowie MR, et al. Traditional and new composite endpoints in heart failure clinical trials: facilitating comprehensive efficacy assessments and improving trial efficiency. Eur J Heart Fail. 2016 May;18(5):482–9.

72. Stammers a. N, Kehler DS, Afilalo J, Avery LJ, Bagshaw SM, Grocott HP, et al. Protocol for the PREHAB study--Pre-operative Rehabilitation for reduction of Hospitalization After coronary Bypass and valvular surgery: a randomised controlled trial. BMJ Open [Internet]. 2015;5(3):e007250.

73. Engelman DT, Ben Ali W, Williams JB, Perrault LP, Reddy VS, Arora RC, et al. Guidelines for Perioperative Care in Cardiac Surgery: Enhanced Recovery after Surgery Society Recommendations. JAMA Surg. 2019;12:1–12.

For peer review only

For peer review only - http://bmjopen.bmj.com/site/about/guidelines.xhtml

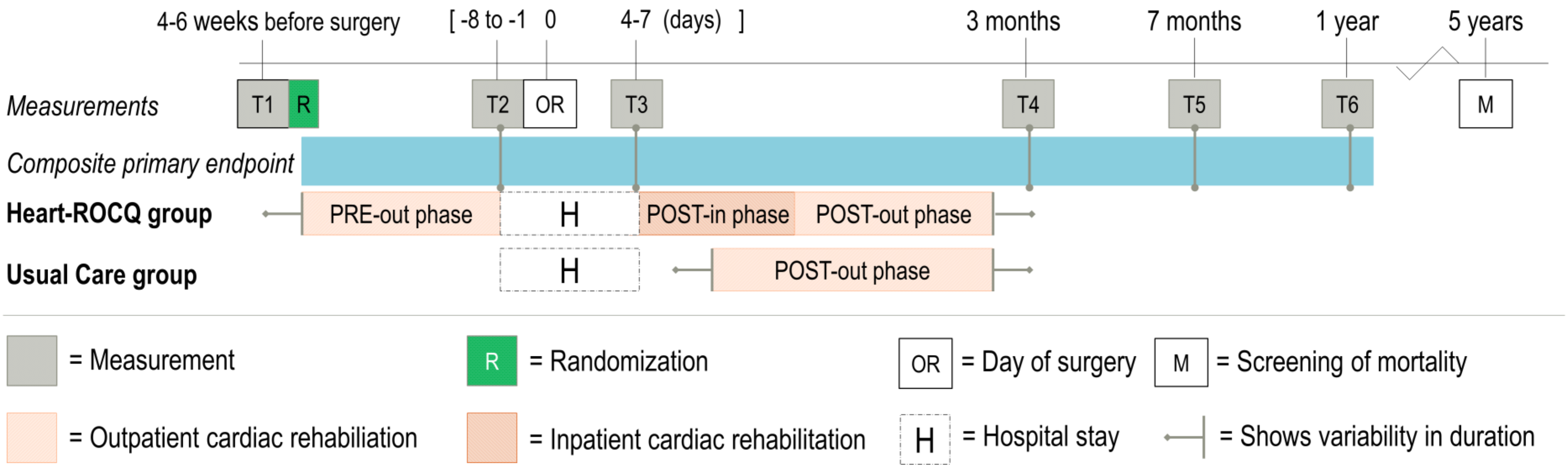

Figure 1: Research design of the Heart-ROCQ PROBE trial. The phases of both cardiac rehabilitation programs and the measurements are shown relative to the moment of surgery.

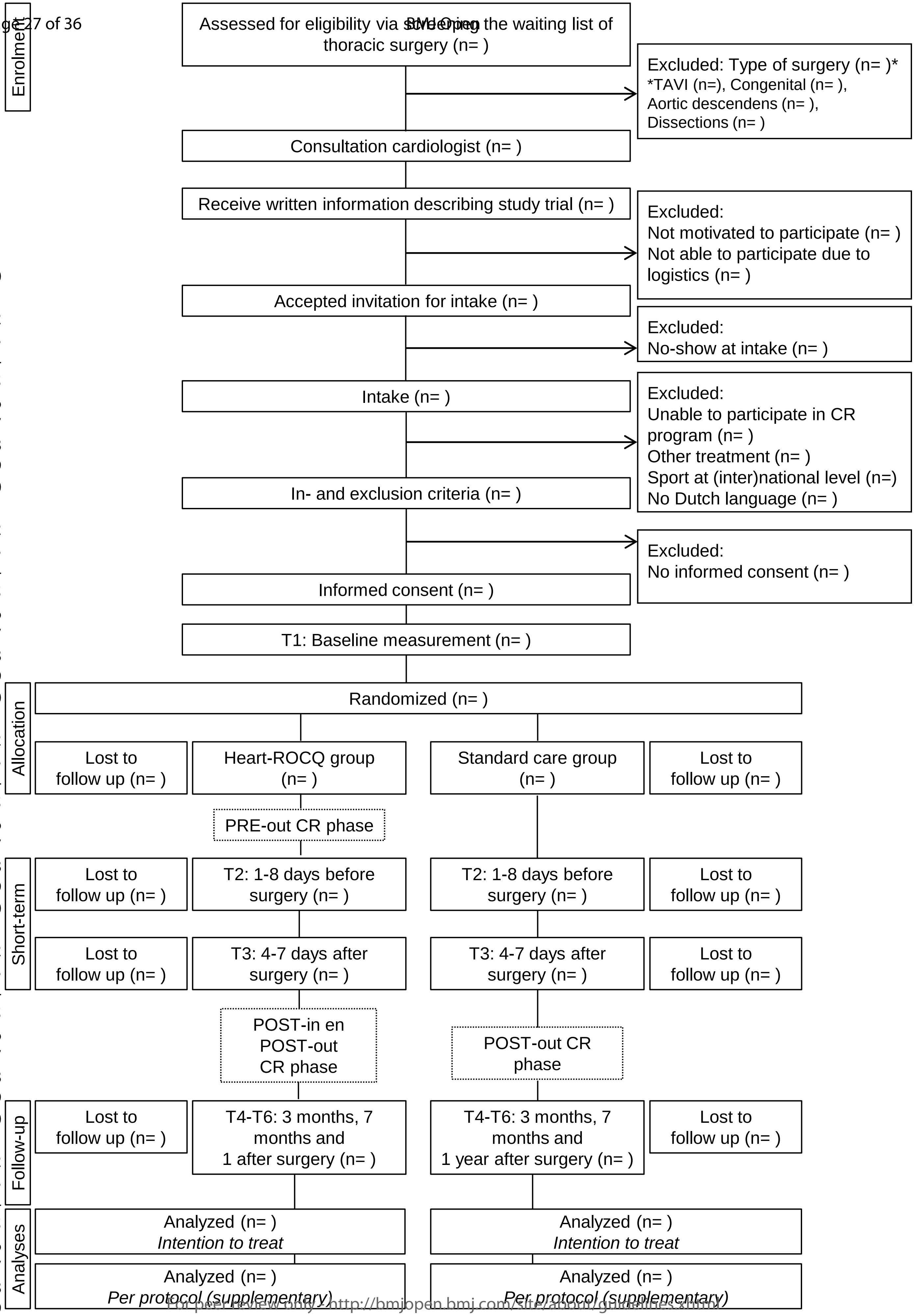
Enrolment
Assessed for eligibility via screening the waiting list of thoracic surgery (n=)
Excluded: Type of surgery (n=)*
*TAVI (n=), Congenital (n=), Aortic descendens (n=), Dissections (n=)
Consultation cardiologist (n=)
Receive written information describing study trial (n=)
Excluded:
Not motivated to participate (n=)
Not able to participate due to logistics (n=)
Accepted invitation for intake (n=)
Excluded:
No-show at intake (n=)
Intake (n=)
Excluded:
Unable to participate in CR program (n=)
Other treatment (n=)
Sport at (inter)national level (n=)
No Dutch language (n=)
In- and exclusion criteria (n=)
Excluded:
No informed consent (n=)
Informed consent (n=)
T1: Baseline measurement (n=)
Allocation
Randomized (n=)
Lost to follow up (n=)
Heart-ROCQ group (n=)
Standard care group (n=)
Lost to follow up (n=)
PRE-out CR phase
Short-term
Lost to follow up (n=)
T2: 1-8 days before surgery (n=)
T2: 1-8 days before surgery (n=)
Lost to follow up (n=)
Lost to follow up (n=)
T3: 4-7 days after surgery (n=)
T3: 4-7 days after surgery (n=)
Lost to follow up (n=)
POST-in en POST-out CR phase
POST-out CR phase
Follow-up
Lost to follow up (n=)
T4-T6: 3 months, 7 months and 1 after surgery (n=)
T4-T6: 3 months, 7 months and 1 year after surgery (n=)
Lost to follow up (n=)
Analyses
Analyzed (n=)
Intention to treat
Analyzed (n=)
Intention to treat
Analyzed (n=)
Per protocol (supplementary)
Analyzed (n=)
Per protocol (supplementary)

SUPPLEMENTARY INFORMATION - DESCRIPTION OF THE HEART-ROCQ PROGRAM

PRE-out phase – An outpatient cardiac rehabilitation phase during the waiting period
Three times per week, a minimum of three weeks

Physical therapy

Aims
- To maintain or improve patients physical capacity before surgery
- Patient learns to apply the stress-strain training principles
- To optimize pulmonary muscle strength
- Patient knows which breathing and coughing techniques should be used after surgery to prevent pulmonary complications

When needed:
- Patient learns to recognize body signals and boundaries.
- Patient is able to exercise despite of possible kinesiophobia

Type of exercise	Frequency	Intensity	Monitoring
IMT	3 × p / wk	- 6 cycles of 6 repetitions,<br>- rest periods of resp. 60, 45, 30, 15 and 5 s.<br>- 60-80% of maximal inspiratory pressure[65]	- Week 1: each training ↑ intensity with 10%<br>- Intensity ↑ with 5% if RPE <5[1]
Aerobic cycle	3 × p / wk	- 25 min. at RPE 3[1]<br>- Interval training will be given, if the patient is not able to perform endurance training.	- First training: 50% of POpeak<br>- Load ↑ if RPE< 3[1]<br>- Interval training: guided by complaints and RPE of 3
Resistance training	3 × p / wk	- 1-3 cycles of 10-15 repetitions<br>- Rest: 30-60 s.<br>- 50-80% of estimated 1RM<br>- On six fitness apparatus	- First training: 6-10 RM per fitness apparatus
Body awareness	1 × p / 2 wks	- 30 min. breathing and relaxation techniques	Not Applicable
Group education	Two sessions	- Basic training principles<br>- Forced expiration, huff- and cough techniques	Not Applicable

Dietary advice[2]

Aims
- To maintain or improve patients nutritional status
- Patient knows which nutrients are important focusing on the surgery
- Patients knows the importance of food in relation to cardiovascular disease and cardiovascular risk management

Type	Frequency	Content
Intake interview	One session	- Anamnesis about nutritional status
Counselling	On indication	- Individual sessions according to existing guidelines
Group education	One session	- 60 min., cardiovascular risk factors and dietary intake

Psychological guidance[2]

Aims
- To optimize mental status of the patients before surgery
- Patient has made a start with the awareness of cardiovascular risk factors

Intake interview	One session	- Anamnesis about mental status
Counselling	On indication	- Individual sessions according to evidence-based treatment protocols dependent on mental problems of patient[3]
Group education	One session	- Coping with psychological tension/stress, to process the mental trauma of cardiac surgery, and risk factors

No-smoking consultation *(For patients who smoke)*

Aim
- Patient gives up smoking during the waiting time before surgery

Intake interview & counselling	One session per wk	- 30 min., individual session based on existing guidelines

IMT: Inspiratory muscle training; RPE: Rate perceives exertion; POpeak: Maximum power output achieved during submaximal Ergometry test. [1]On a Borgscale 0-10; [2]Involvement of partner/relatives during group and individual sessions; [3] like cognitive behavioural therapy, eye movement desensitization and reprocessing (EMDR), acceptance and commitment therapy etc.

For peer review only - http://bmjopen.bmj.com/site/about/guidelines.xhtml

POST-in phase – An inpatient cardiac rehabilitation phase
Starting 4-7 days after surgery, duration of three weeks, weekends at home

Physical therapy

Aim

- To recover patients physical capacity
- Patient performs breathing and coughing techniques to prevent pulmonary complications
- Patient mobilize and can perform activities of daily living independently
- Patient knows about risk factors according to physical activity, knows the ACSM recommendations (moderate-intensity cardiorespiratory exercise training for ≥30min/day ≥5 days/week or vigorous-intensity cardiorespiratory exercise training for ≥20 min/day on ≥3 days/week or a combination[66]) and makes a plan to apply the ACSM recommendations in his own life
- Patient works on personal goals

Type of exercise	Frequency	Intensity	Monitoring
Individual therapy	2 × p / day	- Practice at transfers, walking, and climbing stairs<br>- Very light mobilizing exercises for upper extremity	- During the first 2 days of this phase.<br>- Extended, if patient is not able to participate in the group sessions.
Individual therapy	2 × p / wk	- Attention to personal goals	- Week 2 and 3
IMT[2]	3 × p / wk: 2 × under supervision, 1 × without supervision	- 6 cycles of 6 repetitions,<br>- rest periods of resp. 60, 45, 30, 15 and 5 s.<br>- intensity of 60-80% of maximum inspiratory pressure[65]	- First training: 50% of resistance of last preoperative training<br>- Intensity ↑ with 5% if RPE <5[1]<br>- IMT stops when resistance of preoperative training is reached
Aerobic cycle[2]	1 × p / day	- Week 1: duration of 5-20 min. at light intensity (RPE 2[1])<br>- Week 2 and 3: Work up to 25 min. at moderate intensity (RPE 3[1])<br>- Interval training will be given, if the patient is not able to perform endurance training.	- First training: at 50% of power output of last preoperative training<br>- Load ↑ if RPE< 2 à 3[1]<br>- Interval training: guided by complaints and RPE of 3.
Resistance training[2]	3-4 × p / wk	- 3 cycles of 15-20 repetitions<br>- Rest periods of 30-60 seconds<br>- On 6 fitness apparatus	- First training: 50% of resistance of last preoperative training for LE and 25% for UE.<br>- Gradual build up to 50-80% van 1RM based on RPE 3[1]
Body awareness[2]	1 × p / wk	- 30 min. breathing and relaxation techniques	Not Applicable

Dietary advice[3]

Aim

- To maintain or improve patients nutritional status
- Patient knows which nutrients are important focusing on the recovery of surgery
- Patients knows the importance of food in relation to cardiovascular disease and cardiovascular risk management

Type	Frequency	Content
Intake interview	One session	- Anamnesis about nutritional status post-surgery
Counselling	Sessions on indication	- Treatment according to existing guidelines
Group education	One session	- 90 min., skills needed to maintain a healthy life style

Psychological guidance[3]

Aim

- Patient start to process the mental trauma of cardiac surgery and the consequences of it
- Patient has self-management competence to maintain a healthy life style
- Patient and partner/relatives are able to support each other in the processing process

Intake interview	One session	- Anamnesis about mental status post-surgery
Intake interview & counselling	Sessions on indication	- Individual sessions according to evidence-based treatment protocols dependent on mental problems of patient[4]
Group education	One session	- 90 min., processing the cardiac surgery: possible reactions en consequences, coping with stress and healthy life style

IMT: Inspiratory muscle training; RPE: Rate perceives exertion; LE: Lower extremities; UE: Upper extremities. [1]On a Borgscale 0-10; [2]Group sessions start from day three of the inpatient phase or when patient is able to perform in the group activities; [3]Involvement of partner/relatives during group and individual sessions; [4] like cognitive behavioural therapy, eye movement desensitization and reprocessing (EMDR), acceptance and commitment therapy etc.

For peer review only

For peer review only - http://bmjopen.bmj.com/site/about/guidelines.xhtml

**POST-in phase – An inpatient cardiac rehabilitation phase (continuation)**<br>*Starting 4-7 days after surgery, duration of three weeks, weekends at home*		
**No-smoking consultation**[1] *(For patients who smoke)*		
Aim<br>- Patient is still motivated to stop smoking or patient is motivated to give up smoking (when patient did not give up smoking before cardiac surgery.		
Intake interview & counseling	1 × p / wk	- 30 min., individual sessions based on
Group education	One session	- 60 min., general information about smoking addiction and support from fellow smokers
**Return to work consultation**[1] *(for patients who are employed)*		
Aim<br>- Patient is informed about laws and regulation for illness, social security contributions, and medical examinations<br>- Patient knows their rights and obligations, the rights and obligations of the employer, the working conditions agency, the employee insurance agency, and re-integration companies<br>- Patient received tools to return adequately back to work (knows positive and negative factors that can impact the re-integration)		
Group education	One session	- 60 min., laws and regulation for illness, procedures, roles, rights and obligations of different involved persons, communication to involved persons (e.g. colleagues, employer), working during rehabilitation, positive and negative factors regarding to return to work
Counselling	Sessions on indication	- Individual sessions with labour consultant dependent on problems of patient

[1]Involvement of partner/relatives during group and individual sessions.

For peer review only

POST-out phase – An outpatient cardiac rehabilitation phase
Starting on Tuesday after discharge of the POST-in phase, two times per week, four weeks

Physical therapy

Aim

- Patient has optimized his/her physical capacity
- Patient knows his/her boundaries and limitations
- Patient knows about cardiovascular risk factors according to physical activity, knows the ACSM recommendations (moderate-intensity cardiorespiratory exercise training for ≥30min/day ≥5 days/week or vigorous-intensity cardiorespiratory exercise training for ≥20 min/day on ≥3 days/week or a combination[65]) and makes a plan to apply the ACSM recommendations in his own life
- Patient resumes his/her work or hobbies
- Patient experiences pleasure during exercise
- Patients achieves their personal goals

Type of exercise	Frequency	Intensity	Monitoring
Aerobic cycle	2 × p / wk	Depending on trainability of patient<br>- 25 min. at 50-80% HRR or RPE 5[1]	- Work up to 25 minutes at moderate intensity (RPE 3[1]), when patient was not able to do it in POST-in phase<br>- Load ↑ if RPE< 5[1] or HRR <50-80%
Resistance training	2 × p / wk	UE:<br>- 3-4 cycles of 10-15 repetitions<br>- Rest: 30-60 s.<br>- 50-80% of estimated 1RM<br>- On six fitness apparatus<br>LE:<br>- Focus on mobilization in week 1 & 2<br>- Focus on strength and endurance in week 3 & 4	- UE: Load ↑ if RPE< 5[1]<br>- LE: Under guidance of complaints
Sport and games	1 × p / wk	Focus on:<br>- Experiencing pleasure during exercise, regaining trust and handling boundaries.<br>- Exploring different types of sports and knowing the possibilities after CR	
Swimming	1 × p / wk		
Education	1 × p / wk	- Training principles of POST-out phase and a repetition of ACSM recommendations<br>- Awareness of exercise after CR; making a plan to exercise after CR<br>- Explanation of the results of the exercise test<br>- Discussing the plan to exercise, share experiences to expand exercise in home situation	

Dietary advice, psychological guidance, no-smoking consultation, and return to work consultation

Individual sessions are continued when aims are not achieved

RPE: Rate perceives exertion; HRR: Heart rate reserve; LE: Lower extremities; UE: Upper extremities; ACSM: American College of Sports Medicine; CR: Cardiac Rehabilitation. [1]On a Borgscale 0-10; [2]Involvement of partner/relatives during group and individual sessions.

For peer review only

For peer review only - http://bmjopen.bmj.com/site/about/guidelines.xhtml

SPIRIT
STANDARD PROTOCOL ITEMS: RECOMMENDATIONS FOR INTERVENTIONAL TRIALS

SPIRIT 2013 Checklist: Recommended items to address in a clinical trial protocol and related documents*

Section/item	Item No	Description	Addressed on page number
**Administrative information**			
Title	1	Descriptive title identifying the study design, population, interventions, and, if applicable, trial acronym	______1_______
Trial registration	2a	Trial identifier and registry name. If not yet registered, name of intended registry	______3, 6_____
	2b	All items from the World Health Organization Trial Registration Data Set	______3, 6_____
Protocol version	3	Date and version identifier	________6______
Funding	4	Sources and types of financial, material, and other support	______7_______
Roles and responsibilities	5a	Names, affiliations, and roles of protocol contributors	______1, 19___
	5b	Name and contact information for the trial sponsor	______1_______
	5c	Role of study sponsor and funders, if any, in study design; collection, management, analysis, and interpretation of data; writing of the report; and the decision to submit the report for publication, including whether they will have ultimate authority over any of these activities	______7_______
	5d	Composition, roles, and responsibilities of the coordinating centre, steering committee, endpoint adjudication committee, data management team, and other individuals or groups overseeing the trial, if applicable (see Item 21a for data monitoring committee)	______7, 8, 10, 13

For peer review only

Introduction

Background and rationale	6a	Description of research question and justification for undertaking the trial, including summary of relevant studies (published and unpublished) examining benefits and harms for each intervention	___4, 5______
	6b	Explanation for choice of comparators	___4, 5_______
Objectives	7	Specific objectives or hypotheses	___5_________
Trial design	8	Description of trial design including type of trial (eg, parallel group, crossover, factorial, single group), allocation ratio, and framework (eg, superiority, equivalence, noninferiority, exploratory)	___6, 7, ______

Methods: Participants, interventions, and outcomes

Study setting	9	Description of study settings (eg, community clinic, academic hospital) and list of countries where data will be collected. Reference to where list of study sites can be obtained	___6________
Eligibility criteria	10	Inclusion and exclusion criteria for participants. If applicable, eligibility criteria for study centres and individuals who will perform the interventions (eg, surgeons, psychotherapists)	___7________
Interventions	11a	Interventions for each group with sufficient detail to allow replication, including how and when they will be administered	___9________
	11b	Criteria for discontinuing or modifying allocated interventions for a given trial participant (eg, drug dose change in response to harms, participant request, or improving/worsening disease)	___NA______
	11c	Strategies to improve adherence to intervention protocols, and any procedures for monitoring adherence (eg, drug tablet return, laboratory tests)	___13______
	11d	Relevant concomitant care and interventions that are permitted or prohibited during the trial	___7_______
Outcomes	12	Primary, secondary, and other outcomes, including the specific measurement variable (eg, systolic blood pressure), analysis metric (eg, change from baseline, final value, time to event), method of aggregation (eg, median, proportion), and time point for each outcome. Explanation of the clinical relevance of chosen efficacy and harm outcomes is strongly recommended	___10-14___
Participant timeline	13	Time schedule of enrolment, interventions (including any run-ins and washouts), assessments, and visits for participants. A schematic diagram is highly recommended (see Figure)	____6-14___

For peer review only - http://bmjopen.bmj.com/site/about/guidelines.xhtml

Sample size	14	Estimated number of participants needed to achieve study objectives and how it was determined, including clinical and statistical assumptions supporting any sample size calculations	___15_______
Recruitment	15	Strategies for achieving adequate participant enrolment to reach target sample size	___7, 8, 16___

Methods: Assignment of interventions (for controlled trials)

Allocation:

Sequence generation	16a	Method of generating the allocation sequence (eg, computer-generated random numbers), and list of any factors for stratification. To reduce predictability of a random sequence, details of any planned restriction (eg, blocking) should be provided in a separate document that is unavailable to those who enrol participants or assign interventions	___7, 8______
Allocation concealment mechanism	16b	Mechanism of implementing the allocation sequence (eg, central telephone; sequentially numbered, opaque, sealed envelopes), describing any steps to conceal the sequence until interventions are assigned	___7, 8______
Implementation	16c	Who will generate the allocation sequence, who will enrol participants, and who will assign participants to interventions	____7, 8_____
Blinding (masking)	17a	Who will be blinded after assignment to interventions (eg, trial participants, care providers, outcome assessors, data analysts), and how	____7, 8_____
	17b	If blinded, circumstances under which unblinding is permissible, and procedure for revealing a participant's allocated intervention during the trial	___NA________

Methods: Data collection, management, and analysis

Data collection methods	18a	Plans for assessment and collection of outcome, baseline, and other trial data, including any related processes to promote data quality (eg, duplicate measurements, training of assessors) and a description of study instruments (eg, questionnaires, laboratory tests) along with their reliability and validity, if known. Reference to where data collection forms can be found, if not in the protocol	__11-14_______
	18b	Plans to promote participant retention and complete follow-up, including list of any outcome data to be collected for participants who discontinue or deviate from intervention protocols	__NA_________

Data management	19	Plans for data entry, coding, security, and storage, including any related processes to promote data quality (eg, double data entry; range checks for data values). Reference to where details of data management procedures can be found, if not in the protocol	__13_________
Statistical methods	20a	Statistical methods for analysing primary and secondary outcomes. Reference to where other details of the statistical analysis plan can be found, if not in the protocol	___15, 16_____
	20b	Methods for any additional analyses (eg, subgroup and adjusted analyses)	___15________
	20c	Definition of analysis population relating to protocol non-adherence (eg, as randomised analysis), and any statistical methods to handle missing data (eg, multiple imputation)	_____15_______
**Methods: Monitoring**			
Data monitoring	21a	Composition of data monitoring committee (DMC); summary of its role and reporting structure; statement of whether it is independent from the sponsor and competing interests; and reference to where further details about its charter can be found, if not in the protocol. Alternatively, an explanation of why a DMC is not needed	___13________
	21b	Description of any interim analyses and stopping guidelines, including who will have access to these interim results and make the final decision to terminate the trial	___15________
Harms	22	Plans for collecting, assessing, reporting, and managing solicited and spontaneously reported adverse events and other unintended effects of trial interventions or trial conduct	___13________
Auditing	23	Frequency and procedures for auditing trial conduct, if any, and whether the process will be independent from investigators and the sponsor	___13________
**Ethics and dissemination**			
Research ethics approval	24	Plans for seeking research ethics committee/institutional review board (REC/IRB) approval	___6________
Protocol amendments	25	Plans for communicating important protocol modifications (eg, changes to eligibility criteria, outcomes, analyses) to relevant parties (eg, investigators, REC/IRBs, trial participants, trial registries, journals, regulators)	___6_________

Consent or assent	26a	Who will obtain informed consent or assent from potential trial participants or authorised surrogates, and how (see Item 32)	__7, 8_______
	26b	Additional consent provisions for collection and use of participant data and biological specimens in ancillary studies, if applicable	__7, 8_______
Confidentiality	27	How personal information about potential and enrolled participants will be collected, shared, and maintained in order to protect confidentiality before, during, and after the trial	__13_________
Declaration of interests	28	Financial and other competing interests for principal investigators for the overall trial and each study site	__19_________
Access to data	29	Statement of who will have access to the final trial dataset, and disclosure of contractual agreements that limit such access for investigators	__13_________
Ancillary and post-trial care	30	Provisions, if any, for ancillary and post-trial care, and for compensation to those who suffer harm from trial participation	__13_________
Dissemination policy	31a	Plans for investigators and sponsor to communicate trial results to participants, healthcare professionals, the public, and other relevant groups (eg, via publication, reporting in results databases, or other data sharing arrangements), including any publication restrictions	___6_________
	31b	Authorship eligibility guidelines and any intended use of professional writers	___6_________
	31c	Plans, if any, for granting public access to the full protocol, participant-level dataset, and statistical code	___NA_______
**Appendices**			
Informed consent materials	32	Model consent form and other related documentation given to participants and authorised surrogates	___13________
Biological specimens	33	Plans for collection, laboratory evaluation, and storage of biological specimens for genetic or molecular analysis in the current trial and for future use in ancillary studies, if applicable	___NA________

*It is strongly recommended that this checklist be read in conjunction with the SPIRIT 2013 Explanation & Elaboration for important clarification on the items. Amendments to the protocol should be tracked and dated. The SPIRIT checklist is copyrighted by the SPIRIT Group under the Creative Commons "Attribution-NonCommercial-NoDerivs 3.0 Unported" license.

BMJ Open

HEART Rehabilitation in patients awaiting Open-heart surgery targeting to prevent Complications and to improve Quality of life (Heart-ROCQ): Study protocol for a Prospective Randomised Open Blinded End-point (PROBE) trial

Journal:	*BMJ Open*
Manuscript ID	bmjopen-2019-031738.R2
Article Type:	Protocol
Date Submitted by the Author:	15-Aug-2019
Complete List of Authors:	Hartog, Johanneke; UMCG, Cardio-thoracic Surgery<br>Blokzijl, Fredrike; UMCG, Cardio-thoracic Surgery<br>Dijkstra, Sandra; UMCG, Cardio-thoracic Surgery<br>DeJongste, Mike; UMCG<br>Reneman, Michiel; UMCG, Rehabilitation Medicine<br>Dieperink, Willem; UMCG, Critical Care<br>van der Horst, Iwan; Maastricht University Medical Centre+, Intensive Care<br>Fleer, Joke; UMCG, Health Psychology<br>van der Woude, Lucas; UMCG, Center for Human Movement Sciences; UMCG, Rehabilitation Medicine<br>Van der Harst, Pim; UMCG, Cardiology<br>Mariani, Massimo; UMCG, Cardio-thoracic surgery
**Primary Subject Heading**:	Cardiovascular medicine
Secondary Subject Heading:	Cardiovascular medicine, Health economics, Mental health, Rehabilitation medicine, Surgery
Keywords:	rehabilitation, cardiac surgical procedures, life style, health status, postoperative complications, cost-benefit analysis

SCHOLARONE™
Manuscripts

Trial design BMJ Open

Title

HEART Rehabilitation in patients awaiting **O**pen-heart surgery targeting to prevent **C**omplications and to improve **Q**uality of life (Heart-ROCQ) - Study protocol for a **P**rospective **R**andomised **O**pen **B**linded **E**nd-point (PROBE) trial

Authors

Johanneke Hartog, MSc[a]; Fredrike Blokzijl, MSc[a]; Sandra Dijkstra, MSc[a]; Mike J.L. DeJongste, M.D., PhD, FESC[b]; Michiel F. Reneman, PhD[c]; Willem Dieperink, PhD[d]; Iwan C.C. van der Horst, M.D., PhD[b,e]; Joke Fleer, PhD[f]; Lucas H.V. van der Woude, PhD[c,g]; Pim van der Harst, M.D., PhD[h]; Massimo A. Mariani, M.D., PhD[a]

a University of Groningen, University Medical Centre Groningen, Department of Cardio-thoracic Surgery, Groningen, The Netherlands

b University of Groningen, University Medical Centre Groningen, Groningen, The Netherlands

c University of Groningen, University Medical Centre Groningen, Department of Rehabilitation Medicine, Groningen, The Netherlands

d University of Groningen, University Medical Centre Groningen, Department of Critical Care, Groningen, The Netherlands

e Maastricht University Medical Centre, Department of Intensive Care, Maastricht, The Netherlands.

f University of Groningen, University Medical Centre Groningen, Department of Health Psychology, Groningen, The Netherlands

g University of Groningen, University Medical Centre Groningen, Centre for Human Movement Sciences, Groningen, The Netherlands

h University of Groningen, University Medical Centre Groningen, Department of Cardiology, Groningen, The Netherlands

Corresponding Author:

For peer review only

Johanneke Hartog, Department of Cardio-thoracic Surgery, University Medical Centre Groningen (UMCG), Hanzeplein 1, AB41, 9713 GZ Groningen, The Netherlands

Phone: +3150 3617527; Email: j.hartog@umcg.nl; FAX: +31503614391

For peer review only

For peer review only - http://bmjopen.bmj.com/site/about/guidelines.xhtml

ABSTRACT

Introduction

The rising prevalence of modifiable risk factors (e.g. obesity, hypertension, and physical inactivity) is causing an increase in possible avoidable complications in patients undergoing cardiac surgery. This study aims to assess whether a combined pre- and postoperative multidisciplinary cardiac rehabilitation (CR) program (Heart-ROCQ program) can improve functional status and reduce surgical complications, readmissions and major adverse cardiac events (MACE) as compared to standard care.

Methods and analysis

Patients (n=350) are randomised to the Heart-ROCQ program or standard care. The Heart-ROCQ program consists of a preoperative optimization phase whilst waiting for surgery (3 times p/week, minimum 3 weeks), a post-operative inpatient phase (3 weeks) and an outpatient CR phase (2 times p/week, 4 weeks). Patients receive multidisciplinary treatment (e.g. physical therapy, dietary advice, psychological sessions, and smoking cessation). Standard care consists of 6 weeks post-surgery outpatient CR with education and physical therapy (2 times p/week). The primary outcome is a composite weighted score of functional status, surgical complications, readmissions, and MACE and is evaluated by a blinded end-point committee. Secondary outcomes are length of stay, physical, and psychological functioning, life style risk factors, and work participation. Finally, an economic evaluation is performed. Data is collected at six time points: at baseline (start of the waiting period), the day before surgery, at discharge from the hospital, and at 3, 7, and 12 months post-surgery.

Ethics and dissemination

This study will be conducted according to the principles of the Declaration of Helsinki (version 8, October 2013). The protocol has been approved by the Medical Ethical Review Board (METc) of the UMCG (No. 2016/464). Results of this study will be submitted to a peer-reviewed scientific journal and can be presented at national and international conferences.

For peer review only - http://bmjopen.bmj.com/site/about/guidelines.xhtml

Registration: The Heart-ROCQ study has been registered at ClinicalTrials.gov (No. NCT02984449).

Data sharing statement: Data are available upon reasonable request after the results have been published.

Strength and limitations of this study

- A prospective randomised open, blinded end-point (PROBE) trial
- Comparison of a unique multidisciplinary pre- and post-operative CR program with a standard care post-operative outpatient CR program
- Patient-oriented: primary and secondary outcomes provide a comprehensive and clinically relevant evaluation of patients' recovery from surgery (e.g. on physical, psychological, social domains)
- Both short- and long term effects of the CR programs are included
- Single centre study, albeit that patients are referred from four hospitals

For peer review only

INTRODUCTION

The leading cause of death in Western countries is ischemic heart disease.(1) One of the treatments for severe ischemic heart disease is cardiac surgery. The risk of post-operative complications related to cardiac surgery is substantial; pulmonary complications (up to 33%), delirium (~26%), and arrhythmias (~30%) have been reported to occur.(2–4) These complications are associated with prolonged hospitalisation, increased adverse events (i.e. readmission, stroke, myocardial infarction, and mortality), reduced health related quality of life (HRQoL), and higher health care costs.(5–10) Patients with poor dietary habits (present in ~80% of the candidates for cardiac surgery), who are physically inactive (~45%), who smoke (22%), or who experience depression and/or anxiety disorders (~30%) are at higher risk for post-operative complications and are at risk for lack of functional benefits after cardiac surgery.(8,11–19)

Over the last decades risk factors such as age, obesity, diabetes, hypertension and dyslipidemia have steadily increased in patients undergoing cardiac surgery.(20,21) Despite their adverse effects on treatment outcomes, reducing the burden of modifiable risk factors are currently not part of standard clinical care before and after cardiac surgery. Before cardiac surgery, patients often have a preoperative period of several weeks on the waiting list in which they receive little or no guidance. This waiting period has been associated with increased psychological stress, feelings of anxiety and reduced functional status.(22,23) With respect to inactivity during hospitalisation after cardiac surgery, research has shown that during the 8 to 11 days of hospitalisation, patients spent the majority of their time sitting or in a supine position.(24,25) In-hospital physical inactivity is a predictor of a longer hospital stay and re-hospitalisation.(24,26,27) It causes a decrease in muscle strength and aerobic capacity, both fundamental in the performance of activities of daily living.(28,29) This reduced physical capacity may seriously impact independence, especially since these patients are often elderly and the functioning of their entire physiological system is already reduced.(30)

The goal of Cardiac rehabilitation (CR) is to improve the pre- and post-operative status of patients undergoing cardiac surgery. CR aims "to favourably influence the underlying cause of cardiovascular disease, as well as to provide the best possible physical, mental, and social

For peer review only - http://bmjopen.bmj.com/site/about/guidelines.xhtml

conditions".(31) Post-operative CR is already an essential part of standard care in the Netherlands, although many hospitals in the Netherlands provide a phase II outpatient CR program, starting 3 to 6 weeks after cardiac surgery.(32,33) Benefits of post-operative CR are reported for a variety of cardiac patients,(34) however the evidence in patients undergoing cardiac surgery is still lacking with regard to patient-relevant outcomes and mortality.(35) In addition to post-operative CR, small trials suggested that preoperative CR is effective in reducing post-operative pulmonary complications, duration of hospital stay, improving HRQoL, physical fitness and increasing the compliance to post-operative CR.(23,36,37) However, long-term effects and the effects on other complications remain unclear. Furthermore, most studies investigated the effect of preoperative CR *or* post-operative CR, but not the effect of both CR methods combined. The hypothesis is that a combined pre- and post-operative CR program is more beneficial when compared to a separate preoperative CR program *or* a single post-operative CR program.

The aim of the Heart-ROCQ study is to determine the effect of a comprehensive pre- and post-operative CR program (the Heart-ROCQ program) on functional status, post-operative surgical complications, readmissions to hospital, and major adverse cardiac events (MACE) compared to a regular Dutch post-operative outpatient CR program (the Standard Care CR program).(32) In addition, the effect of the Heart-ROCQ program on physical and psychological functioning, including HRQoL, life style risk factors, work participation, and cost-effectiveness are evaluated in comparison to the Standard Care CR program. To assess who will benefit from CR and why the CR programs are effective, moderator and mediator analyses are performed.

For peer review only - http://bmjopen.bmj.com/site/about/guidelines.xhtml

METHODS AND ANALYSIS

Study design and organization

This investigator-initiated prospective randomised open, blinded end-point (PROBE) trial is executed in a single Centre (University Medical Centre Groningen [UMCG]). Patients from three referral hospitals (Ommelander Hospital Groningen [OZG], Martini Hospital Groningen [MZH], and Wilhelmina Hospital Assen [WZA]) who are accepted for cardiac surgery at the UMCG are also approached to participate. Patients are randomly assigned to a combined pre- and post-operative multidisciplinary CR program (the Heart-ROCQ group) or a regular phase II outpatient CR program after surgery (the Standard Care group).(32) Figure 1 provides an overview of the study design.

Ethical considerations and dissemination

This study is conducted according to the principles of the Declaration of Helsinki (version 8, October 2013) and according to the research code of the UMCG. It has been registered at ClinicalTrials.gov (No. NCT02984449) and the protocol (version 2, 2016-Dec-19) has been approved by the Medical Ethical Review Board (METc) of the UMCG (No. 2016/464). If applicable, substantial amendments will be notified to the METc for approval and changes will be written into articles describing the results of the study. Results of this study will be submitted to a peer-reviewed scientific journal and can be presented at national and international conferences.

For peer review only

Funding

The Heart-ROCQ study is financially supported by Edwards Lifesciences SA, Abbott (former St. Jude Medical Nederland B.V.) and 'Stichting Beatrixoord Noord-Nederland'. The sponsors are not involved in the design or execution of the study.

Participants

Patients (≥ 18 years) admitted to elective coronary artery bypass grafting (CABG), valve surgery, aortic surgery, or combined procedures are eligible. Patients accepted for congenital procedures,

For peer review only - http://bmjopen.bmj.com/site/about/guidelines.xhtml

transcatheter aortic valve implantations (TAVI), aortic dissections, or aortic descending repair are excluded. Other exclusion criteria are: inability to participate in all program elements of the Heart-ROCQ program due to disorders of the nervous or musculoskeletal system that limits exercise capacity, chronic obstructive pulmonary disease (COPD) with Global Initiative for Chronic Obstructive Lung Disease (GOLD) criteria classification 3 or 4,(38) addiction to alcohol or drugs, a serious psychiatric illness (i.e. recently experienced psychosis, bipolar disorder, diagnosis of schizophrenia, serious cognitive, or neurological problems, and acute suicidal ideations or behaviour), or when it is undesirable to exercise (i.e. hypertrophic cardiomyopathy, unstable angina, advice from cardiologist); any treatment which is planned during one of the CR programs and which is expected to interrupt attendance to the CR program (e.g. planned organ transplantation, preoperative endocarditis, or planned chemotherapy etc.); playing a sport at (inter)national level; being unable to read, write, or understand Dutch.

Study enrolment, randomization and registry

Figure 2 shows the flow chart of the Heart-ROCQ study. Patients on the waiting list of the thoracic surgery department and meeting the study criteria for type of surgery are asked to participate by their cardiologist. The cardiologist provides the patients with study information and an invitation to meet the researcher at the preoperative consultation. At the preoperative consultation, the researcher will obtain informed consent and conduct the baseline measurements. Eligible patients who have signed informed consent and performed the baseline measurements, are randomised to the Heart-ROCQ group or Standard Care group. Randomization (concealed group allocation in REDCap, random blocks of 2-4, 1:1 ratio) is stratified for weight of the surgery (isolated CABG, single non-CABG [i.e. replacement or repair of part of aortic or valve], 2 procedures or 3 procedures), gender, and age (≥65 and <65 years). Prior to the start of the study, the randomization lists were created (using the 'ralloc' function of Stata/SE, version 13.0) and imported into the secure data collection tool REDCap (version 7.3.2), by an independent researcher. Medical staff and researchers are not blinded to group allocation, because of logistic reasons. The primary end point is evaluated by an independent end-point committee, blinded for group allocation.

For peer review only - http://bmjopen.bmj.com/site/about/guidelines.xhtml

Patients who are not willing to participate are asked to give written consent for using data that are collected during routine care. The data are collected in the Heart-ROCQ study registry to increase insight into-potential differences between patients who participated in the study and patients who did not. The Heart-ROCQ registry will thus provide more insight into the generalizability of the results. Data from this registry are not used for the primary statistical analyses.

Intervention

Heart-ROCQ group

The Heart-ROCQ group receives a CR program at the Centre of Rehabilitation of the UMCG (location Beatrixoord, which is located 6 km from the hospital of the UMCG) consisting of 3 phases. The first phase is an outpatient preoperative optimization phase during the waiting period (3 times per week, minimum of 3 weeks). The second phase is a post-operative inpatient CR phase (3 weeks, weekdays only) followed by the third phase, an outpatient CR phase (2 times per week, for 4 weeks). During each phase, all participants receive physical therapy including group sessions of inspiratory muscle training (IMT),(39) strength training, aerobic cycling, breathing, coughing and relaxation. In addition, patients have an assessment with a dietician and a psychologist and take part, when indicated, in individual sessions to optimize their health. In addition, different group education sessions are organized regarding coping with stress, awareness of risk factors, and maintaining a healthy life style. Two additional modules, namely coaching to stop smoking and to return to work, are available for patients who respectively smoke or are employed. A detailed description of the CR program is given in the supplementary information.

Standard Care group

In the Netherlands, the current standard of care consists of a phase II outpatient CR program, which is conducted at the referred hospital.(32,33) In general, this CR program starts 3 to 6 weeks after discharge from the hospital and lasts for 6 weeks. The program consists of four educational sessions (regarding risk factors and retaining a healthy life style) and physical therapy (2 times per week: 30 min. cycling together with 30 min. sports and games, relaxation therapy, or strength training).

For peer review only - http://bmjopen.bmj.com/site/about/guidelines.xhtml

Furthermore, patients can be referred to a psychologist or a dietician when needed. The content of this CR program is based on the Dutch CR guidelines.(40)

Composite primary end point: functional status, complications and events

The primary outcome is a composite weighted score of functional status, post-operative surgical complications, readmissions to the hospital, and MACE. Each event is scored (ranging from 1 to 3 points), separately. Table 1 shows an overview of the primary outcome and the scoring system. The scores of all events are combined to calculate a total score. Only the most serious complication is counted per event (e.g. when a percutaneous coronary intervention [score 1] is complicated by a stroke [score 2], the score will be 2 and not 3 [1 + 2]).

The concept of the composite weighted score is adapted from the African-American Heart Failure Trial (A-HeFT).(41) Functional status is assessed through two health domains of the Medical Outcome Study 36-item General Health Survey (RAND-36 version 2)(42): physical functioning and physical health problems. The primary end point is evaluated at 3 months (T4) and 1 year (T6) after surgery (Figure 1). Deep wound infections and surgical re-explorations are screened up to 120 days, after surgery. Other post-operative surgical complications are measured in the period between the surgery and when a patient meets the UMCG discharge criteria (Table 2). Hospital admissions are checked between baseline and one year after surgery. To prevent bias (since the Heart-ROCQ group follows the in-patient CR phase after surgery) hospital admissions between the day of admission before surgery and 30 days after surgery are not included when determining the (calculated) primary end point.

Secondary outcomes

Complications and events

All individual components of the composite end point regarding the complications and events will be analysed separately as secondary outcomes. Table 3 summarizes the definitions of the secondary complications and events, including at the time of screening.

For peer review only

For peer review only - http://bmjopen.bmj.com/site/about/guidelines.xhtml

All documents concerning the composite primary end point and the secondary complications are encrypted and subsequently adjudicated by the independent end-point committee. The end-point committee consists of four members (cardiologists and cardiothoracic surgeons), who are not employed in the UMCG and are blinded for group allocation.

For peer review only

For peer review only - http://bmjopen.bmj.com/site/about/guidelines.xhtml

Table 1: definitions and score of the components of the composite primary end point

**Functional status**	**Score**
Worsening in physical functioning (*domain score of health related quality of life, rand36_v2)*†	1
No change or improvement in physical functioning†	0
Worsening in physical problem (*domain score of health related quality of life, rand36_v2)*†	1
No change or improvement in physical problem†	0
A clinical relevant worsening is classified as minimal change according to *Wyrwich et al., 2004*(43)	
**(Serious) Adverse Events**	
No serious adverse event	0
Prolonged mechanical ventilation<br>*Mechanical ventilation longer than 24 hours*	1
Lung infection<br>*1) A new lung radiographic infiltrate AND 2) two signs that the infiltrate is infectious origin (i.e. a) fever - body temperature >38°C or <36°C -, b) leukocytosis - white blood cells >10,000 or <4000, c) positive sputum culture AND/OR d) decline in oxygenation*	1
Delirium<br>*1) A DOS score ≥ 3 at hospital ward AND/OR 2) Diagnosis confirmed by a psychiatrist, geriatrist, or supervising specialist according to the DSM-IV criteria resulting in treatment with medication*	1
Readmissions to intensive care unit<br>*Unrelated to a secondary end point*	1
Deep wound infection<br>*Deeper tissues are affected (muscle, sternum, and mediastinum) and must include: 1) surgical drainage / re-fixation OR 2) an organism is isolated from culture of mediastina tissue or fluid, OR 3) antibiotic treatment, because of sternum wound*	2
Readmissions to hospital<br>*An unplanned hospital stay with different dates of admission and discharge with a medical indication (i.e. clinical signs and symptoms or change of treatment)*	1
Any cardiothoracic surgical interventions<br>*Graft- or valve failure, CABG, valve, aortic, or other cardiac surgery*‡	2
Any percutaneous interventions<br>*PCI, TAVI, etc.*	1
Myocardial infarction<br>*According to the third universal definition of myocardial infarction*§	2
Cerebral vascular accident / stroke<br>*Acute neurological event of at least 24 hours of duration, with focal signs and symptoms and without evidence supporting any alternative explanation. Diagnosis of stroke requires confirmation by CT, MRI, or pathological confirmation.*	2
Sudden death survivor<br>*The sudden onset of symptoms, such as chest pain and cardiac arrhythmias, ventricular tachycardia, which lead to the loss of consciousness and cardiac arrest followed by reanimation and does not lead to biological death.*	2
Death<br>*All-cause mortality*	3
Total score = *sum of physical status and SAE's at 3 months and 1 year (worst score of each event)*	

†Compared with baseline; ‡According to the definitions of the 'Begeleidingscommissie Hartinterventies Nederland; §Thygesen et al., 2012(44); DOS: Delirium Observation Screening scale(45); CABG: Coronary arterial bypass graft; PCI: Percutaneous coronary intervention; TAVI: Transcathetic aortic valve implantation; In gray: post-operative complications can be scored ones; SAE: Serious adverse event.

For peer review only - http://bmjopen.bmj.com/site/about/guidelines.xhtml

236 *Table 2: Discharge criteria of the University Medical Centre Groningen (UMCG)*

1) no drain, no external pacemaker lead, no infusion, or oxygen present,
2) stable clinical conditions (stable lab results, X-ray, and haemodynamic parameters),
3) able to perform basic activity of daily living (ADL)-activities (i.e. going independently to the toilet).

237

238 *Table 3: definitions of the secondary complications and events*

**Definitions**	**Time of measure**
Atrial fibrillation<br>*New onset of atrial fibrillation or atrial flutter requiring medical treatment or cardioversion*	Surgery to T3
Prolonged ICU stay<br>*When the number of calendar days is two or more from ICU admission to discharge*	Initial stay
Readmissions to hospital<br>*The number of unplanned hospital stays with different dates of admission and discharge with a medical indication (i.e. clinical signs and symptoms or change of treatment)*<br>*a) directly related to a cardiac cause*<br>*b) not directly related to a cardiac cause*	Baseline to T6§
Hospitalisation days<br>*Total number of days of hospitalisation*<br>*a) directly related to a cardiac cause*<br>*b) not directly related to a cardiac cause*	Baseline to T6§
Cardiovascular death<br>*Any death due to proximate cardiac or cardiovascular cause (e.g. Myocardial infarct, low-output failure, fatal arrhythmia, death secondary to a cerebral vascular accident, pulmonary embolism, ruptured or dissecting aortic aneurysm, or other vascular diseases), un-witnessed death, and death of unknown cause, and all procedure related deaths (e.g. PCI, CABG), including those related to concomitant treatment†.*	Baseline to 5 years after surgery
Non-cardiovascular death<br>*Any death not covered by above definition, such as death caused by infection, malignancy, sepsis, pulmonary causes, accident, suicide, or trauma*	Baseline to 5 years after surgery
**Concerning safety**	
Surgical re-exploration for bleeding/ tamponade<br>*Surgical incision into the sternum as a result of a bleeding or tamponade*<br>*a) acute: presented within 24 hours after surgery*<br>*b) late: presented after 24 hours after surgery*	Surgery to T4
Surgical re-exploration dehiscence<br>*Aseptic wound dehiscence*	Surgery to T4

239 †Specifically, any unexpected death even in patients with coexisting potentially fatal non-cardiac disease
240 (e.g. cancer, infection) will be classified as cardiac death; §Not measured between the day of admission
241 before surgery and 30 days after surgery, because of POST-in phase of Heart-ROCQ program; ICU:
242 Intensive care unit; T4: Follow-up at 3 months after surgery; T6: Follow-up at 12 months after surgery.

For peer review only - http://bmjopen.bmj.com/site/about/guidelines.xhtml

Questionnaires and physical tests

Table 4 shows the physical tests and questionnaires of the secondary outcomes regarding physical and psychological health, life style risk factors, and the economic evaluation. Physical tests and questionnaires are completed at six assessment points (Figure 1). The preoperative measurements (T1 and T2) are conducted at the start (preoperative consultation) and the end (1 to 8 days before surgery) of the waiting period. The third measurement (T3) is performed when patients meet the UMCG discharge criteria (Table 2). The follow-up measurements (T4, T5, and T6) are at 3, 7, and 12 months after surgery. Patients are asked to fill in questionnaires online or when requested on paper, prior to the visits for the physical tests. All adverse events reported spontaneously by the patient or observed by the investigator are recorded. In addition, serious adverse events are reported to the METc. Data are stored in REDCap and on a secure drive at the department CardioResearch in the UMCG. Two times per year, the study is monitored by a trained research monitor from another department of the UMCG. Details of procedures, data collection, management and monitoring can be found in the Trial Master File of the Heart-ROCQ study (can be obtained by the investigators). On the 12th of June 2018, the study was audited and certified for the ISO 9001: 2015 independently by DNV GL.

<u>Potential moderators</u>

Preoperative risk profile (i.e. Euroscore II, medical history, life style risk factors, physical, and mental status), surgery parameters, and demographics are collected form the medical record and baseline measurements. The content of the CR program is described in terms of compliance, duration of CR program, type of treatment, frequency, and training load (i.e. for bicycle training: external workload, heart rate response, rate of perceived exertion using the Borg scale, and for strength training: sets, repetitions, and intensity) for both the Heart-ROCQ group as the Standard Care group.

For peer review only - http://bmjopen.bmj.com/site/about/guidelines.xhtml

Table 4: Time of measure, physical tests, and questionnaires of the secondary outcomes

Secondary outcomes	Measure	Time of measure
Physical health		
Cardiorespiratory fitness	6MWT(46)	T1-T4, T6
Muscle strength	STS_10, grip & leg strength(47,48)	T1-T4, T6
Independence in ADL	KATZ(49,50)	T1, T4, T6
Psychological health		
General anxiety	GAD-7(51)	T1,T2, T4, T6
Feelings of depression	PHQ-9(52,53)	T1,T2, T4, T6
Health related Quality of Life	Rand-36_v2(42)	T1, T4, T6
Life style risk factors		
Physical activity	iPAQ(54) & Actigraph†(55)	T1, T4, T6
Obesity indices	BMI, waist-to-hip ratio	T1, T4, T6
Smoking behaviour	Number of cigarettes per day	T1, T4, T6
Economic evaluation		
Health care use & related medical costs	iMCQ(56)	T1, T4-T6
Work participation‡	iPCQ(57)	T1, T4-T6
QALYs	EQ-5D-5L(58)	T1, T4-T6
Potential mediators		
Cardiac self-efficacy	CSE(59)	T1, T2, T4, T6
Illness representations	IPQ-R(60)	T1, T2, T4, T6

†Patients are asked to wear the Actigraph during waking hours for 1 consecutive week; ‡Health-related productivity losses of paid work and unpaid work; T1: Begin of waiting time; T2: 1 to 8 days before surgery; T3: Moment that patients meet the UMCG discharge criteria; T4-T6: Follow-up at 3, 7, and 12 months after surgery; 6MWT: Six minutes walking test; STS_10: Ten times sit to stand test; ADL: Activities of Daily Living; KATZ: Katz Index of Independence in Activities of Daily Living; GAD-7: Generalized Anxiety Disorder 7-item scale; PHQ-9: Patient Health Questionnaire 9-item scale; iPAQ: International Physical Activity Questionnaire; BMI: Body Mass Index; iMCQ: iMTA Medical Cost Questionnaire; iPCQ: iMTA Productivity Cost Questionnaire; QALY: Quality-adjusted life years; EQ-5D-5L: EuroQol five-dimensional questionnaire; CSE: Cardiac Self-efficacy; IPQ-R: Illness Perception Questionnaire, Revised.

Potential mediators

Illness perceptions (i.e. patients mental representations of their illness based on different sources of information) are measured with three subscales (personal control, treatment control, and consequences) of the revised illness perception questionnaire (IPQ-R).(60) These subscales are chosen because of their sensitivity to change and because of their relation to psychological distress(61). The cardiac self-efficacy (CSE) scale(59) is used to measure CSE (i.e. patients confidence to perform a specific task related to cardiac disease).

For peer review only

For peer review only - http://bmjopen.bmj.com/site/about/guidelines.xhtml

Statistical analyses

Sample size

Assuming a normal distribution, the mean weighted score of the primary end point is estimated on 1.0 with a standard deviation of 0.9 at 1 year after surgery. This estimation is based on historical data of the UMCG, an unpublished on-going pilot study in the UMCG, the Dutch registration database,(62) and data reported in literature.(6,63–65) A decrease of 0.3 is expected in the Heart-ROCQ group, based on previous studies comparing CR with standard care(36,64,66) (i.e. no CR) and is considered to be clinically relevant (i.e. on average a 30% decrease in complications/events or worsening in functional status or 10-20% decrease in MACE or death). To detect this decrease and achieve 80% power (significance level of 5%) a group of 286 patients (143 in the Heart-ROCQ group and 143 in the Standard Care group) is needed. To incorporate a withdrawal of ±20% a total sample size of 350 is needed at baseline.

Interim analysis

An interim analysis will be conducted when 40% of the included patients have had the measurements one year after surgery. The study will be terminated prematurely when the primary outcome of one of the CR programs is obviously ($P<0.001$) different from the other CR program.

Primary and sensitivity analyses

All end points are primary analysed according to the 'intention-to-treat' principles and missing values are counted as worst-case score (nominal variables) or estimated using Maximum Likelihood estimation (interval variables). As supplementary analyses, the end points are analysed on a per-protocol principle with and without using imputation methods for missing values. In all analyses, a two-sided $p<0.05$ is considered statistically significant.

The total score of the primary endpoint will be handled as a continuous variable. All continuous variables will be analysed using linear mixed models to determine 'time x group' differences. Significant interactions will be further explored using the Bonferroni post-hoc test to determine differences between each time point. Non-parametric tests will be used if the assumptions

For peer review only - http://bmjopen.bmj.com/site/about/guidelines.xhtml

of normal distribution are violated. More information about the statistical methods and clinical relevance are written in the research protocol (ClinicalTrials.gov : NCT02984449).

Economic evaluation

For the evaluation of health-care utilization, standard prices published in the Dutch costs guidelines are used.(67) To compare the costs to quality adjusted life years (QALYs), QALYs are estimated with use of the EQ-5D-5L.(58) Utility values for the EQ-5D-5L are calculated based on the new Dutch tariff.(68) Results from the analysis are reported as an Incremental Cost Effectiveness Ratio (ICER), dividing the difference in effect, by the difference in costs. Bootstrap re-sampling will be performed, and cost-effectiveness acceptability curves will be plotted, to estimate the probability that the Heart-ROCQ program is cost effective when compared to standard care. A societal perspective is applied.

Study status

From May 2017 to December 2018, 75 patients were enrolled. In following years we expect that the enrolment will increase to 85 patients per year. The last patient is expected to be included in July 2021.

Patient and public involvement

In a pilot study (to be submitted) the patient satisfaction and the feasibility (in terms of accessibility, compliance, training load and safety) of the Heart-ROCQ program have been evaluated. Patients were very satisfied with the program and scoring it eight out of ten, therefore we did not change the content of the program. However, patients' rate of perceived exertion was generally quite low and no serious adverse events occurred during the bicycle training. For safety reasons the intensity was not increased. However, in order to estimate the maximum load more accurately and better tailor the program to the individual, we decided to change one of the stop criteria of the preoperative submaximal ergometry test from 70% to 90% of expected maximal heart rate. Furthermore, our outcomes are, among others, based on the reasons why patients recommended the program to other patients. For example, patients reported that their self-efficacy and physical capacity were

For peer review only - http://bmjopen.bmj.com/site/about/guidelines.xhtml

improved, so we added the CSE questionnaire and physical tests to objectively measure these outcomes. In this way the results were taken into account in the further development of the Heart-ROCQ program and the protocol of this study. The results of this trial will be distributed by various information channels (e.g. websites of cardiac patient organisations, social media). Two to three times a year we provide a newsletter about the progress and (in the end) the results of the study are sent to patients, who are interested.

For peer review only

For peer review only - http://bmjopen.bmj.com/site/about/guidelines.xhtml

Discussion

The Heart-ROCQ study is the first randomised clinical trial evaluating the effect of a combination of pre- and post-operative CR program compared to a post-operative CR program. Unlike the vast majority of CR programs in previous studies, the current program is multidisciplinary targeting different aspects of surgical outcomes in patients undergoing cardiac surgery. Because different aspects are targeted, a composite weighted score will better reflect the treatment benefits than a single outcome. Therefore, the primary end point is a composite end point of functional status, post-operative surgical complications, readmissions to the hospital and MACE. The components of the end point are of clinical importance to patients undergoing cardiac surgery and reflect a comprehensive representation of the recovery of the patient.

Both the ideas of the combined primary end point and the weighting of the individual components were derived from other studies.(41,69,70) Assigning different weights to the components was needed for more accurate comparison. Since the components are not equal in clinical importance, equal weights would lead to inaccurate statistical analysis(70). In the current study, the weighted score of HRQoL is lower (1 point instead of 2 points) to minimize bias due to patients' knowledge of group allocation. Therefore, improvements in quality of life are not counted in the primary end point to prevent bias in a positive direction. This also prevents that a score in quality of life and adverse event cancel each other out (e.g. when a patient experiences an improvement in Quality in Life [+2 points] and has a stroke [-2 points] then the total score is 0). The scores of all-cause mortality, stroke, myocardial infarction and revascularization are in line with the results showed by Tong and colleagues (2013)(70). A disadvantage of composite end points is that the effect may be driven by complications that occur with the greatest frequency.(71) Therefore, post-operative complications which occur frequently, such as atrial fibrillation, are evaluated separately as secondary end point. The primary end point is evaluated by an end-point committee, which is blinded to group allocation and consists of four independent cardiologists and cardio-thoracic surgeons.

Previous preoperative CR studies were primarily focused on short-term effects; only one preoperative CR study and a few post-operative CR studies have determined long-term

For peer review only - http://bmjopen.bmj.com/site/about/guidelines.xhtml

effects.(23,35,36,72) In contrast to previous preoperative CR studies, the Heart-ROCQ study sets out to investigate both short- and long term effects of the CR programs.(23,35,36) Due to the trends in, among others, increasing age, obesity, and physical inactivity, patients undergoing cardiac surgery are becoming more complex. The Heart-ROCQ program aims to address these issues, which make the program clinically relevant for all cardiac surgery patients. Therefore, we chose to include different types of cardiac surgery. Since different moderators and mediators are assessed before, during and after CR, we can explore which factors are associated with better outcomes and which working mechanisms contribute to its effectiveness. These findings may provide a more in-depth understanding of who benefits the most from CR in both the short and long term and the underlying mechanisms of CR, which are still not fully understood in patients undergoing cardiac surgery.(35) In addition, the present study is thought to considerably contribute to the evidence to further develop guidelines for clinical practice, especially regarding the preoperative CR program.(73)

The Heart-ROCQ program is expected to be cost-effective in the long-term, which is also of interest for policymakers and health care providers. Therefore, an economic evaluation is performed to assess the cost-effectiveness of CR, since little is known about the cost-effectiveness of CR.(64) A societal perspective of this economic evaluation is chosen, meaning that not only health-care costs, but also patient- and productivity related costs and benefits are taken into account. If the Heart-ROCQ program is proven to be effective, it might be advisable to include a supportive prevention-oriented waiting period prior to surgery as an integral part of the treatment for patients undergoing elective cardiac surgery. This implies a paradigm shift from curative care following cardiac surgery to an additional preventive care attitude before surgery. Extensions of rehabilitation options in, or in the vicinity of cardiac centres, will then be required.

The Heart-ROCQ study is the first randomised clinical trial comparing the effect of a combined pre- and post-operative CR program to a regular Dutch phase II post-surgery outpatient rehabilitation CR program in a population undergoing elective cardiac surgery. This study is expected to provide new understanding of the effectiveness and underlying working mechanisms of CR, and subsequently to improve value-based health care.

For peer review only - http://bmjopen.bmj.com/site/about/guidelines.xhtml

Acknowledgements

We thank E.J. Beens, M.J. Nijholt, Drs. A. Branderhorst, Dr. B. Dorhout (in memoriam), Dr. R. de Jong, H.A.M. Wasser, Dr. J. Brügemann, Dr. J.B. Wempe, Dr. J. Posma, Dr. C.A. Geluk, and Drs. M.L. Pentinga supporting the Heart-ROCQ study. In addition, we thank Drs. T.W. Waterbolk, Dr. R.G. Tieleman, and Dr. R.P.M. van Roosmalen for their participation in the end-point committee. We are grateful to all involved colleagues in the department of Cardiology, Cardio-thoracic Surgery, and Centre for Rehabilitation of the UMCG, OZG, MZH, and WZA for the pleasant collaboration and their help with executing the Heart-ROCQ study.

Declaration of interest

JH, SD, and MAM report grants from Edwards Lifesciences SA, Abbott (former St. Jude Medical Nederland B.V.), and 'Stichting Beatrixoord Noord-Nederland' related to this study. In addition, MAM reports consultancy from AtriCure, Getinge, and LivaNova. JF, WD, LHVvdW, MFR, FB, ICCvdH, MJLdJ, and PvdH have nothing to disclose.

Authors'contributions

Conception and design of the study: JH, FB, MJLdJ, MFR, WD, JF, LHVvdW, PvdH, and MAM; Methodology: JH, FB, SD, MJLdJ, MFR, and PvdH; Acquisition of data: JH, FB and SD; Writing – Original draft, tables, and figures: JH; Writing- Reviewing and Editing: FB, SD, MJLdJ, MFR, WD, ICCvdH, JF, LHVvdW, PvdH, and MAM; Supervision: MJLdJ, MFR, WD, JF, ICCvdH, LHVvdW, PvdH, and MAM; Funding Acquisition: JH, SD, JF, LHVvdW, PvdH, and MAM. All authors approved the final version of the manuscript.

For peer review only

For peer review only - http://bmjopen.bmj.com/site/about/guidelines.xhtml

References

1. GBD 2016 Causes of Death Collaborators. Global, regional, and national age-sex specific mortality for 264 causes of death, 1980–2016: a systematic analysis for the Global Burden of Disease Study 2016. Lancet. 2017;390(10100):1151–210.

2. Hulzebos EHJ, Van Meeteren NLU, De Bie RA, Dagnelie PC, Helders PJM. Prediction of postoperative pulmonary complications on the basis of preoperative risk factors in patients who had undergone coronary artery bypass graft surgery. Phys Ther. 2003;83(1):8–16.

3. Kassie GM, Nguyen TA, Kalisch Ellett LM, Pratt NL, Roughead EE. Preoperative medication use and postoperative delirium: a systematic review. BMC Geriatr. 2017 Dec 29;17(1):298.

4. Weymann A, Popov AF, Sabashnikov A, Ali-Hasan-Al-Saegh S, Ryazanov M, Tse G, et al. Baseline and postoperative levels of C-reactive protein and interleukins as inflammatory predictors of atrial fibrillation following cardiac surgery: a systematic review and meta-analysis. Kardiol Pol. 2018 Feb 16;76(2):440–51.

5. Koster S, Hensens AG, Schuurmans MJ, van der Palen J. Consequences of delirium after cardiac operations. Ann Thorac Surg. 2012 Mar;93(3):705–11.

6. Iribarne A, Chang H, Alexander JH, Gillinov AM, Moquete E, Puskas JD, et al. Readmissions after cardiac surgery: experience of the National Institutes of Health/Canadian Institutes of Health research cardiothoracic surgical trials network. Ann Thorac Surg. 2014 Oct;98(4):1274–80.

7. Villareal RP, Hariharan R, Liu BC, Kar B, Lee V-V, Elayda M, et al. Postoperative atrial fibrillation and mortality after coronary artery bypass surgery. J Am Coll Cardiol. 2004 Mar 3;43(5):742–8.

8. Hashemzadeh K, Dehdilani M, Dehdilani M. Postoperative Atrial Fibrillation following Open Cardiac Surgery: Predisposing Factors and Complications. J Cardiovasc Thorac Res. 2013;5(3):101–7.

9. Kosuma P, Wachirasrisirikul S, Jedsadayanmata A. Attributable Costs of Postoperative Atrial Fibrillation among Patients Undergoing Cardiac Surgery. Cardiol Res Pract. 2018;2018:1–5.

10. Findeisen A, Arefian H, Doenst T, Hagel S, Pletz MW, Hartmann M, et al. Economic burden of surgical site infections in patients undergoing cardiac surgery. Eur J Cardio-Thoracic Surg. 2018;0(8):1–7.

11. Ruiz-Núñez B, van den Hurk GHAM, de Vries JHM, Mariani MA, de Jongste MJL, Dijck-Brouwer DAJ, et al. Patients undergoing elective coronary artery bypass grafting exhibit poor pre-operative intakes of fruit, vegetables, dietary fibre, fish and vitamin D. Br J Nutr. 2015;(6):1–11.

12. Zittermann A, Kuhn J, Dreier J, Knabbe C, Gummert JF, Börgermann J. Vitamin D status and the risk of major adverse cardiac and cerebrovascular events in cardiac surgery. Eur Heart J. 2013;34(18):1358–64.

13. Kavanagh T, Mertens DJ, Hamm LF, Beyene J, Kennedy J, Corey P, et al. Prediction of long-term prognosis in 12 169 men referred for cardiac rehabilitation. Circulation. 2002;106(6):666–71.

14. van Laar C, Timman ST, Noyez L. Decreased physical activity is a predictor for a complicated recovery post cardiac surgery. Health Qual Life Outcomes. 2017;15(1):1–7.

15. Christian LM, Graham JE, Padgett DA, Glaser R, Kiecolt-Glaser JK. Stress and wound

For peer review only

healing. Neuroimmunomodulation. 2007;13(5–6):337–46.

16. Tully PJ, Baker RA. Depression, anxiety, and cardiac morbidity outcomes after coronary artery bypass surgery: a contemporary and practical review. J Geriatr Cardiol. 2012 Jun;9(2):197–208.

17. Kehler DS, Stammers AN, Tangri N, Hiebert B, Fransoo R, Schultz ASH, et al. Systematic review of preoperative physical activity and its impact on postcardiac surgical outcomes. BMJ Open. 2017;

18. Zhang YJ, Iqbal J, Van Klaveren D, Campos CM, Holmes DR, Kappetein AP, et al. Smoking is associated with adverse clinical outcomes in patients undergoing revascularization with PCI or CABG: The SYNTAX trial at 5-year follow-up. J Am Coll Cardiol. 2015;65(11):1107–15.

19. Mallik S, Krumholz HM, Zhen QL, Kasl S V., Mattera JA, Roumains SA, et al. Patients with depressive symptoms have lower health status benefits after coronary artery bypass surgery. Circulation. 2005;111(3):271–7.

20. Ghanta RK, Kaneko T, Gammie JS, Sheng S, Aranki SF. Evolving trends of reoperative coronary artery bypass grafting: An analysis of the Society of Thoracic Surgeons Adult Cardiac Surgery Database. J Thorac Cardiovasc Surg. 2013;145(2):364–72.

21. Kindo M, Hoang Minh T, Perrier S, Bentz J, Mommerot A, Billaud P, et al. Trends in isolated coronary artery bypass grafting over the last decade. Interact Cardiovasc Thorac Surg. 2017;24(1):71–6.

22. Carr T, Teucher U, Mann J, Casson AG. Waiting for surgery from the patient perspective. Psychol Res Behav Manag. 2009;2:107–19.

23. Arthur HM, Daniels C, McKelvie R, Hirsh J, Rush B. Effect of a preoperative intervention on preoperative and postoperative outcomes in low-risk patients awaiting elective coronary artery bypass graft surgery: A randomized, controlled trial. Ann Intern Med. 2000;133(4):253–62.

24. Van Der Peijl ID, Vliet Vlieland TPM, Versteegh MIM, Lok JJ, Munneke M, Dion RAE. Exercise therapy after coronary artery bypass graft surgery: A randomized comparison of a high and low frequency exercise therapy program. Ann Thorac Surg. 2004;77(5):1535–41.

25. Koopman C, Van Dis I, Vaartjes I, Visseren FLJ, Bots ML. Hart- en vaatziekten in Nederland 2014, cijfers over kwaliteit van leven, ziekte en sterfte. Harstichting. 2013. p. 192.

26. Takahashi T, Kumamaru M, Jenkins S, Saitoh M, Morisawa T, Matsuda H. In-patient step count predicts re-hospitalization after cardiac surgery. J Cardiol. 2015;66(4):286–91.

27. Cook DJ, Thompson JE, Prinsen SK, Dearani JA, Deschamps C. Functional recovery in the elderly after major surgery: Assessment of mobility recovery using wireless technology. Ann Thorac Surg. 2013;96(3):1057–61.

28. Kortebein P, Symons TB, Ferrando A, Paddon-Jones D, Ronsen O, Protas E, et al. Functional impact of 10 days of bed rest in healthy older adults. J Gerontol A Biol Sci Med Sci. 2008;63(10):1076–81.

29. Convertino V, Hung J, Goldwater D, DeBusk RF. Cardiovascular responses to exercise in middle-aged men after 10 days of bedrest. Circulation. 1982 Jan;65(1):134–40.

30. Chodzko-Zajko WJ, Proctor DN, Fiatarone Singh MA, Minson CT, Nigg CR, Salem GJ, et al. Exercise and physical activity for older adults. Med Sci Sports Exerc. 2009;41(7):1510–30.

31. BACPR. The British Association for Cardiovascular Prevention and Rehabilitation (BACPR)

For peer review only - http://bmjopen.bmj.com/site/about/guidelines.xhtml

standards and core components for cardiovascular disease prevention and rehabilitation, 2nd edition, 2012. 2012.

32. Bjarnason-Wehrens B, McGee H, Zwisler AD, Piepoli MF, Benzer W, Schmid J-P, et al. Cardiac rehabilitation in Europe: results from the European Cardiac Rehabilitation Inventory Survey. Eur J Cardiovasc Prev Rehabil. 2010;17(4):410–8.

33. De Vries H, Kemps HMC, Van Engen-Verheul MM, Kraaijenhagen RA, Peek N. Cardiac rehabilitation and survival in a large representative community cohort of Dutch patients. Eur Heart J. 2015;36:1519–28.

34. Anderson L, Taylor RS. Cardiac rehabilitation for people with heart disease: an overview of Cochrane systematic reviews. Cochrane database Syst Rev. 2014 Jan;12:CD011273.

35. Blokzijl F, Dieperink W, Keus F, Reneman MF, Mariani MA, van der Horst IC. Cardiac rehabilitation for patients having cardiac surgery: a systematic review. J Cardiovasc Surg (Torino). 2018 Apr 3;

36. Hulzebos EHJ, Smit Y, Helders PPJM, van Meeteren NLU. Preoperative physical therapy for elective cardiac surgery patients. Cochrane database Syst Rev. 2012 Jan;11:CD010118.

37. Sawatzky JA V, Kehler DS, Ready AE, Lerner N, Boreskie S, Lamont D, et al. Prehabilitation program for elective coronary artery bypass graft surgery patients: a pilot randomized controlled study. Clin Rehabil. 2014 Jan 23;28(7):648–57.

38. Vogelmeier CF, Criner GJ, Martinez FJ, Anzueto A, Barnes PJ, Bourbeau J, et al. Global Strategy for the Diagnosis, Management and Prevention of Chronic Obstructive Lung Disease 2017 Report. Respirology. 2017;22(3):575–601.

39. van Adrichem EJ, Meulenbroek RL, Plukker JTM, Groen H, van Weert E. Comparison of two preoperative inspiratory muscle training programs to prevent pulmonary complications in patients undergoing esophagectomy: a randomized controlled pilot study. Ann Surg Oncol. 2014 Jul;21(7):2353–60.

40. Van Dijk J. Multidisciplinaire Richtlijn Hartrevalidatie 2011. Tijdschr VOOR BEDRIJFS- EN Verzek. 2011;19(9):410–5.

41. Taylor AL, Ziesche S, Yancy C, Carson P, D'Agostino R, Ferdinand K, et al. Combination of Isosorbide Dinitrate an Hydralazine in Blacks with Heart Failure. N Engl J Med. 2004;351(20):2049–57.

42. Van der Zee KI, Sanderman R, Heyink JW, de Haes H, Van der Zee KI, Sanderman R, et al. Psychometric qualities of the RAND 36-Item Health Survey 1.0: a multidimensional measure of general health status. Int J Behav Med. 1996 Jan;3(2):104–22.

43. Wyrwich KW, Spertus JA, Kroenke K, Tierney WM, Babu AN, Wolinsky FD. Clinically important differences in health status for patients with heart disease: An expert consensus panel report. Am Heart J. 2004;147(4):615–22.

44. Thygesen K, Alpert JS, Jaffe AS, Simoons ML, Chaitman BR, White HD, et al. Third universal definition of myocardial infarction. Eur Heart J. 2012;33(20):2551–67.

45. Koster S, Hensens AG, Oosterveld FGJ, Wijma A, van der Palen J. The delirium observation screening scale recognizes delirium early after cardiac surgery. Eur J Cardiovasc Nurs. 2009;8(4):309–14.

46. Crapo RO, Casaburi R, Coates AL, Enright PL, MacIntyre NR, McKay RT, et al. ATS statement: Guidelines for the six-minute walk test. Am J Respir Crit Care Med.

For peer review only

For peer review only - http://bmjopen.bmj.com/site/about/guidelines.xhtml

2002;166(1):111–7.

47. Puthoff ML, Saskowski D. Reliability and responsiveness of gait speed, five times sit to stand, and hand grip strength for patients in cardiac rehabilitation. Cardiopulm Phys Ther J. 2013 Mar;24(1):31–7.

48. Douma KW, Regterschot GRH, Krijnen WP, Slager GEC, van der Schans CP, Zijlstra W. Reliability of the Q Force; a mobile instrument for measuring isometric quadriceps muscle strength. BMC Sports Sci Med Rehabil. 2016 Jan;8:4.

49. Katz S, Ford AB, Moskowitz RW, Jackson BA, Jaffe MW, Cleveland MA. Studies of Illness in the Aged. The Index of ADL: A Standardized Measure of Biological and Psychosocial Function. JAMA. 1963;185(12):914–9.

50. Reijneveld SA, Spijker J, Dijkshoorn H. Katz' ADL index assessed functional performance of Turkish, Moroccan, and Dutch elderly. J Clin Epidemiol. 2007;60(4):382–8.

51. Spitzer RL, Kroenke K, Williams JBW, Löwe B. A Brief Measure for Assessing Generalized Anxiety Disorder: the GAD-7. Arch Intern Med. 2006;166(10):1092–7.

52. Kroenke K, Spitzer RL, Williams JB. The PHQ-9: validity of a brief depression severity measure. J Gen Intern Med. 2001 Sep;16(9):606–13.

53. Löwe B, Unützer J, Callahan CM, Perkins AJ, Kroenke K. Monitoring depression treatment outcomes with the patient health questionnaire-9. Med Care. 2004 Dec;42(12):1194–201.

54. Craig CL, Marshall AL, Sjöström M, Bauman AE, Booth ML, Ainsworth BE, et al. International physical activity questionnaire: 12-Country reliability and validity. Med Sci Sports Exerc. 2003;35(8):1381–95.

55. Whitaker KM, Gabriel KP, Jacobs DR, Sidney S, Sternfeld B. Comparison of Two Generations of ActiGraph Accelerometers: The CARDIA Study. Med Sci Sport Exerc. 2018;50(6):1333–40.

56. Bouwmans C, Hakkaart-van Roijen L, Koopmanschap M, Krol M, Severens H BW. Medical Cost Questionnaire (iMCQ). Rotterdam: iMTA, Erasmus Universiteit Rotterdam. 2013 [cited 2016 May 1]. Available from: https://www.imta.nl/

57. Bouwmans C, Krol M, Severens H, Koopmanschap M, Brouwer W, Roijen LH Van. The iMTA Productivity Cost Questionnaire: A Standardized Instrument for Measuring and Valuing Health-Related Productivity Losses. Value Heal. 2015;18(6):753–8. Available from: http://dx.doi.org/10.1016/j.jval.2015.05.009

58. EuroQol Group. What is EQ-5D? 2014 [cited 2016 Nov 3]. Available from: http://www.euroqol.org/

59. Sullivan MD, LaCroix AZ, Russo J, Katon WJ. Self-efficacy and self-reported functional status in coronary heart disease: a six-month prospective study. Psychosom Med. 1998;60(4):473–8.

60. Moss-Morris R, Weinman J, Petrie K. The revised illness perception questionnaire (IPQ-R). Psychol Heal. 2002;17(1):1–16.

61. Morgan K, Villiers-Tuthill A, Barker M, McGee H. The contribution of illness perception to psychological distress in heart failure patients. BMC Psychol. 2014 Nov 28;2(1):50.

62. Meetbaar Beter Boek 2015 [cited 2016 Apr 25]. p. 60. Available from: http://www.meetbaarbeter.com/documents/meetbaar-beter-boeken/

63. Engblom E, Korpilahti K, Hämäläinen H, Rönnemaa T, Puukka P. Quality of life and return to

For peer review only

For peer review only - http://bmjopen.bmj.com/site/about/guidelines.xhtml

work 5 years after coronary artery bypass surgery. Long-term results of cardiac rehabilitation. J Cardiopulm Rehabil. 1997 Jan;17(1):29–36.

64. Heran BS, Chen JM, Ebrahim S, Moxham T, Oldridge N, Rees K, et al. Exercise-based cardiac rehabilitation for coronary heart disease. Cochrane database Syst Rev. 2011 Jul 6;(7):CD001800.

65. Serruys PW, Morice M, Kappetein AP, Colombo A, Holmes DR, Mack MJ, et al. Percutaneous Coronary Intervention versus Coronary-Artery Bypass Grafting for Severe Coronary Artery Disease. N Engl J Med. 2009;360:961–72.

66. Jolliffe JA, Rees K, Taylor RS, Thompson D, Oldridge N, Ebrahim S. Exercise-based rehabilitation for coronary heart disease. Cochrane database Syst Rev. 2001 Jan;(1):CD001800.

67. Zorginstituut Nederland. Kostenhandleiding: Methodologie van kostenonderzoek en referentieprijzen voor economische evaluaties in de gezondheidszorg. 2015.

68. Versteegh MM, Vermeulen KM, Evers SMAA, de Wit GA, Prenger R, Stolk EA. Dutch Tariff for the Five-Level Version of EQ-5D. Value Health. 2016 Jun;19(4):343–52.

69. de Vries AE, de Jong RM, van der Wal MH, Jaarsma T, van Dijk RB, Hillege HL. The value of INnovative ICT guided disease management combined with Telemonitoring in OUtpatient clinics for Chronic Heart failure patients. Design and methodology of the IN TOUCH study: a multicenter randomised trial. BMC Health Serv Res. 2011;11(1):167.

70. Tong BC, Huber JC, Ascheim DD, Puskas JD, Ferguson Jr B, Blackstone EH, et al. Weighting Composite Endpoints in Clinical Trials: Evidence for the Heart Team. 2013;94(6):1908–13.

71. Anker SD, Schroeder S, Atar D, Bax JJ, Ceconi C, Cowie MR, et al. Traditional and new composite endpoints in heart failure clinical trials: facilitating comprehensive efficacy assessments and improving trial efficiency. Eur J Heart Fail. 2016 May;18(5):482–9.

72. Stammers a. N, Kehler DS, Afilalo J, Avery LJ, Bagshaw SM, Grocott HP, et al. Protocol for the PREHAB study--Pre-operative Rehabilitation for reduction of Hospitalization After coronary Bypass and valvular surgery: a randomised controlled trial. BMJ Open [Internet]. 2015;5(3):e007250.

73. Engelman DT, Ben Ali W, Williams JB, Perrault LP, Reddy VS, Arora RC, et al. Guidelines for Perioperative Care in Cardiac Surgery: Enhanced Recovery after Surgery Society Recommendations. JAMA Surg. 2019;12:1–12.

For peer review only

For peer review only - http://bmjopen.bmj.com/site/about/guidelines.xhtml

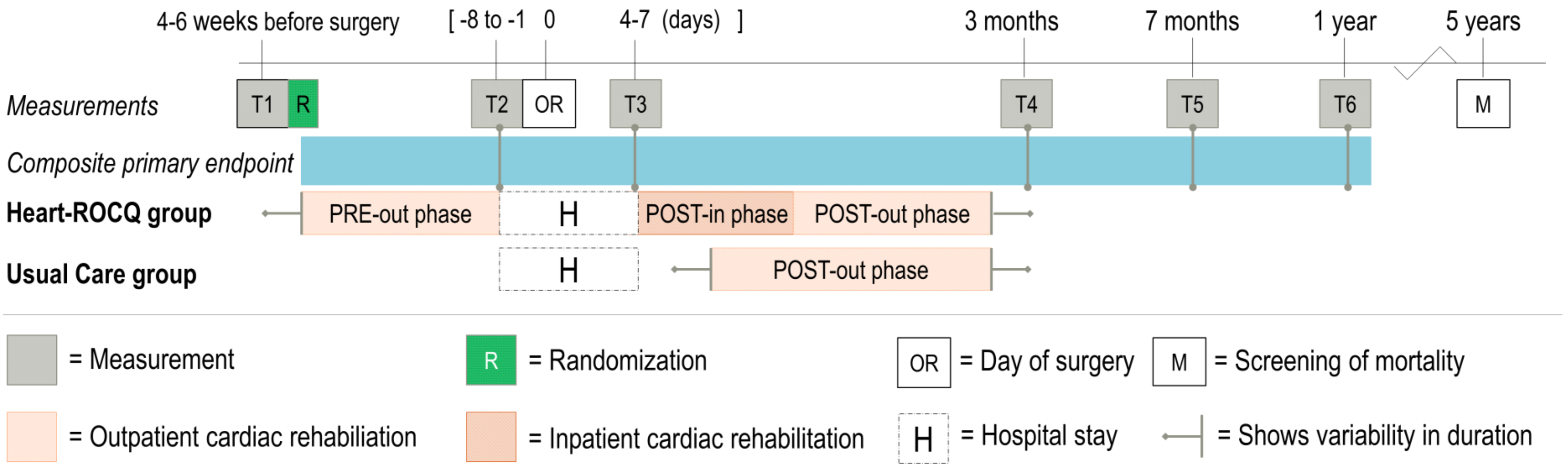

Figure 1: Research design of the Heart-ROCQ PROBE trial. The phases of both cardiac rehabilitation programs and the measurements are shown relative to the moment of surgery.

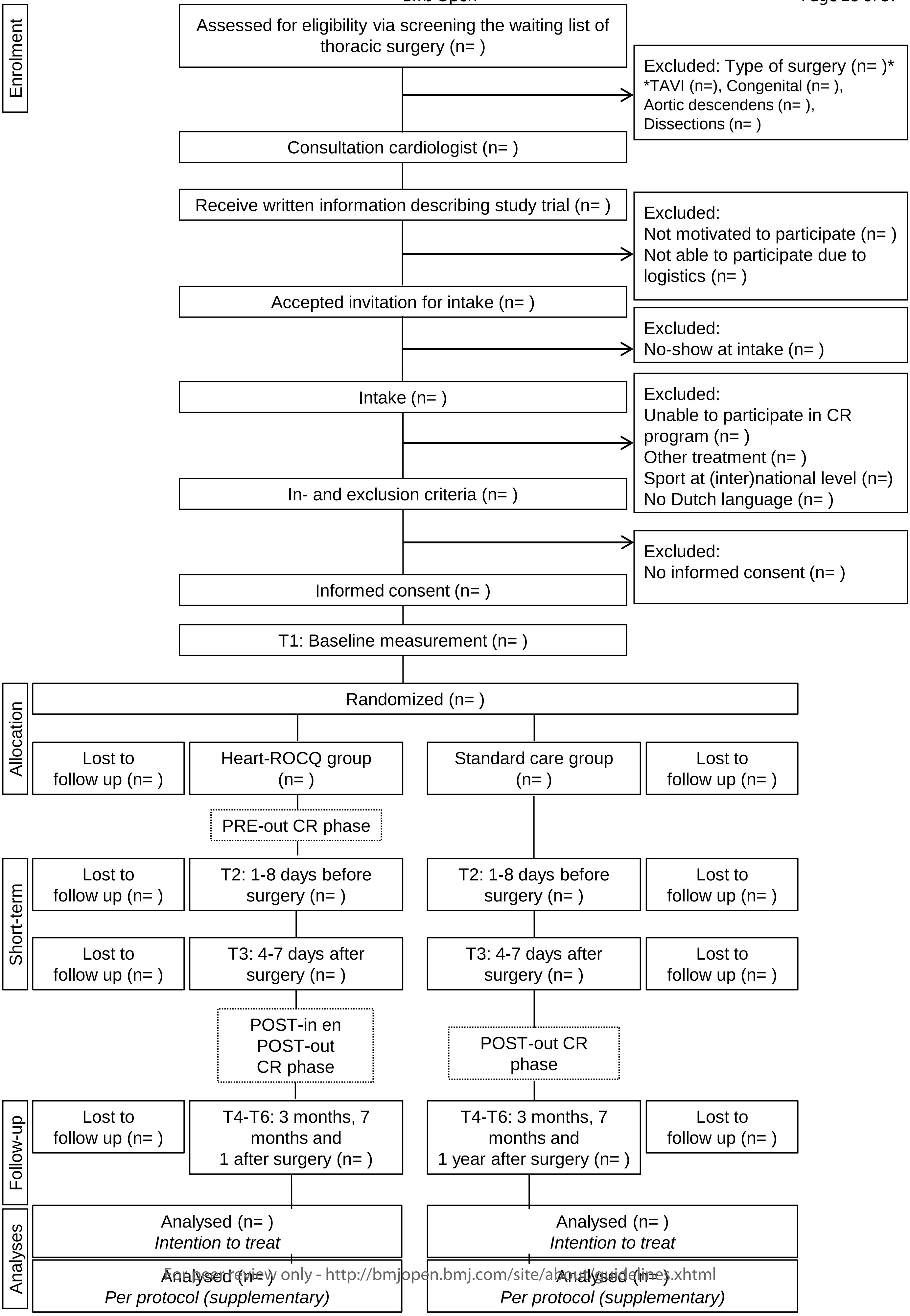
Enrolment
Assessed for eligibility via screening the waiting list of thoracic surgery (n=)
Excluded: Type of surgery (n=)*
*TAVI (n=), Congenital (n=), Aortic descendens (n=), Dissections (n=)
Consultation cardiologist (n=)
Receive written information describing study trial (n=)
Excluded:
Not motivated to participate (n=)
Not able to participate due to logistics (n=)
Accepted invitation for intake (n=)
Excluded:
No-show at intake (n=)
Intake (n=)
Excluded:
Unable to participate in CR program (n=)
Other treatment (n=)
Sport at (inter)national level (n=)
No Dutch language (n=)
In- and exclusion criteria (n=)
Excluded:
No informed consent (n=)
Informed consent (n=)
T1: Baseline measurement (n=)
Allocation
Randomized (n=)
Lost to follow up (n=)
Heart-ROCQ group (n=)
Standard care group (n=)
Lost to follow up (n=)
PRE-out CR phase
Short-term
Lost to follow up (n=)
T2: 1-8 days before surgery (n=)
T2: 1-8 days before surgery (n=)
Lost to follow up (n=)
Lost to follow up (n=)
T3: 4-7 days after surgery (n=)
T3: 4-7 days after surgery (n=)
Lost to follow up (n=)
POST-in en POST-out CR phase
POST-out CR phase
Follow-up
Lost to follow up (n=)
T4-T6: 3 months, 7 months and 1 after surgery (n=)
T4-T6: 3 months, 7 months and 1 year after surgery (n=)
Lost to follow up (n=)
Analyses
Analysed (n=)
Intention to treat
Analysed (n=)
Intention to treat
Analysed (n=)
Per protocol (supplementary)
Analysed (n=)
Per protocol (supplementary)

SUPPLEMENTARY INFORMATION - DESCRIPTION OF THE HEART-ROCQ PROGRAM

PRE-out phase – An outpatient cardiac rehabilitation phase during the waiting period
Three times per week, a minimum of three weeks

Physical therapy

Aims
- To maintain or improve patients physical capacity before surgery
- Patient learns to apply the stress-strain training principles
- To optimize pulmonary muscle strength
- Patient knows which breathing and coughing techniques should be used after surgery to prevent pulmonary complications

When needed:
- Patient learns to recognize body signals and boundaries.
- Patient is able to exercise despite of possible kinesiophobia

Type of exercise	Frequency	Intensity	Monitoring
IMT	3 × p / wk	- 6 cycles of 6 repetitions,<br>- rest periods of resp. 60, 45, 30, 15 and 5 s.<br>- 60-80% of maximal inspiratory pressure[65]	- Week 1: each training ↑ intensity with 10%<br>- Intensity ↑ with 5% if RPE <5[1]
Aerobic cycle	3 × p / wk	- 25 min. at RPE 3[1]<br>- Interval training will be given, if the patient is not able to perform endurance training.	- First training: 50% of POpeak<br>- Load ↑ if RPE< 3[1]<br>- Interval training: guided by complaints and RPE of 3
Resistance training	3 × p / wk	- 1-3 cycles of 10-15 repetitions<br>- Rest: 30-60 s.<br>- 50-80% of estimated 1RM<br>- On six fitness apparatuses	- First training: 6-10 RM per fitness apparatus
Body awareness	1 × p / 2 wks	- 30 min. breathing and relaxation techniques	Not Applicable
Group education	Two sessions	- Basic training principles<br>- Forced expiration, huff and cough techniques	Not Applicable

Dietary advice[2]

Aims
- To maintain or improve patients nutritional status
- Patient knows which nutrients are important focusing on the surgery
- Patients knows the importance of food in relation to cardiovascular disease and cardiovascular risk management

Type	Frequency	Content
Intake interview	One session	- Anamnesis about nutritional status
Counselling	On indication	- Individual sessions according to existing guidelines
Group education	One session	- 60 min., cardiovascular risk factors and dietary intake

Psychological guidance[2]

Aims
- To optimize mental status of the patients before surgery
- Patient has made a start with the awareness of cardiovascular risk factors

Intake interview	One session	- Anamnesis about mental status
Counselling	On indication	- Individual sessions according to evidence-based treatment protocols dependent on mental problems of patient[3]
Group education	One session	- Coping with psychological tension/stress, to process the mental trauma of cardiac surgery, and risk factors

No-smoking consultation *(For patients who smoke)*

Aim
- Patient gives up smoking during the waiting time before surgery

Intake interview & counselling	One session per wk	- 30 min., individual session based on existing guidelines

IMT: Inspiratory muscle training; RPE: Rate perceives exertion; POpeak: Maximum power output achieved during submaximal Ergometry test. [1]On a Borgscale 0-10; [2]Involvement of partner/relatives during group and individual sessions; [3] like cognitive behavioural therapy, eye movement desensitization and reprocessing (EMDR), acceptance and commitment therapy etc.

For peer review only

For peer review only - http://bmjopen.bmj.com/site/about/guidelines.xhtml

POST-in phase – An inpatient cardiac rehabilitation phase
Starting 4-7 days after surgery, duration of three weeks, weekends at home

Physical therapy

Aim

- To recover patients physical capacity
- Patient performs breathing and coughing techniques to prevent pulmonary complications
- Patient mobilize and can perform activities of daily living independently
- Patient knows about risk factors according to physical activity, knows the ACSM recommendations (moderate-intensity cardiorespiratory exercise training for ≥30min/day ≥5 days/week or vigorous-intensity cardiorespiratory exercise training for ≥20 min/day on ≥3 days/week or a combination[66]) and makes a plan to apply the ACSM recommendations in his own life
- Patient works on personal goals

Type of exercise	Frequency	Intensity	Monitoring
Individual therapy	2 × p / day	- Practice at transfers, walking, and climbing stairs<br>- Very light mobilizing exercises for upper extremity	- During the first 2 days of this phase.<br>- Extended, if patient is not able to participate in the group sessions.
Individual therapy	2 × p / wk	- Attention to personal goals	- Week 2 and 3
IMT[2]	3 × p / wk: 2 × under supervision, 1 × without supervision	- 6 cycles of 6 repetitions,<br>- rest periods of resp. 60, 45, 30, 15 and 5 s.<br>- intensity of 60-80% of maximum inspiratory pressure[65]	- First training: 50% of resistance of last preoperative training<br>- Intensity ↑ with 5% if RPE <5[1]<br>- IMT stops when resistance of preoperative training is reached
Aerobic cycle[2]	1 × p / day	- Week 1: duration of 5-20 min. at light intensity (RPE 2[1])<br>- Week 2 and 3: Work up to 25 min. at moderate intensity (RPE 3[1])<br>- Interval training will be given, if the patient is not able to perform endurance training.	- First training: at 50% of power output of last preoperative training<br>- Load ↑ if RPE< 2 à 3[1]<br>- Interval training: guided by complaints and RPE of 3.
Resistance training[2]	3-4 × p / wk	- 3 cycles of 15-20 repetitions<br>- Rest periods of 30-60 seconds<br>- On six fitness apparatuses	- First training: 50% of resistance of last preoperative training for LE and 25% for UE.<br>- Gradual build up to 50-80% van 1RM based on RPE 3[1]
Body awareness[2]	1 × p / wk	- 30 min. breathing and relaxation techniques	Not Applicable

Dietary advice[3]

Aim

- To maintain or improve patients nutritional status
- Patient knows which nutrients are important focusing on the recovery of surgery
- Patients knows the importance of food in relation to cardiovascular disease and cardiovascular risk management

Type	Frequency	Content
Intake interview	One session	- Anamnesis about nutritional status post-surgery
Counselling	Sessions on indication	- Treatment according to existing guidelines
Group education	One session	- 90 min., skills needed to maintain a healthy life style

Psychological guidance[3]

Aim

- Patient start to process the mental trauma of cardiac surgery and the consequences of it
- Patient has self-management competence to maintain a healthy life style
- Patient and partner/relatives are able to support each other in the processing process

Intake interview	One session	- Anamnesis about mental status post-surgery
Intake interview & counselling	Sessions on indication	- Individual sessions according to evidence-based treatment protocols dependent on mental problems of patient[4]
Group education	One session	- 90 min., processing the cardiac surgery: possible reactions en consequences, coping with stress and healthy life style

IMT: Inspiratory muscle training; RPE: Rate perceives exertion; LE: Lower extremities; UE: Upper extremities. [1]On a Borgscale 0-10; [2]Group sessions start from day three of the inpatient phase or when patient is able to perform in the group activities; [3]Involvement of partner/relatives during group and individual sessions; [4] like cognitive behavioural therapy, eye movement desensitization and reprocessing (EMDR), acceptance and commitment therapy etc.

For peer review only

For peer review only - http://bmjopen.bmj.com/site/about/guidelines.xhtml

**POST-in phase – An inpatient cardiac rehabilitation phase (continuation)**<br>*Starting 4-7 days after surgery, duration of three weeks, weekends at home*		
**No-smoking consultation**[1] *(For patients who smoke)*		
Aim<br>- Patient is still motivated to stop smoking or patient is motivated to give up smoking (when patient did not give up smoking before cardiac surgery.		
Intake interview & counseling	1 × p / wk	- 30 min., individual sessions based on
Group education	One session	- 60 min., general information about smoking addiction and support from fellow smokers
**Return to work consultation**[1] *(for patients who are employed)*		
Aim<br>- Patient is informed about laws and regulation for illness, social security contributions, and medical examinations<br>- Patient knows their rights and obligations, the rights and obligations of the employer, the working conditions agency, the employee insurance agency, and re-integration companies<br>- Patient received tools to return adequately back to work (knows positive and negative factors that can impact the re-integration)		
Group education	One session	- 60 min., laws and regulation for illness, procedures, roles, rights and obligations of different involved persons, communication to involved persons (e.g. colleagues, employer), working during rehabilitation, positive and negative factors regarding to return to work
Counselling	Sessions on indication	- Individual sessions with labour consultant dependent on problems of patient

[1]Involvement of partner/relatives during group and individual sessions.

For peer review only

**POST-out phase – An outpatient cardiac rehabilitation phase**<br>*Starting on Tuesday after discharge of the POST-in phase, two times per week, four weeks*			
**Physical therapy**			
Aim<br>- Patient has optimized his/her physical capacity<br>- Patient knows his/her boundaries and limitations<br>- Patient knows about cardiovascular risk factors according to physical activity, knows the ACSM recommendations (moderate-intensity cardiorespiratory exercise training for ≥30min/day ≥5 days/week or vigorous-intensity cardiorespiratory exercise training for ≥20 min/day on ≥3 days/week or a combination[65]) and makes a plan to apply the ACSM recommendations in his own life<br>- Patient resumes his/her work or hobbies<br>- Patient experiences pleasure during exercise<br>- Patients achieves their personal goals			
**Type of exercise**	**Frequency**	**Intensity**	**Monitoring**
Aerobic cycle	2 × p / wk	Depending on trainability of patient<br>- 25 min. at 50-80% HRR or RPE 5[1]	- Work up to 25 minutes at moderate intensity (RPE 3[1]), when patient was not able to do it in POST-in phase<br>- Load ↑ if RPE< 5[1] or HRR <50-80%
Resistance training	2 × p / wk	UE:<br>- 3-4 cycles of 10-15 repetitions<br>- Rest: 30-60 s.<br>- 50-80% of estimated 1RM<br>- On six fitness apparatuses<br>LE:<br>- Focus on mobilization in week 1 & 2<br>- Focus on strength and endurance in week 3 & 4	- UE: Load ↑ if RPE< 5[1]<br>- LE: Under guidance of complaints
Sport and games	1 × p / wk	Focus on:<br>- Experiencing pleasure during exercise, regaining trust and handling boundaries.<br>- Exploring different types of sports and knowing the possibilities after CR	
Swimming	1 × p / wk		
Education	1 × p / wk	- Training principles of POST-out phase and a repetition of ACSM recommendations<br>- Awareness of exercise after CR; making a plan to exercise after CR<br>- Explanation of the results of the exercise test<br>- Discussing the plan to exercise, share experiences to expand exercise in home situation	
**Dietary advice, psychological guidance, no-smoking consultation, and return to work consultation**			
Individual sessions are continued when aims are not achieved			

RPE: Rate perceives exertion; HRR: Heart rate reserve; LE: Lower extremities; UE: Upper extremities; ACSM: American College of Sports Medicine; CR: Cardiac Rehabilitation. [1]On a Borgscale 0-10; [2]Involvement of partner/relatives during group and individual sessions.

For peer review only

STANDARD PROTOCOL ITEMS: RECOMMENDATIONS FOR INTERVENTIONAL TRIALS

SPIRIT 2013 Checklist: Recommended items to address in a clinical trial protocol and related documents*

Section/item	Item No	Description	Addressed on page number
**Administrative information**			
Title	1	Descriptive title identifying the study design, population, interventions, and, if applicable, trial acronym	______1_______
Trial registration	2a	Trial identifier and registry name. If not yet registered, name of intended registry	______3, 6_____
	2b	All items from the World Health Organization Trial Registration Data Set	______3, 6_____
Protocol version	3	Date and version identifier	________6______
Funding	4	Sources and types of financial, material, and other support	______7_______
Roles and responsibilities	5a	Names, affiliations, and roles of protocol contributors	______1, 19___
	5b	Name and contact information for the trial sponsor	______1_______
	5c	Role of study sponsor and funders, if any, in study design; collection, management, analysis, and interpretation of data; writing of the report; and the decision to submit the report for publication, including whether they will have ultimate authority over any of these activities	______7_______
	5d	Composition, roles, and responsibilities of the coordinating centre, steering committee, endpoint adjudication committee, data management team, and other individuals or groups overseeing the trial, if applicable (see Item 21a for data monitoring committee)	______7, 8, 10, 13

For peer review only

Introduction

Background and rationale	6a	Description of research question and justification for undertaking the trial, including summary of relevant studies (published and unpublished) examining benefits and harms for each intervention	___4, 5______
	6b	Explanation for choice of comparators	___4, 5_______
Objectives	7	Specific objectives or hypotheses	___5_________
Trial design	8	Description of trial design including type of trial (eg, parallel group, crossover, factorial, single group), allocation ratio, and framework (eg, superiority, equivalence, noninferiority, exploratory)	___6, 7, ______

Methods: Participants, interventions, and outcomes

Study setting	9	Description of study settings (eg, community clinic, academic hospital) and list of countries where data will be collected. Reference to where list of study sites can be obtained	___6________
Eligibility criteria	10	Inclusion and exclusion criteria for participants. If applicable, eligibility criteria for study centres and individuals who will perform the interventions (eg, surgeons, psychotherapists)	___7________
Interventions	11a	Interventions for each group with sufficient detail to allow replication, including how and when they will be administered	___9________
	11b	Criteria for discontinuing or modifying allocated interventions for a given trial participant (eg, drug dose change in response to harms, participant request, or improving/worsening disease)	___NA______
	11c	Strategies to improve adherence to intervention protocols, and any procedures for monitoring adherence (eg, drug tablet return, laboratory tests)	___13______
	11d	Relevant concomitant care and interventions that are permitted or prohibited during the trial	___7_______
Outcomes	12	Primary, secondary, and other outcomes, including the specific measurement variable (eg, systolic blood pressure), analysis metric (eg, change from baseline, final value, time to event), method of aggregation (eg, median, proportion), and time point for each outcome. Explanation of the clinical relevance of chosen efficacy and harm outcomes is strongly recommended	___10-14___
Participant timeline	13	Time schedule of enrolment, interventions (including any run-ins and washouts), assessments, and visits for participants. A schematic diagram is highly recommended (see Figure)	____6-14___

For peer review only - http://bmjopen.bmj.com/site/about/guidelines.xhtml

Sample size	14	Estimated number of participants needed to achieve study objectives and how it was determined, including clinical and statistical assumptions supporting any sample size calculations	___15_______
Recruitment	15	Strategies for achieving adequate participant enrolment to reach target sample size	___7, 8, 16___

Methods: Assignment of interventions (for controlled trials)

Allocation:

Sequence generation	16a	Method of generating the allocation sequence (eg, computer-generated random numbers), and list of any factors for stratification. To reduce predictability of a random sequence, details of any planned restriction (eg, blocking) should be provided in a separate document that is unavailable to those who enrol participants or assign interventions	___7, 8______
Allocation concealment mechanism	16b	Mechanism of implementing the allocation sequence (eg, central telephone; sequentially numbered, opaque, sealed envelopes), describing any steps to conceal the sequence until interventions are assigned	___7, 8______
Implementation	16c	Who will generate the allocation sequence, who will enrol participants, and who will assign participants to interventions	____7, 8_____
Blinding (masking)	17a	Who will be blinded after assignment to interventions (eg, trial participants, care providers, outcome assessors, data analysts), and how	____7, 8_____
	17b	If blinded, circumstances under which unblinding is permissible, and procedure for revealing a participant's allocated intervention during the trial	___NA________

Methods: Data collection, management, and analysis

Data collection methods	18a	Plans for assessment and collection of outcome, baseline, and other trial data, including any related processes to promote data quality (eg, duplicate measurements, training of assessors) and a description of study instruments (eg, questionnaires, laboratory tests) along with their reliability and validity, if known. Reference to where data collection forms can be found, if not in the protocol	__11-14_______
	18b	Plans to promote participant retention and complete follow-up, including list of any outcome data to be collected for participants who discontinue or deviate from intervention protocols	__NA_________

Data management	19	Plans for data entry, coding, security, and storage, including any related processes to promote data quality (eg, double data entry; range checks for data values). Reference to where details of data management procedures can be found, if not in the protocol	__13_________
Statistical methods	20a	Statistical methods for analysing primary and secondary outcomes. Reference to where other details of the statistical analysis plan can be found, if not in the protocol	___15, 16_____
	20b	Methods for any additional analyses (eg, subgroup and adjusted analyses)	___15________
	20c	Definition of analysis population relating to protocol non-adherence (eg, as randomised analysis), and any statistical methods to handle missing data (eg, multiple imputation)	____15_______
**Methods: Monitoring**			
Data monitoring	21a	Composition of data monitoring committee (DMC); summary of its role and reporting structure; statement of whether it is independent from the sponsor and competing interests; and reference to where further details about its charter can be found, if not in the protocol. Alternatively, an explanation of why a DMC is not needed	___13________
	21b	Description of any interim analyses and stopping guidelines, including who will have access to these interim results and make the final decision to terminate the trial	___15________
Harms	22	Plans for collecting, assessing, reporting, and managing solicited and spontaneously reported adverse events and other unintended effects of trial interventions or trial conduct	___13________
Auditing	23	Frequency and procedures for auditing trial conduct, if any, and whether the process will be independent from investigators and the sponsor	___13________
**Ethics and dissemination**			
Research ethics approval	24	Plans for seeking research ethics committee/institutional review board (REC/IRB) approval	___6________
Protocol amendments	25	Plans for communicating important protocol modifications (eg, changes to eligibility criteria, outcomes, analyses) to relevant parties (eg, investigators, REC/IRBs, trial participants, trial registries, journals, regulators)	___6_________

For peer review only - http://bmjopen.bmj.com/site/about/guidelines.xhtml

Consent or assent	26a	Who will obtain informed consent or assent from potential trial participants or authorised surrogates, and how (see Item 32)	__7, 8_______
	26b	Additional consent provisions for collection and use of participant data and biological specimens in ancillary studies, if applicable	__7, 8_______
Confidentiality	27	How personal information about potential and enrolled participants will be collected, shared, and maintained in order to protect confidentiality before, during, and after the trial	__13_________
Declaration of interests	28	Financial and other competing interests for principal investigators for the overall trial and each study site	__19_________
Access to data	29	Statement of who will have access to the final trial dataset, and disclosure of contractual agreements that limit such access for investigators	__13_________
Ancillary and post-trial care	30	Provisions, if any, for ancillary and post-trial care, and for compensation to those who suffer harm from trial participation	__13_________
Dissemination policy	31a	Plans for investigators and sponsor to communicate trial results to participants, healthcare professionals, the public, and other relevant groups (eg, via publication, reporting in results databases, or other data sharing arrangements), including any publication restrictions	___6_________
	31b	Authorship eligibility guidelines and any intended use of professional writers	___6_________
	31c	Plans, if any, for granting public access to the full protocol, participant-level dataset, and statistical code	___NA_______
**Appendices**			
Informed consent materials	32	Model consent form and other related documentation given to participants and authorised surrogates	___13________
Biological specimens	33	Plans for collection, laboratory evaluation, and storage of biological specimens for genetic or molecular analysis in the current trial and for future use in ancillary studies, if applicable	___NA________

*It is strongly recommended that this checklist be read in conjunction with the SPIRIT 2013 Explanation & Elaboration for important clarification on the items. Amendments to the protocol should be tracked and dated. The SPIRIT checklist is copyrighted by the SPIRIT Group under the Creative Commons "Attribution-NonCommercial-NoDerivs 3.0 Unported" license.

For peer review only - http://bmjopen.bmj.com/site/about/guidelines.xhtml
